# ChemMORT: an automatic ADMET optimization platform using deep learning and multi-objective particle swarm optimization

**DOI:** 10.1093/bib/bbae008

**Published:** 2024-02-20

**Authors:** Jia-Cai Yi, Zi-Yi Yang, Wen-Tao Zhao, Zhi-Jiang Yang, Xiao-Chen Zhang, Cheng-Kun Wu, Ai-Ping Lu, Dong-Sheng Cao

**Affiliations:** School of Computer Science, National University of Defense Technology, Changsha 410073, Hunan, PR China; Xiangya School of Pharmaceutical Sciences, Central South University, Changsha 410013, Hunan, P. R. China; Xiangya School of Pharmaceutical Sciences, Central South University, Changsha 410013, Hunan, P. R. China; School of Computer Science, National University of Defense Technology, Changsha 410073, Hunan, PR China; Xiangya School of Pharmaceutical Sciences, Central South University, Changsha 410013, Hunan, P. R. China; School of Computer Science, National University of Defense Technology, Changsha 410073, Hunan, PR China; State Key Laboratory of High-Performance Computing, Changsha 410073, Hunan, PR China; Institute for Advancing Translational Medicine in Bone and Joint Diseases, School of Chinese Medicine, Hong Kong Baptist University, Hong Kong SAR, P. R. China; Xiangya School of Pharmaceutical Sciences, Central South University, Changsha 410013, Hunan, P. R. China; Institute for Advancing Translational Medicine in Bone and Joint Diseases, School of Chinese Medicine, Hong Kong Baptist University, Hong Kong SAR, P. R. China

**Keywords:** ADMET evaluation, lead optimization, substructure modification, deep learning, inverse QSAR, reversible molecular representation, particle swarm optimization

## Abstract

Drug discovery and development constitute a laborious and costly undertaking. The success of a drug hinges not only good efficacy but also acceptable absorption, distribution, metabolism, elimination, and toxicity (ADMET) properties. Overall, up to 50% of drug development failures have been contributed from undesirable ADMET profiles. As a multiple parameter objective, the optimization of the ADMET properties is extremely challenging owing to the vast chemical space and limited human expert knowledge. In this study, a freely available platform called Chemical Molecular Optimization, Representation and Translation (ChemMORT) is developed for the optimization of multiple ADMET endpoints without the loss of potency (https://cadd.nscc-tj.cn/deploy/chemmort/). ChemMORT contains three modules: Simplified Molecular Input Line Entry System (SMILES) Encoder, Descriptor Decoder and Molecular Optimizer. The SMILES Encoder can generate the molecular representation with a 512-dimensional vector, and the Descriptor Decoder is able to translate the above representation to the corresponding molecular structure with high accuracy. Based on reversible molecular representation and particle swarm optimization strategy, the Molecular Optimizer can be used to effectively optimize undesirable ADMET properties without the loss of bioactivity, which essentially accomplishes the design of inverse QSAR. The constrained multi-objective optimization of the poly (ADP-ribose) polymerase-1 inhibitor is provided as the case to explore the utility of ChemMORT.

## INTRODUCTION

The journey of discovering a new drug candidate and shepherding it through clinical trials and onto the market is time-consuming, fraught with difficulties, inordinately expensive, and prone to failure, which typically costs 15 years and $12–15 million for successfully developing a clinical candidate [1]. Since the key to improving pharmaceutical productivity is to boost the efficiency of discovering drug candidates entering clinical trials, several revolutionary technologies have been used for accelerating drug candidate development, such as combinatorial chemistry, X-ray crystallography, high-throughput screening (HTS) and virtual screening (VS) [2–4]. However, despite the inventiveness and rapid advances witnessed in HTS and VS [5–7], the attrition rate during the early drug candidate discovery is still as high as 75%, even for those experienced global pharmaceutical companies [8, 9]. It is estimated that up to 50% of failures are contributed from the deficiency in absorption, distribution, metabolism, elimination, and toxicity (ADMET) properties, which reaffirms the importance of optimizing ADMET properties during drug discovery campaigns [10]. The ADMET optimization could be viewed as an extremely challenging multi-parameter optimization task, which aims to improve multiple ADMET properties while avoid the reduction of biological potency [11, 12].

With large-scale investigations of deep learning (DL) in molecular representations [13, 14], it is found that the chemical space learned by deep neural networks represents several advantages: smooth, continuous, unique and expressive, which could efficiently benefit molecular optimization [15]. Recently, a method proposed by Gomez-Bombarelli *et al.* [16] further illustrates the above advantages through the application of variational autoencoder, with the additional feature of reversibility. This autoencoder comprises two neural networks: an encoder and a decoder. The encoder network transforms input Simplified Molecular Input Line Entry System (SMILES) strings into a lower-dimensional representation, commonly referred to as the latent space. Conversely, the decoder network maps the points from this latent space back to SMILES sequences. To achieve the encoding of higher-level molecular features rather than the syntactic concepts or repetitive patterns of the sequence, the reconstruction task is transformed into a translation task, by translating one molecular representation to another syntactically different one [17, 18]. Several researches have shown that the models trained with enumerated SMILES sequences own more advantages in data augmentation and important molecular feature learning than those only trained with one variant (e.g. canonical SMILES) [17, 19]. In addition, it has been observed that latent space vectors showed superior performance in molecular similarity analysis and Quantitative Structure–Activity Relationship (QSAR) modeling than autoencoder-derived vectors and the models built using the Extended Connectivity Fingerprints (ECFP4) fingerprints, suggesting that they have increased relevant to biological activities and physicochemical properties [17]. Due to high reversibility and information enrichment, latent space vectors are highly recommended for inverse QSAR problems. For the navigation of the optimization tasks for multiple molecular properties, the application of latent space vectors needs to be combined with efficient optimization strategy and necessary structural constraint, thus effectively avoiding the drop of target potency [20].

Considering the importance of ADMET optimization in drug discovery, here, a freely available platform called Chemical Molecular Optimization, Representation and Translation (ChemMORT) is developed for the optimization of multiple ADMET endpoints (https://cadd.nscc-tj.cn/deploy/chemmort/). ChemMORT contains three basic modules: SMILES Encoder, Descriptor Decoder and Molecular Optimizer, which provide the representation, translation and optimization functions, respectively. Based on the training of 17 million enumerated SMILES strings, SMILES Encoder can generate the 512-dimensional molecular representation, and Descriptor Decoder is able to translate the above representation to the corresponding molecular structure with high accuracy. Based on the reversible molecular representation and particle swarm optimization (PSO) strategy, Molecular Optimizer can effectively accomplish ADMET optimization tasks while preserving the potency of the optimized molecules through necessary similarity and substructure constraint. To evaluate the utility of ChemMORT, the constrained multi-objective optimization of the poly (ADP-ribose) polymerase-1 (PARP-1) inhibitor was provided as the case. It is believed that through the rational application of ChemMORT, researchers can discover potent drug candidates with improved ADMET profiles.

## MATERIALS AND METHODS

### Neural translation model

In this study, inspired by human language neural network translation models, a sequence-to-sequence (seq2seq) model was trained based on the SMILES notation for chemical space exploration. It turns enumerated SMILES notation into a fixed-length vector representation in the encoder and turns this fixed-length vector into the Canonical SMILES of the molecule in the decoder, where the fixed-length vector will be used as the connection between structure modification and property optimization [18]. Generally, recurrent neural networks are used as the backend of the seq2seq model, which often bring the vanishing or exploding gradient problems. To avoid it, three stacked *Gate Recurrent Unit* (GRU) layers were used in both the encoder and decoder networks. In addition, a fully connected layer (*information bottleneck*) with 512 units and hyperbolic tangent activation function is used as the final layer of the encoder to generate a 512-dimensional latent representation. Through information bottleneck, it can capture the most statistically salient features about molecular structures, which ensures the accuracy of translation and the efficiency for property prediction. The decoder takes the latent representation as an input and feeds it into a similar three stacked aforementioned GRU layers with 1024, 512 and 256 units. The input of the decoder to each time step is the output of the preceding time step and the embedding of the ground truth. In the training phase, the output of the decoder transfers to the ground truth to calculate the cross-entropy loss and conduct the gradient update. In the prediction phase, the beam search algorithm [21], a heuristic search algorithm that explores the best combination of words by expanding the most promising node in a limited set, is used in the model to iteratively predict each character until a complete sequence is generated. An internal database with 1.7 million accessible molecules was used to validate the reliability and generalization ability of the model. All the molecules were randomly divided into a training set (1.53 million molecules) and a test set (0.17 million molecules) with a ratio of 9:1. Every molecule was represented by 10 different enumerated SMILES strings for encoding. A previous study has already proved that training the encoder with enumerated SMILES strings and the decoder with Canonical SMILES is able to achieve a better balance between translation correction rate and chemical space breadth [17].

### ADMET prediction model

In order to construct credible ADMET-related prediction models for molecular optimization, a large and high quality ADMET dataset containing basic information and experimental values were collected from the ChEMBL, EPA and DrugBank databases, and all the molecules in the dataset were prepared by molecular operating environment (MOE, version 2016) [22–25]. Finally, around 30 000 entries, covering logD7.4, LogS, Caco-2, MDCK cells, Plasma protein binding rate (PPB), AMES toxicity, human ether-a-go-go-related gene (hERG) toxicity, hepotoxicity and median lethal dose (LD50), were obtained for ADMET evaluation [23, 24, 26–29]. The source and information about the ADMET dataset are summarized in Table S1, see Supplementary Data available online at http://bib.oxfordjournals.org/. Based on the combination of the calculated 512-dimensional vectors and XGBoost algorithm, nine high-quality ADMET prediction models were constructed for the evaluation and guidance of molecular optimization. In addition, three calculated properties, including SlogP, quantitative estimate of drug-likeness (QED) score and synthetic accessibility (SA) score, were also included in ChemMORT for more comprehensive evaluation of molecular suitability [30–32].

### Scoring scheme

To provide a comprehensive perspective of multi-parameter optimization task, the scoring scheme was applied for a qualitative evaluation of the desirability of the optimized molecule. Based on the recommended value range (Table S2, see Supplementary Data available online at http://bib.oxfordjournals.org/), the customized aim range and the actual property value for individual scores will be: 1 for the value in the optimal range, (0, 1) for the value out of the optimal range but in the recommended value range, and 0 for the value out of the recommended value range. Considering the different requirements in different optimization tasks, the individual scaled score components with customized weights will be combined according to the importance of different features in the whole task. The Final Score (eq 2) will be presented as the weighted average score of all scaled scores, where a low value corresponds to undesirable optimization and a high value indicates an acceptable optimization. 


(1) \begin{equation*} F=\frac{\sum_{i=1}^j\left({S}_i\cdotp{W}_i\right)}{\sum_{i=1}^j{W}_i} \end{equation*}

where $j$ is the number of the objective functions used for optimization, ${S}_i$ represents the desirability of the objective function $i$ of the optimized molecule, and ${W}_i$ corresponds to the priority of the objective function $i$ in this task.

### Particle swarm optimization

Based on the continuous presentation and scoring scheme, the PSO [33], a stochastic optimization method that mimics swarm intelligence to find an optimal point in a search space, was applied to explore the optimized molecules with desirable properties [34–37]. Inspired by social behavior of bird flocking or fish schooling, the PSO consists of individuals for space searching, which utilizes and communicates the information gained during their search. During this process, each particle in the swarm is defined by their position $x$ and velocity $v$, where the scoring scheme $f$ is applied for the detection of the potential surface of the search space. The movement of the *i-*th particle at iteration step *k* is influenced by the historical best point of itself: 


(2) \begin{equation*} {x}_i^{best}= argmaxf\left({x}_i^k\right) \end{equation*}

as well as the overall historical best point of the swarm:


(3) \begin{equation*} {x}^{best}= argmaxf\left({x}_i^{best}\right) \end{equation*}

After each iteration, each particle will update its velocity and position based on the collected information and its status:


(4) \begin{equation*} {v}_i^{k+1}=w{v}_i^k+{c}_1{r}_1\left({x}_i^{best}-{x}_i^k\right)+{c}_2{r}_2\left({x}^{best}-{x}_i^k\right) \end{equation*}

(5) \begin{equation*} {x}_i^{k+1}={x}_i^k+{v}_i^{k+1} \end{equation*}

where ${c}_1$ and ${c}_2$ are the constants that weight the contribution of the individual experience versus the swarm experience; ${r}_1$ and ${r}_2$ are the random numbers drawn from independent uniform distributions between 0 and 1; the inertia weight $w$ is a constant that controls the momentum of the particle from the previous iteration. In this work, the position of the particle is initialized by the output of the encoder.

### Webserver development

ChemMORT was developed by using Python 3.7, Django 2.2, Tensorflow 1.14.0, SQLite 3, celery 4.4.7, RabbitMQ 3.6.10 and RDKit 2019.03.1. It was a Django task, which is deployed on a high-performance Nginx Web server of Ubuntu 18.04.4 LTS via the application of uWSGI. ChemMORT applied the MVT (model, view and template)design pattern, including three layers: model layer, view layer and template layer. The model layer interfaces to the SQLite3 database which was applied for model construction, upload file storing and property prediction. The view layer contains the main logic code, which was used for providing access to the prediction models, handling file upload and download, and manipulating multi-prediction tasks. The template layer was applied for the presentation of the front-end pages, including result visualization, page rendering, document integration, etc. The browser compatibility testing is shown in Table S6, see Supplementary Data available online at http://bib.oxfordjournals.org/.

## RESULTS AND DISCUSSION

### ChemMORT workflow

**Figure 1:**
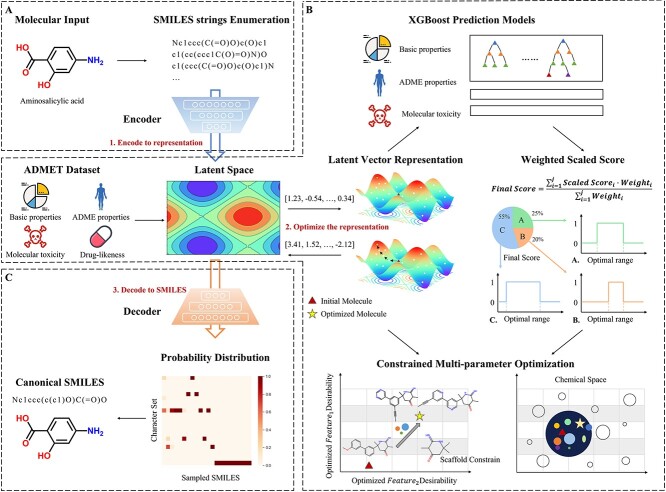
The workflow of ChemMORT.

The ChemMORT protocol is presented as a workflow in Figure 1. As shown in Figure 1, there are three main modules in ChemMORT: SMILES Encoder, Descriptor Decoder and Molecular Optimizer, which refer to the functions of descriptor calculation, molecular translation and ADMET optimization, respectively.

SMILES Encoder: Three molecular input types are supported by the SMILES Encoder module: inputting SMILES strings, drawing molecules from the editor, and uploading file (*.sdf/*.csv/*.txt). After the molecular preparation process, the corresponding 512-dimension vectors will be calculated based on the well-trained encoder network. In the following page, the Summary and Result block will present the overview of the results and the detailed information about the SMILES strings, molecular graphs and 512-dimension vectors. The calculated descriptors and related SMILES strings can be saved as .csv file.

Descriptor Decoder: In this function, users can upload a 512-dimensional vector (between −1 and 1), which latter will be back-engineered to the corresponding uniform canonical SMILES string through the application of decoder network. It should be noted that owing to the character-by-character nature of the SMILES representation and the fragility of its internal syntax, an arbitrary combination may lead to the output of invalid or failed molecules. After calculation, the summary and result block are provided for the overview of the translated SMILES information, where the results can also be downloaded as .csv file. Owing to the advantage of reversibility, the combination of SMILES Encoder and Descriptor Decoder possesses the ability to deal with the inverse design problem, which is the key point of lead optimization.

Molecular Optimizer: Molecular optimization is a complex and multi-objective task, which needs to balance bioactivity, pharmacokinetic profile and therapeutic safety. To achieve this goal, the Molecular Optimizer module is provided with the integration of reversible molecular representation, credible QSAR models, necessary structural constraint and multi-objective PSO strategy, which follows the principle of inverse QSAR methodology. Firstly, users are required to input the job information and the SMILES string of the molecule that needs to be optimized. Twelve credible objective functions covering basic molecular properties, synesthetic accessibility, drug-likeness, absorption, distribution and toxicity were provided for property optimization. To retain the efficacy and novelty of initial optimized molecule, the *Similarity Constrain* and *Substructure Constrain* functions were applied for the definition of the starting point and the annotation of important active motif, respectively [38]. The application of the *Similarity Constrain* function enables the setting of the distance limitation between the generated molecule and the reference molecule based on the ECFP4 fingerprint and Tanimoto similarity metric, while the application of the *Substructure Constrain* function highlights the importance of bioactivity motif. All the above functions are allowed to set the weights according to their importance, which later will be applied to the scaled score for optimization navigation and comprehensive evaluation. Owing to the different requirements for optimal molecules, users can adjust the iteration steps and the number of the top desirable compounds in each iteration step. After submission, the optimization job will be calculated in the background. Users can obtain the optimized result from the email or the access of Queue page with the input of job token. The final result includes the information about the starting molecule and optimized molecules, of which the latter one provides the detailed table about the SMILES, the structural graph, selected optimized property values and the final score. Based on the combination of user-defined property value range, specific function weights and the optimized property value, the final score is a comprehensive desirability index of the optimized molecules, and it can quantitatively indicate the desirability and quality of the optimized molecule in the specified optimization task.

### Neural translation model training

**Figure 2:**
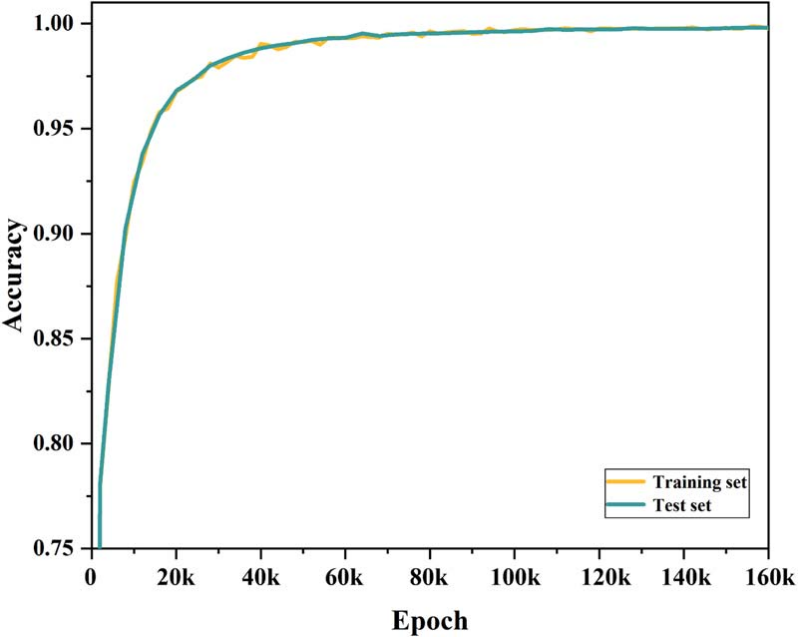
The translation accuracy for the training set and test set during the 160 k training steps.

A multi-layer gate recurrent unit network, including input dropout, bottleneck layer and Gaussian noise term, was employed for training and application. The model was trained until convergence, using a batch size of 64, dropout ratio of 0.15 and embedding noise of 0.05. As shown in Figure 2, the translation accuracy for the training set and test set first increased rapidly, but after a point, it became stable and almost unchanged. The final average single character accuracy values for both the training and test sets reached 99.8%, indicating the proportion of correctly predicted characters to the total predicted length. This achievement underscores the reliability and credibility of this seq2seq model. It also indicated that the important feature of the molecule has already been encoded in the latent space, resulting in a potentially powerful molecular descriptor for further ADMET prediction and optimization task.

### ADMET predictive model validation

Based on the 512-dimensional descriptors calculated by the encoder network, 9 ADMET-related prediction models, including logD7.4, AMES, Caco-2, MDCK, PPB, LogS, hERG, hepatoxicity and LD50, were constructed with the XGBoost algorithm. All the datasets were divided into the training set and test set according to the chemical space distribution computed by the ‘Diverse training set split’ module in ChemSAR, where 75% compounds were used as the training set and the remaining 25% as the test set [39]. The prediction performance of the ADMET prediction models and related parameters are summarized in Table 1 and Table S3, see Supplementary Data available online at http://bib.oxfordjournals.org/, respectively.

**Table 1:** The performance of ADMET prediction models in ChemMORT

ADMET regression model performance										
Property	Description	Algorithm^*^	5-Fold CV			Test set			Data information	
			RMSE	MAE	R^2^	RMSE	MAE	R^2^	Training set	Test set
logD7.4	Log of the octanol/water distribution coefficient at pH 7.4.	XGBoost	0.562 ± 0.009	0.428 ± 0.13	0.834 ± 0.005	0.555 ± 0.010	0.426 ± 0.007	0.840 ± 0.004	773	258
logS	Log of Solubility	XGBoost	0.842 ± 0.084	0.592 ± 0.056	0.839 ± 0.029	0.823 ± 0.026	0.572 ± 0.009	0.862 ± 0.011	4116	1104
Caco-2	Caco-2 Permeability	XGBoost& Data Augment	0.328 ± 0.004	0.245 ± 0.005	0.728 ± 0.011	0.332 ± 0.007	0.244 ± 0.004	0.718 ± 0.019	886	296
MDCK	MDCK Permeability	XGBoost& Data Augment	0.322 ± 0.034	0.235 ± 0.021	0.644 ± 0.057	0.323 ± 0.022	0.232 ± 0.011	0.650 ± 0.041	912	228
PPB	Plasma Protein Binding	XGBoost	0.154 ± 0.010	0.106 ± 0.007	0.691 ± 0.025	0.152 ± 0.003	0.104 ± 0.002	0.691 ± 0.016	1368	454
ADMET classification model performance										
Property	Description	Algorithm^*^	5-Fold CV			Test set			Data information	
			Accuracy	Sensitivity	AUC	Accuracy	Sensitivity	AUC	Training set	Test set
AMES	The probability to be positive in Ames test.	XGBoost	0.810 ± 0.016	0.838 ± 0.014	0.889 ± 0.013	0.813 ± 0.007	0.835 ± 0.013	0.888 ± 0.004	7514	1905
hERG	The probability to be hERG Blocker	XGBoost	0.800 ± 0.036	0.820 ± 0.068	0.857 ± 0.053	0.814 ± 0.026	0.841 ± 0.042	0.854 ± 0.032	392	263
hepatoxicity	The probability of owning liver toxicity	XGBoost	0.700 ± 0.026	0.701 ± 0.030	0.764 ± 0.030	0.729 ± 0.016	0.732 ± 0.019	0.794 ± 0.015	2208	502
LD_50_	LD50 of acute toxicity	XGBoost	0.741 ± 0.045	0.742 ± 0.128	0.833 ± 0.033	0.765 ± 0.007	0.764 ± 0.015	0.848 ± 0.007	5917	1480
QED	quantitative estimate of drug-likeness	n/a	n/a	n/a	n/a	n/a	n/a	n/a	n/a	n/a
SlogP	Log of the octanol/water partition coefficient, based on an atomic contribution model	n/a	n/a	n/a	n/a	n/a	n/a	n/a	n/a	n/a

*Data Augment refers to the application of enumerated SMILES in model construction.

As shown in Table 1, it can be observed that most models have high and stable performance in both the 5-fold cross validation and the test set prediction. For the regression models, the average values of RMSE and R2 are 0.442 and 0.747 for the 5-fold cross validation, respectively, and 0.437 and 0.752 for the test set, respectively. For the classification models, the average values of accuracy and AUC are 0.763 and 0.836 for the 5-fold cross validation, respectively, and 0.780 and 0.846 for the test set, respectively. Such results not only proved the credibility of the ADMET prediction models, but also indicated the effectiveness and utility of the latent representations calculated by ChemMORT. In addition, the combination of the encoding and decoding networks ensures the reversibility of the latent representations, which enables the ADMET prediction models to navigate molecular optimization.

### Constrained multi-objective optimization

PARP-1 is a critical DNA repair enzyme in the base excision repair pathway. Inhibitors of PARP-1 provide a new type of anticancer drugs that selectively kill cancer cells by targeting homologous recombination repair defects [40, 41]. However, most PARP-1 inhibitors suffer from the deficiency of poor aqueous solubility, which has severely disrupted the applicability value [42]. Therefore, the optimization of more hydrophilic but still potent PARP-1 inhibitors for cancer therapy is quite necessary.

**Figure 3:**
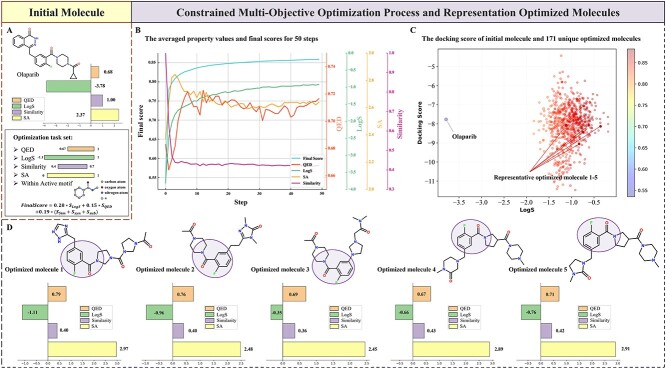
The information about the initial molecule, optimization process and representative optimized molecules. (A) The introduction of the initial molecule Olaparib and related optimization task set; (B) The averaged values of the final score, QED, LogS, SA and similarity during the 50-step optimization; (C) The docking score, final score and LogS value of Olaparib and 171 unique optimized molecules; (D) Five representative optimized molecules with their property information.

The approved drug Olaparib is selected as the initial molecule for further optimization, which is an efficient PARP-1 inhibitor possessing IC_50_ of 0.9 nmol but solubility of only 0.0601 mg/mL (logS of −3.8). During this multi-parameter optimization task, the solubility, QED and SA of the molecule are selected as the aim properties. In addition, to ensure the potency of the PARP-1 inhibitor, the bioactivity motif and similarity constraint are also used (Figure 3A). This optimization task is repeated 100 times and 50 iterations are conducted for the PSO optimization each time. The detailed information about the privilege function section and corresponding weight, the different properties over the course of the optimization, and the final representative optimized molecules are depicted in Figure 3.

As shown in Figure 3B, ChemMORT is consistently able to optimize the initial molecule with respect to the defined multi-parameter properties. Clearly, the LogS value and the final score first increased rapidly, but after a point, it became stable and almost unchanged. For QED and SA, according to the dense interval settings, they tend to fluctuate between 0.70 and 0.72, and between 2.5 and 2.7, respectively. For similarity, the tendency is totally converse, with a stable value of 0.40 for the later optimized molecules. It is not surprising since it is a process for molecular optimization. Besides, according to the constraint of bioactivity motif, the optimized molecule is not far from the initial molecule, indicating the importance of structural constraints. Finally, 171 unique optimized molecules with higher bioactivity and better water-solubility than the initial molecule are generated after 100 optimization cycles (Table S4, see Supplementary Data available online at http://bib.oxfordjournals.org/). As shown in Figure 3C, the solubility and final desirable score of the optimized molecules are much higher than those of the initial molecule. One of the main reasons is the substitution of 1,2-dihydrophthalazine to a more polar function group, such as 1H-1,2,4-triazole, piperazine and imidazolidine with N,N-dimethylacetamide, thus strengthening the hydration of compounds and promoting the thermodynamic process of dissolution [43]. Though the ability to optimize molecular pharmacokinetic properties often comes at the price of limited bioactivity to target, but with the application of molecular docking, it is found that most optimized molecules have rather high docking scores, which indicated preliminary guarantee of their potency (Table S4, see Supplementary Data available online at http://bib.oxfordjournals.org/). Such privileged results have a close relationship with the implementation of the constraints of the essential bioactivity motif and the similarity threshold to the initial molecule. Five representative optimized molecules with their property information are provided in Figure 3D. All of them possess high structural similarity to Olaparib and other approved PARP1 drugs, which successfully replicated the ideation of medicinal chemists during lead optimization.

**Figure 4:**
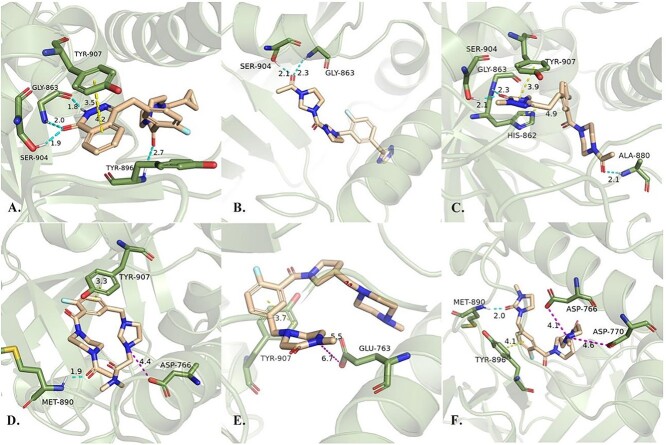
The interactions between the generated active molecules with PARP1. Figures A-F correspond to the binding mode of Olaparib and the optimized molecules 1 ~ 5, respectively. The hydrogen-bonding, π-π stacking interactions and charged interactions are indicated by different colors.

We then investigated the detailed interactions between the optimized molecules and the PARP-1 target (PDB ID: 4L6S). The predicted binding modes of Olaparib and the optimized molecules are presented in Figure 4. As shown in Figure 4, most of the optimized molecules have several key interactions, such as the H-bonding networks with Gly863 and Ser904 and the π-π stacking with Tyr907, which are known as the key binding patterns between PARP-1 inhibitors and PARP-1.

**Figure 5:**
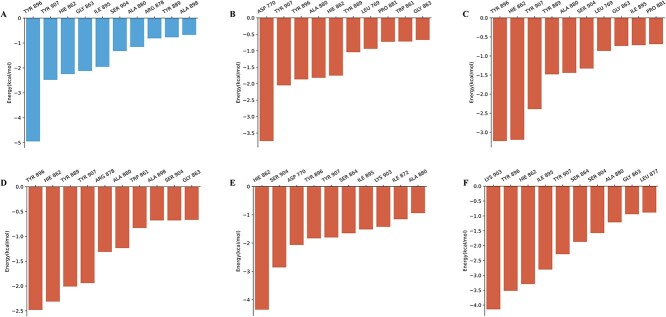
The top 10 residues contributing to the binding free energies of the generated active molecules. Figures A-F correspond to the binding modes of Olaparib and the optimized molecules 1 ~ 5, respectively.

To further ensure the utility and potency of these optimized molecules, molecular dynamics (MD) simulation was used to characterize the protein–ligand interactions of the optimized molecules [44]. Specifically, the AMBER ff19SB force field [45] and the General AMBER Force Field 2 [46] were used to parameterize the system. The conformation of the protein–ligand complex was generated using Vina [47]. Prior to the MD simulation, two-step minimizations, heating and equilibration were performed. The minimized system was heated to 298.15 K and increased to atmospheric pressure. Then, 20 ns production MD simulation was carried out for each complex in the ensemble with a time step of 2 fs. The structural root-mean-square-deviations (RMSDs) of the backbone atoms (C, C_a_ and N) of the protein relative to the initial structures were examined as a function of time (Figure S1). As can be seen in the plot, all the systems were stable during the 20-ns MD simulations. The RMSDs of PARP-1 in complex with five optimized molecules showed almost the same RMSDs with Olaparib. Additionally, we uniformly extracted 100 frames from the trajectory of the last 2 nanoseconds. Subsequently, we performed molecular mechanics generalized Born surface area (MM/GBSA) calculations and residue energy decomposition using AmberTools2023 [48]. The predicted binding free energies listed in Table S5, see Supplementary Data available online at http://bib.oxfordjournals.org/, also indicate that the optimized molecule 3 (−56.772 kcal/mol), 4 (−58.949 kcal/mol) and 5 (−58.831 kcal/mol) were almost at the same level of the binding affinity for Olaparib (−52.130 kcal/mol). To further identify the key residues related to the binding process, the free energy contributions of the top 10 residues at the binding site were estimated. As shown in Figure 5, Tyr896, Tyr907, Hie862 and Tyr907 are the most important residues for most optimized molecules, which is in high agreement with the residue contributions for PARP1 inhibitors (Figure 5A). Such analysis indicated that the reasonable application of privileged motif and similarity constraint is necessary to maintain molecular bioactivity during lead optimization [49]. Although this multi-parameter optimization task consists of many different and partially conflicting individual objectives, such as aqueous solubility, activity, SA and structural constraints, ChemMORT is consistently able to find some molecules in the vast chemical space that meets the desirable ranges for all of the defined ADMET objectives within the guarantee of target bioactivity.

## CONCLUSION

The success of a drug is determined not only by good efficacy and specificity, but also the acceptable ADMET properties. However, the optimization of the lead molecules is a multiple parameter optimization problem, covering potency, selectivity, pharmacokinetics features and safety, which is extremely challenging owing to the vast and discrete drug-like chemical space and limited knowledge from experimental transformation. Therefore, to break through this bottleneck, ChemMORT is developed for the multiple ADMET property optimization of drug candidates through the application of NMT, credible ADMET prediction models and multi-objective PSO strategy. Three modules are included in ChemMORT: SMILES Encoder, Descriptor Decoder and Molecular Optimizer, which provide the representation, translation and optimization functions, respectively. The constrained multi-objective optimization of PARP-1 inhibitors has indicated the successful match of ChemMORT to chemist design, which has successfully optimized ADMET properties of the initial molecule with the preservation of target binding affinity. It is anticipated that the future is bright for ADMET property optimization of lead molecules with the rational application of ChemMORT.

**Key Points**

- ChemMORT is a web-based integrated tool that learns molecular representations based on an encoder-decoder neural network architecture. It enhances network performance using SMILES enumeration and conducts multi-objective optimization of molecular properties through particle swarm optimization algorithms. ChemMORT can effectively optimize undesirable ADMET properties without compromising bioactivity, thereby achieving reverse QSAR design at its core.
- ChemMORT has been meticulously designed and optimized for its functional modules to enhance user experience. It supports batch upload and download functionalities. Users can define promising and desirable molecules based on their own criteria. The optimizer also provides substructure and similarity constraints, allowing users to freely adjust the importance of each property for highly customizable molecular optimization.
- The constrained multi-objective optimization of the PARP-1 inhibitor is provided as the case to explore the utility of ChemMORT.

## Supplementary Material

supplementary_materials_bbae008

## Data Availability

The project is available in the GitHub address (https://github.com/antwiser/ChemMORT).

## ABBREVIATIONS

ADMET, absorption, distribution, metabolism, elimination, and toxicity; QSAR, quantitative structure–activity relationship; DL, deep learning; SMILES, simplified molecular input line entry specification; HTS, high-throughput screening; VS, virtual screening; ECFP4, extended connectivity fingerprints; RNNs, recurrent neural networks; hERG, human ether-a-go-go-related gene; PPB, plasma protein binding rate; PSO, particle swarm optimization; PARP-1, Poly (ADP-ribose) polymerase-1; MD, molecular dynamics; RMSDs, root-mean-square-deviations;

## Author Biographies

**Jia-cai Yi** is a graduate student in the School of Computer Science, National University of Defense Technology. His researches focus on artificial intelligence for drug discovery.

**Zi-yi Yang** is a graduate student at Xiangya School of Pharmaceutical Sciences, Central South University. Her researches focus on screening for frequent hit compounds.

**Wen-Tao Zhao** is currently a professor in the School of Computer Science, National University of Defense Technology, China. His research interests include network performance optimization, information processing, and machine learning.

**Zhi-Jiang Yang** is a graduate student at Xiangya School of Pharmaceutical Sciences, Central South University. His researches focus on the development of cheminformatics tools.

**Xiao-Chen Zhang** is a Ph.D. student in the School of Computer Science, National University of Defense Technology, China. His researches focus on the development of cheminformatics tools.

**Cheng-Kun Wu** is currently an associate professor research fellow student in Institute for Quantum Information & State Key Laboratory of High Performance Computing, College of Computer Science and Technology, National University of Defense Technology. His researches focus on Systems Biology, High-Performance Computing, Pattern Recognition, Machine Learning, and Data Mining.

**Ai-Ping Lu** is a professor at the Hong Kong Baptist University and has long been engaged in basic and new drug discovery and development of Chinese medicine for the treatment of rheumatoid arthritis and oncology, as well as the research of aptamer-targeted delivery of innovative drugs.

**Dong-Sheng Cao** is currently an associate professor in the Xiangya School of Pharmaceutical Sciences, Central South University, China. His research interests can be found at the website of his group: http://www.scbdd.com.
