# Supplementary material for: ChemMORT: an automatic ADMET optimization platform using deep learning and multi-objective particle swarm optimization
Source: Brief Bioinform. 2024 Feb 20;25(2):bbae008. doi: 10.1093/bib/bbae008 (PMC10883642; doi:10.1093/bib/bbae008)
Supplement: supplementary_materials_bbae008 [file supplementary_materials_bbae008.zip › supplementary_materials_bbae008/Table S1.docx]

**Table S1.** The source and information about ADMET datasets

| **Property** | **Category** | **Data** | **Source** |
| --- | --- | --- | --- |
| logD7.4 | Basic property | 1031 | ADMETlab (<http://admet.scbdd.com/home/index/>) |
| logS | Basic property | 5220 | ADMETlab (<http://admet.scbdd.com/home/index/>) |
| Caco-2 | Absorption | 1182 | ADMETlab (<http://admet.scbdd.com/home/index/>) |
| MDCK | Absorption | 1140 | ADMETlab (<http://admet.scbdd.com/home/index/>) |
| PPB | Distribution | 1822 | ADMETlab (<http://admet.scbdd.com/home/index/>) |
| AMES | Toxicity | 9419 | ADMETlab (<http://admet.scbdd.com/home/index/>) |
| hERG | Toxicity | 655 | ADMETlab (<http://admet.scbdd.com/home/index/>) |
| hepatoxicity | Toxicity | 2710 | Mol. Pharmaceutics 2019, 16, 393−408 |
| LD50 | Toxicity | 7397 | ADMETlab (<http://admet.scbdd.com/home/index/>) |
| QED | Comprehensive evaluation | n/a | Nature Chem 4, 2012, 90–98 |
| SlogP | Basic property | n/a | J. Chem. Inf. Comput. Sci. 1999, 39, 5, 868–873 |
