# Supplementary material for: ChemMORT: an automatic ADMET optimization platform using deep learning and multi-objective particle swarm optimization
Source: Brief Bioinform. 2024 Feb 20;25(2):bbae008. doi: 10.1093/bib/bbae008 (PMC10883642; doi:10.1093/bib/bbae008)
Supplement: supplementary_materials_bbae008 [file supplementary_materials_bbae008.zip › supplementary_materials_bbae008/Table S2.docx]

**Table S2.** The systematic value range in ChemMORT

| **Property** | **Name** | **Recommended value range** |
| --- | --- | --- |
| Basic property | logD7.4 | [-3,8] |
| Basic property | SlogP | [-5,9] |
| Basic property | logS | [-14,2] |
| Absorption | Caco-2 | [-8,-4] |
| Absorption | MDCK | [-8,-3] |
| Distribution | PPB | [0,1] |
| Drug-likeness score | QED | [0,1] |
| Toxicity | AMES | [0,1] |
| Toxicity | hERG | [0,1] |
| Toxicity | hepatoxicity | [0,1] |
| Toxicity | LD_50_ | [0,1] |
