# Supplementary material for: ChemMORT: an automatic ADMET optimization platform using deep learning and multi-objective particle swarm optimization
Source: Brief Bioinform. 2024 Feb 20;25(2):bbae008. doi: 10.1093/bib/bbae008 (PMC10883642; doi:10.1093/bib/bbae008)
Supplement: supplementary_materials_bbae008 [file supplementary_materials_bbae008.zip › supplementary_materials_bbae008/Table S3.docx]

**Table S3.** The information about ADMET prediction models

| **Property** | **Parameter** | | | |
| --- | --- | --- | --- | --- |
|  | **n_estimators** | **max_depth** | **learning_rate** | **subsample** |
| logD7.4 | 1200 | 6 | 0.02 | 0.4 |
| logS | 1200 | 6 | 0.02 | 1 |
| Caco-2 | 1000 | 8 | 0.02 | 0.7 |
| MDCK | 1000 | 6 | 0.05 | 0.8 |
| PPB | 800 | 6 | 0.06 | 0.8 |
| AMES | 1200 | 6 | 0.02 | 0.4 |
| hERG | 1200 | 8 | 0.1 | 0.8 |
| hepatoxicity | 1200 | 6 | 0.02 | 0.8 |
| LD50 | 1200 | 6 | 0.02 | 0.8 |
