# Supplementary material for: ChemMORT: an automatic ADMET optimization platform using deep learning and multi-objective particle swarm optimization
Source: Brief Bioinform. 2024 Feb 20;25(2):bbae008. doi: 10.1093/bib/bbae008 (PMC10883642; doi:10.1093/bib/bbae008)
Supplement: supplementary_materials_bbae008 [file supplementary_materials_bbae008.zip › supplementary_materials_bbae008/Table S4.docx]

**Table S4.** The detailed information about the 715 optimized molecules

| **SMILES** | **Final score** | **QED** | **logS** | **Active motif** | **Similarity** | **SA** | **docking score** |
| --- | --- | --- | --- | --- | --- | --- | --- |
| O=C(N1CCN(CC1)C(=O)c1cc(ccc1F)Cc1n[nH]c(=O)c2c1cccc2)C1CC1 | 0.535 | 0.683 | -3.787 | 1 | Initial molecule | 2.369 | -7.769 |
| CC(=O)N1CCN(C1)CCN1CCN(C1)C(=O)c1cc(ccc1F)Cc1nnn[nH]1 | 0.863 | 0.694 | -0.783 | 1 | 0.413 | 3.029 | -7.780 |
| CC(=O)N1CCN(CC1)CCN1CCN(C1)C(=O)c1cc(ccc1F)Cc1[nH]nnn1 | 0.863 | 0.678 | -0.800 | 1 | 0.413 | 2.817 | -6.431 |
| CC(=O)N1CCN(CC1=O)CCN1CCN(C1)C(=O)c1cc(ccc1F)Cc1[nH]ncn1 | 0.857 | 0.667 | -0.875 | 1 | 0.395 | 2.955 | -7.898 |
| CC(=O)N1CCN(CC1=O)CCN1CCN(C1)C(=O)c1cc(ccc1F)Cc1[nH]nnn1 | 0.854 | 0.624 | -0.880 | 1 | 0.405 | 3.066 | -6.773 |
| CC(=O)N1CCN(CC1=O)CCN1CCN(CC1)C(=O)c1cc(ccc1F)Cc1[nH]ncn1 | 0.853 | 0.650 | -1.003 | 1 | 0.405 | 2.768 | -7.648 |
| O=C(N1CCN(CC1)C(=O)C)CN1CCN(C1)C(=O)c1cc(ccc1F)Cc1[nH]nnn1 | 0.852 | 0.650 | -0.886 | 1 | 0.385 | 2.846 | -7.270 |
| CC(=O)N1CCN(CC1)CCN1CCN(CC1=O)C(=O)c1cc(ccc1F)Cc1[nH]nnn1 | 0.849 | 0.613 | -0.846 | 1 | 0.388 | 2.810 | -7.805 |
| O=C(N1CCN(C1)C(=O)C)N1CCN(C1)C(=O)c1cc(ccc1F)Cc1ncn[nH]1 | 0.848 | 0.786 | -1.110 | 1 | 0.400 | 2.973 | -8.257 |
| CC(=O)N1CCN(CC1)CCN1CCN(CC1=O)C(=O)c1cc(ccc1F)Cc1[nH]ncn1 | 0.848 | 0.654 | -0.954 | 1 | 0.383 | 2.702 | -8.265 |
| O=C(N1CCN(CC1)C(=O)C)CN1CCN(C1)C(=O)c1cc(ccc1F)Cc1[nH]ncn1 | 0.848 | 0.694 | -0.974 | 1 | 0.385 | 2.734 | -8.587 |
| O=CN1CCN(C1)CCN1CCN(CC1)C(=O)Cc1n[nH]c(=O)c2c1cccc2 | 0.857 | 0.650 | -0.939 | 1 | 0.403 | 2.719 | -10.019 |
| O=C1CN(CCN2CCN(CC2)C(=O)c2n[nH]c(=O)c3c2cccc3)CCN1C | 0.850 | 0.743 | -1.021 | 1 | 0.394 | 2.427 | -10.166 |
| NN1CCN(C1)CCN1CCN(CC1)C(=O)c1n[nH]c(=O)c2c1cccc2 | 0.849 | 0.674 | -1.101 | 1 | 0.420 | 2.615 | -10.534 |
| O=NN1CCN(C1)CCN1CCN(CC1)C(=O)Cc1n[nH]c(=O)c2c1cccc2 | 0.847 | 0.675 | -1.133 | 1 | 0.403 | 2.786 | -10.379 |
| O=CN1CCN(CC1)CCN1CCN(CC1)C(=O)c1n[nH]c(=O)c2c1cccc2 | 0.846 | 0.680 | -1.160 | 1 | 0.412 | 2.473 | -9.532 |
| CN1CCN(CC1)CCN1CCN(C1)C(=O)c1n[nH]c(=O)c2c1cccc2 | 0.845 | 0.809 | -1.179 | 1 | 0.406 | 2.441 | -9.972 |
| O=CN1CCN(CC1)CCN1CCN(CC1)C(=O)Cc1n[nH]c(=O)c2c1cccc2 | 0.845 | 0.636 | -1.162 | 1 | 0.400 | 2.553 | -9.084 |
| O=CN1CCN(C1)CCN1CCN(CC1)C(=O)c1n[nH]c(=O)c2c1cccc2 | 0.844 | 0.693 | -1.203 | 1 | 0.414 | 2.644 | -10.541 |
| OCCN1CCN(CC1)C(=O)C1CCN(C1)C(=O)Cc1n[nH]c(=O)c2c1cccc2 | 0.844 | 0.681 | -1.205 | 1 | 0.472 | 2.843 | -8.496 |
| O=CN1CCN(CC1)CCN1CCN(C1)C(=O)Cc1n[nH]c(=O)c2c1cccc2 | 0.843 | 0.650 | -1.220 | 1 | 0.403 | 2.749 | -9.302 |
| O=C(N1CCN(CC1)C(=O)c1cc(ccc1F)Cc1nn(c(=O)n1C)C)C(O)C | 0.862 | 0.740 | -0.821 | 1 | 0.411 | 3.019 | -8.780 |
| CC(CN1CCN(CC1)C(=O)c1cc(ccc1F)Cc1nn(c(=O)n1C)C)O | 0.859 | 0.775 | -0.898 | 1 | 0.458 | 3.000 | -9.534 |
| CC(C(=O)N1CCN(CC1)C(=O)c1cc(ccc1F)Cc1nn(C)c(=O)n(c1=O)C)O | 0.858 | 0.652 | -0.891 | 1 | 0.432 | 3.021 | -9.165 |
| CC(=O)N1CCN(CC1)C(=O)c1cc(ccc1F)Cc1nn(C)c(=O)n(c1=O)C | 0.858 | 0.689 | -0.919 | 1 | 0.464 | 2.461 | -7.488 |
| CC(=O)N1CCN(CC1)C(=O)c1cc(ccc1F)CSc1n[nH]c(=O)n1C | 0.854 | 0.778 | -0.983 | 1 | 0.431 | 2.407 | -8.923 |
| CC(=O)N1CCN(CC1)C(=O)c1cc(ccc1F)CCc1nn(c(=O)n1C)C | 0.853 | 0.756 | -0.959 | 1 | 0.394 | 2.483 | -9.014 |
| COCCN1CCN(CC1)C(=O)c1cc(ccc1F)Cc1n[nH]c(=O)n1C | 0.853 | 0.780 | -1.016 | 1 | 0.458 | 2.439 | -8.915 |
| OCC(N1CCN(CC1)C(=O)c1cc(ccc1F)Cc1nn(c(=O)n1C)C)C | 0.852 | 0.775 | -0.998 | 1 | 0.400 | 3.048 | -8.588 |
| OCC1CN(CCN1C(=O)C)C(=O)c1cc(ccc1F)Cc1n[nH]c(=O)n1C | 0.851 | 0.730 | -0.961 | 1 | 0.429 | 3.157 | -8.340 |
| CC(=O)N1CCCN(CC1)C(=O)c1cc(ccc1F)Cc1n[nH]c(=O)n1C | 0.850 | 0.847 | -1.064 | 1 | 0.478 | 2.453 | -7.549 |
| [O-]C(CN1CCN(CC1)Cc1ccc(c(c1)C(=O)N1CCN(CC1)C(=O)C)F)C | 0.882 | 0.690 | -0.361 | 1 | 0.394 | 2.931 | -7.949 |
| [O-]C(=O)CN1CCN(CC1)Cc1ccc(c(c1)C(=O)N1CCN(CC1)C(=O)C)F | 0.873 | 0.625 | -0.524 | 1 | 0.406 | 2.479 | -6.442 |
| CC(=O)N1CCN(CC1)C(=O)c1cc(ccc1F)CN1CCN(C(=O)C1)C | 0.869 | 0.766 | -0.628 | 1 | 0.394 | 2.269 | -7.861 |
| O=C(N(C)C)CN1CCN(C1)Cc1ccc(c(c1)C(=O)N1CCN(CC1)C(=O)C)F | 0.869 | 0.687 | -0.355 | 1 | 0.365 | 2.457 | -8.144 |
| [O-]C(=O)N1CCN(CC1)Cc1ccc(c(c1)C(=O)N1CCN(CC1)C(=O)C)F | 0.868 | 0.699 | -0.686 | 1 | 0.397 | 2.454 | -7.560 |
| [O-]C(=O)CN1CCN(C1=O)Cc1ccc(c(c1)C(=O)N1CCN(CC1)C(=O)C)F | 0.867 | 0.633 | -0.580 | 1 | 0.389 | 2.709 | -7.730 |
| CC(C(=O)[O-])CN1CCN(CC1)Cc1ccc(c(c1)C(=O)N1CCN(CC1)C(=O)C)F | 0.866 | 0.609 | -0.431 | 1 | 0.384 | 3.011 | -6.493 |
| [O-]C(=O)CN1CCN(C1)Cc1ccc(c(c1)C(=O)N1CCN(CC1)C(=O)C)F | 0.866 | 0.637 | -0.688 | 1 | 0.394 | 2.712 | -7.911 |
| O=C(c1cc(ccc1F)CN1CC[NH2+]CC1)N1CCN(CC1)C(=O)C | 0.866 | 0.801 | -0.537 | 1 | 0.377 | 2.989 | -7.637 |
| CC(=O)N1CCN(CC1)C(=O)c1cc(CCN2CCN(C2=O)C)ccc1F | 0.865 | 0.789 | -0.583 | 1 | 0.380 | 2.356 | -7.392 |
| CN1CCN(CC1)C(=O)C1CNCCN1C(=O)c1cc(ccc1F)CN1CCN(C(=O)C1)C | 0.873 | 0.640 | -0.192 | 1 | 0.361 | 3.100 | -7.640 |
| CN1CCN(CC1)C(=O)C1CCN(C1)C(=O)c1cc(ccc1F)CN1CCN(C(=O)C1)C | 0.870 | 0.669 | -0.658 | 1 | 0.429 | 2.888 | -8.083 |
| CN1CCN(CC1)C(=O)C1CCN(C1)C(=O)c1cc(ccc1F)CN1CCN(C1=O)C | 0.865 | 0.712 | -0.763 | 1 | 0.421 | 2.907 | -8.414 |
| CN1CCN(CC1)C(=O)N1CCN(CC1)C(=O)c1cc(ccc1F)Cc1nn(c(=O)[nH]1)C | 0.864 | 0.715 | -0.779 | 1 | 0.425 | 2.774 | -8.233 |
| CN1CCN(CC1)C(=O)N1CCN(CC1)C(=O)c1cc(ccc1F)Cc1nccc(=O)[nH]1 | 0.863 | 0.753 | -0.800 | 1 | 0.419 | 2.553 | -8.532 |
| CN1CCN(CC1)C(=O)N1CCN(CC1)C(=O)c1cc(ccc1F)CN1CCN(C(=O)C1)C | 0.861 | 0.646 | -0.648 | 1 | 0.378 | 2.492 | -7.997 |
| CN1CCN(CC1)C(=O)C(=O)N1CCN(CC1)C(=O)c1cc(ccc1F)CN1CNCC1 | 0.859 | 0.616 | -0.484 | 1 | 0.370 | 2.612 | -7.279 |
| CN1CCN(CC1)CCN1CCN(C1)C(=O)c1cc(ccc1F)Cc1[nH][nH]c(=O)n1 | 0.857 | 0.676 | -0.922 | 1 | 0.427 | 2.841 | -7.657 |
| CN1CCN(CC1)C(=O)C1NCCN(C1)C(=O)c1cc(ccc1F)CN1CCN(C1=O)C | 0.857 | 0.698 | -0.615 | 1 | 0.375 | 3.137 | -7.622 |
| CN1CCN(CC1)C(=O)N1CCN(C1)C(=O)c1cc(ccc1F)CN1CCOCC1=O | 0.857 | 0.684 | -0.188 | 1 | 0.321 | 2.697 | -8.008 |
| OCCN1CCN(CC1)C(=O)c1cc(ccc1F)CN1CCN(C1=O)CCO | 0.865 | 0.669 | -0.708 | 1 | 0.394 | 2.440 | -7.567 |
| OCCN1CCN(CC1)C(=O)c1cc(ccc1F)CN1CCN(CC1)C(=O)CO | 0.863 | 0.642 | -0.677 | 1 | 0.386 | 2.253 | -7.506 |
| OCc1ccc(c(c1)C(=O)N1CCN(CC1)CC(=O)N1CCOCC1)F | 0.861 | 0.809 | -0.673 | 1 | 0.380 | 2.142 | -6.942 |
| OCCN1CCN(CC1)C(=O)c1cc(ccc1F)CN1CCNCC1=O | 0.860 | 0.737 | -0.799 | 1 | 0.392 | 2.373 | -7.725 |
| OCCN1CCN(CC1)C(=O)c1cc(ccc1F)CN1CCN(C(=O)C1)CCO | 0.859 | 0.615 | -0.610 | 1 | 0.384 | 2.397 | -7.991 |
| OCCN1CCN(CC1)C(=O)c1cc(ccc1F)CN1CCNC(=O)C1 | 0.859 | 0.737 | -0.821 | 1 | 0.392 | 2.300 | -7.950 |
| OCCN1CCN(C1)Cc1ccc(c(c1)C(=O)N1CCN(C(=O)C1)CCO)F | 0.858 | 0.628 | -0.611 | 1 | 0.373 | 2.592 | -9.237 |
| OCCN1CCN(CC1)C(=O)c1cc(ccc1F)CN1CC[NH2+]C(=O)C1 | 0.857 | 0.663 | -0.894 | 1 | 0.397 | 3.032 | -7.950 |
| OCCN1CCN(CC1)Cc1ccc(c(c1)C(=O)N1CCN(CC1)CCO)F | 0.856 | 0.661 | -0.943 | 1 | 0.415 | 2.185 | -7.515 |
| OCCN1CCN(CC1)C(=O)c1cc(ccc1F)CN1CCN(CC1=O)CCO | 0.856 | 0.615 | -0.730 | 1 | 0.389 | 2.402 | -8.838 |
| OCCc1ccc(c(c1)C(=O)N1CCN(CCN(CC1)C(=O)CO)C(=O)CO)F | 0.851 | 0.540 | -0.574 | 1 | 0.400 | 2.793 | -6.925 |
| OCc1ccc(c(c1)C(=O)N1CCN(CCN(CC1)C(=O)CO)C(=O)CO)F | 0.848 | 0.564 | -0.745 | 1 | 0.413 | 2.749 | -8.861 |
| OCc1ccc(c(c1)C(=O)N1CCN(CC1)C(=O)CN1CCOCC1)F | 0.848 | 0.809 | -0.803 | 1 | 0.366 | 2.134 | -7.646 |
| OCc1ccc(c(c1)F)C(=O)N1CCN(CCN(CC1)C(=O)C1CC1)C(=O)CO | 0.847 | 0.722 | -1.136 | 1 | 0.448 | 2.682 | -5.825 |
| OCN1CCN(CCN(CC1)C(=O)c1cc(CO)ccc1F)C(=O)CO | 0.846 | 0.619 | -1.054 | 1 | 0.403 | 2.785 | -7.438 |
| OCC(CN1CCN(CCN(C1=O)C(=O)c1ccccc1F)C(=O)C1CC1)CO | 0.846 | 0.717 | -1.161 | 1 | 0.403 | 2.661 | -7.739 |
| OCC(=O)N1CCN(CC1)C(=O)C1CCN(C1)C(=O)c1ccccc1F | 0.844 | 0.817 | -1.110 | 1 | 0.391 | 2.533 | -7.589 |
| OCC(=O)N1CCN(CCN(C1=O)C(=O)C1CC1)C(=O)c1ccccc1F | 0.843 | 0.825 | -1.208 | 1 | 0.420 | 2.475 | -5.820 |
| OCC(N1CCN(CCN1C(=O)C1CC1)C(=O)c1ccccc1F)CO | 0.843 | 0.773 | -1.210 | 1 | 0.400 | 2.708 | -5.812 |
| OCC(=O)N1CCN(CCN(C1)C(=O)CO)C(=O)c1cc(CF)ccc1F | 0.843 | 0.717 | -1.161 | 1 | 0.394 | 2.659 | -6.958 |
| OCCN1CCN(CC1)Cc1ccc(c(c1)C(=O)N1CCN(CC1)CC=O)F | 0.861 | 0.654 | -0.789 | 1 | 0.394 | 2.325 | -7.007 |
| OCCN1CCN(CC1)Cc1ccc(c(c1)C(=O)N1CCN(CC1)C(=O)CO)F | 0.860 | 0.642 | -0.733 | 1 | 0.386 | 2.253 | -7.614 |
| OCCN1CCN(CC1)Cc1ccc(c(c1)C(=O)N1CCN(CC1)C=O)F | 0.860 | 0.698 | -0.815 | 1 | 0.394 | 2.364 | -7.696 |
| OCC(=O)N1CCN(CC1)Cc1ccc(c(c1)C(=O)N1CCNCC1)F | 0.860 | 0.754 | -0.879 | 1 | 0.400 | 2.264 | -6.692 |
| OCCN1CCN(CC1)C(=O)Cc1ccc(c(c1)C(=O)N1CCN(CC1)CCO)F | 0.859 | 0.602 | -0.700 | 1 | 0.403 | 2.271 | -7.907 |
| OCN1CCN(C1)C(=O)c1cc(ccc1F)CN1CCN(CC1=O)C(=O)C1CC1 | 0.840 | 0.751 | -1.279 | 1 | 0.466 | 2.666 | -7.875 |
| OCCN1CCN(CC1)C(=O)c1cc(ccc1F)CN1CCN(CC1=O)C(=O)C1CC1 | 0.837 | 0.692 | -1.342 | 1 | 0.486 | 2.403 | -6.836 |
| O=C(c1cc(ccc1F)Cc1n[nH]ccc1=O)N1CCN(CC1)C(=O)CN1CCCC1 | 0.835 | 0.765 | -1.377 | 1 | 0.493 | 2.654 | -7.935 |
| O=C(c1cc(ccc1F)Cc1n[nH]ccc1=O)N1CCN(CC1)C(=O)C(=O)N1CCOCC1 | 0.833 | 0.630 | -1.380 | 1 | 0.479 | 2.852 | -8.198 |
| O=C(N1CCN(C1)C(=O)c1cc(ccc1F)Cc1n[nH]ccc1=O)CN1CCCC1 | 0.830 | 0.785 | -1.474 | 1 | 0.459 | 2.849 | -9.088 |
| O=C1CCN1CCN1CCN(CC1)C(=O)c1cc(ccc1F)Cc1n[nH]ccc1=O | 0.830 | 0.697 | -1.488 | 1 | 0.479 | 2.788 | -7.418 |
| O=C1CN1CCN1CCN(CC1)C(=O)c1cc(ccc1F)Cc1n[nH]c(=O)c(c1)F | 0.829 | 0.681 | -1.438 | 1 | 0.500 | 3.133 | -8.058 |
| OCCn1c(n[nH]c1=O)Cc1ccc(c(c1)C(=O)N1CCN(C1)C(=O)C1CCCO1)F | 0.824 | 0.646 | -1.422 | 1 | 0.427 | 3.324 | -8.562 |
| OCc1cccnc1Cc1ccc(c(c1)C(=O)N1CCN(CC1)C(=O)C(=O)[O-])F | 0.824 | 0.682 | -1.605 | 1 | 0.458 | 2.740 | -7.573 |
| O=C(N1CCN(CC1)C(=O)c1cc(ccc1F)Cc1n[nH]c(=O)c(=O)[nH]1)C1CC1 | 0.823 | 0.693 | -1.621 | 1 | 0.694 | 2.609 | -7.214 |
| OCN(N1CCN(CC1)C(=O)c1cc(ccc1F)Cc1n[nH]c(=O)n1C)C | 0.873 | 0.674 | -0.597 | 1 | 0.452 | 2.946 | -8.603 |
| CCOCN(N1CCN(CC1)C(=O)c1cc(ccc1F)Cc1n[nH]c(=O)n1C)C | 0.869 | 0.672 | -0.687 | 1 | 0.429 | 2.978 | -6.928 |
| OCN(N1CCN(C1)C(=O)c1cc(ccc1F)Cc1n[nH]c(=O)n1C)C | 0.863 | 0.685 | -0.715 | 1 | 0.421 | 3.153 | -7.509 |
| [O-]C(=O)N1CCN(CC1)C(=O)c1cc(ccc1F)Cc1n[nH]c(=O)n1C | 0.861 | 0.747 | -0.841 | 1 | 0.485 | 2.792 | -8.062 |
| CC(N1CCN(CC1)C(=O)c1cc(ccc1F)Cc1nn(c(=O)n1C)C)O | 0.860 | 0.808 | -0.830 | 1 | 0.411 | 3.073 | -8.498 |
| CC(N1CCN(CC1)C(=O)c1cc(ccc1F)Cc1n[nH]c(=O)n1C)O | 0.857 | 0.793 | -0.914 | 1 | 0.465 | 3.040 | -7.984 |
| [O-]C(=O)CN1CCN(CC1)C(=O)c1cc(ccc1F)Cc1n[nH]c(=O)n1C | 0.853 | 0.669 | -1.004 | 1 | 0.486 | 2.800 | -6.899 |
| OCC(N1CCN(CC1)C(=O)c1cc(ccc1F)Cc1n[nH]c(=O)n(c1=O)C)C | 0.852 | 0.683 | -0.989 | 1 | 0.493 | 3.084 | -7.366 |
| CC(N1CCN(CC1)C(=O)c1cc(ccc1F)Cc1nn(C)c(=O)n(c1=O)C)O | 0.859 | 0.722 | -0.846 | 1 | 0.432 | 3.063 | -7.698 |
| COCC(=O)N1CCN(CC1)C(=O)c1cc(ccc1F)Cc1n[nH]c(=O)n1C | 0.854 | 0.759 | -0.984 | 1 | 0.465 | 2.481 | -7.860 |
| COCN1CCN(CC1)C(=O)c1cc(ccc1F)Cc1n[nH]c(=O)n1C | 0.852 | 0.821 | -1.041 | 1 | 0.465 | 2.592 | -7.039 |
| ON1CCN(CC1)C(=O)c1cc(ccc1F)CN1CCNCC1=O | 0.865 | 0.807 | -0.716 | 1 | 0.394 | 2.609 | -7.226 |
| OCCN1CCN(CC1)C(=O)c1cc(ccc1F)CN1CC1O | 0.856 | 0.729 | -0.942 | 1 | 0.400 | 2.997 | -7.834 |
| OCN1CCN(CC1)C(=O)c1cc(ccc1F)CN1CCNCC1=O | 0.856 | 0.763 | -0.959 | 1 | 0.403 | 2.484 | -7.964 |
| OCCN1CCN(CC1)C(=O)c1cc(ccc1F)CN1CC(CC1=O)O | 0.855 | 0.747 | -0.842 | 1 | 0.387 | 2.803 | -8.325 |
| O=C1CNCCN1Cc1ccc(c(c1)C(=O)N1CCN(CC1)CC(=O)N1CC1)F | 0.852 | 0.662 | -0.950 | 1 | 0.392 | 2.377 | -7.575 |
| O=CN1CCN(CC1)C(=O)c1cc(ccc1F)CN1CCNCC1 | 0.852 | 0.796 | -1.030 | 1 | 0.400 | 2.400 | -7.477 |
| OCCN1CCN(CC1)C(=O)c1ccc(c(c1)C(=O)N1CCN(CC1)CCO)F | 0.852 | 0.649 | -0.692 | 1 | 0.364 | 2.195 | -8.121 |
| CN1CCN(CC1)CC(=O)N1CCN(CC1)C(=O)Cc1n[nH]c(=O)c2c1cccc2 | 0.852 | 0.720 | -1.029 | 1 | 0.400 | 2.331 | -10.152 |
| CN1CCN(CC1)CC(=O)N1CCN(C1)C(=O)Cc1n[nH]c(=O)c2c1cccc2 | 0.850 | 0.735 | -0.968 | 1 | 0.389 | 2.518 | -9.422 |
| O=C(N1CCN(CC1)C(=O)c1n[nH]c(=O)c2c1cccc2)CN1CC[NH2+]CC1 | 0.849 | 0.641 | -1.005 | 1 | 0.394 | 3.051 | -9.387 |
| OC(N1CCN(CC1)C(=O)c1n[nH]c(=O)c2c1cccc2)CN1CCOCC1 | 0.849 | 0.718 | -1.023 | 1 | 0.392 | 2.955 | -10.317 |
| CN1CCN(CC1)C(=O)CN1CCN(CC1)C(=O)c1n[nH]c(=O)c2c1cccc2 | 0.845 | 0.746 | -1.174 | 1 | 0.400 | 2.242 | -9.117 |
| CN1CCN(CC1)C(=O)N(N1CCN(CC1)C(=O)Cc1n[nH]c(=O)c2c1cccc2)C | 0.845 | 0.730 | -0.925 | 1 | 0.373 | 2.765 | -7.364 |
| O=C(N1CCN(CC1)CCc1n[nH]c(=O)c2c1cccc2)CN1COCC1 | 0.845 | 0.791 | -1.105 | 1 | 0.392 | 2.559 | -9.921 |
| O=C(N1CCN(CC1)C(=O)c1n[nH]c(=O)c2c1cccc2)CN1CCNCC1 | 0.845 | 0.712 | -1.182 | 1 | 0.400 | 2.318 | -9.387 |
| O=C(N1CCN(CC1)C(=O)c1n[nH]c(=O)c2c1cccc2)CCN1CCOCC1 | 0.844 | 0.773 | -1.191 | 1 | 0.403 | 2.276 | -10.251 |
| CN1CCN(CC1)C(=O)CN1CCN(CC1)C(=O)Cc1n[nH]c(=O)c2c1cccc2 | 0.844 | 0.720 | -1.196 | 1 | 0.400 | 2.331 | -6.119 |
| CC(=O)N1CCN(CC1)C(=O)c1cc(ccc1F)CCc1n[nH]n(c1=O)C | 0.862 | 0.834 | -0.829 | 1 | 0.443 | 2.692 | -8.000 |
| CC(=O)N1CCN(CC1)C(=O)c1cc(ccc1F)CCc1n[nH]c(=O)n(c1=O)C | 0.861 | 0.754 | -0.838 | 1 | 0.471 | 2.512 | -8.974 |
| CC(=O)N1CCN(CC1)C(=O)c1cc(ccc1F)CCc1nn(C)c(=O)n(c1=O)C | 0.860 | 0.681 | -0.853 | 1 | 0.397 | 2.494 | -9.386 |
| CC(=O)N1CCN(CC1)C(=O)c1cc(ccc1F)CCc1n[nH]c(=O)n1C | 0.856 | 0.834 | -0.951 | 1 | 0.449 | 2.429 | -8.877 |
| Fc1ccc(cc1C(=O)N1CCN(CC1)S(=O)(=O)C)CCc1n[nH]c(=O)n1C | 0.853 | 0.730 | -1.007 | 1 | 0.419 | 2.519 | -8.853 |
| CC(=O)N1CCN(CC1)C(=O)c1cc(ccc1F)CCc1n[nH]c(=O)c(=O)n1C | 0.848 | 0.705 | -1.116 | 1 | 0.486 | 2.497 | -8.220 |
| NC(=O)N1CCN(CC1)C(=O)c1cc(ccc1F)Cc1n[nH]c(=O)c(=O)n1C | 0.848 | 0.651 | -1.116 | 1 | 0.522 | 2.560 | -8.080 |
| CC(=O)N1CCN(CC1)C(=O)c1cc(ccc1F)CCc1n[nH]n(c(=O)c1=O)C | 0.848 | 0.705 | -1.118 | 1 | 0.431 | 2.785 | -7.338 |
| OCC(=O)N1CCN(CC1)C(=O)c1cc(ccc1F)Cc1n[nH]c(=O)n1C | 0.865 | 0.718 | -0.768 | 1 | 0.478 | 2.518 | -8.106 |
| OCCC(=O)N1CCN(CC1)C(=O)c1cc(ccc1F)Cc1n[nH]c(=O)n1C | 0.859 | 0.714 | -0.886 | 1 | 0.465 | 2.535 | -7.830 |
| O=C1CCN(CN1C)C(=O)c1cc(ccc1F)Cc1n[nH]c(=O)n1C | 0.859 | 0.852 | -0.895 | 1 | 0.431 | 2.794 | -7.791 |
| O=C(N1CCC(CC1)C(=O)[O-])Cc1ccc(c(c1)C(=O)N1CCN(CC1)C(=O)C)F | 0.853 | 0.666 | -1.012 | 1 | 0.414 | 2.536 | -8.698 |
| [O-]C(=O)N1CCN(CC1)C(=O)c1cc(ccc1F)Cc1c[nH]c(=O)n1C | 0.853 | 0.800 | -1.022 | 1 | 0.423 | 2.931 | -6.991 |
| OCC(=O)N(N1CCN(C1)C(=O)c1cc(ccc1F)Cc1n[nH]c(=O)n1C)C | 0.852 | 0.669 | -0.972 | 1 | 0.410 | 3.093 | -7.548 |
| CC(=O)N1CCN(CC1)C(=O)c1cc(ccc1F)CN1CCN(C1)C(=O)C | 0.875 | 0.781 | -0.556 | 1 | 0.409 | 2.331 | -8.078 |
| OCCN1CCN(C1)Cc1ccc(c(c1)C(=O)N1CCN(CC1)C(=O)C)F | 0.873 | 0.793 | -0.609 | 1 | 0.400 | 2.345 | -8.524 |
| CC(=O)N1CCN(C1)Cc1ccc(c(c1)C(=O)N1CCN(C1)C(=O)C)F | 0.871 | 0.794 | -0.477 | 1 | 0.382 | 2.577 | -8.475 |
| CC(=O)N1CCN(CC1)C(=O)c1cc(CCN2CCN(C2)C(=O)C)ccc1F | 0.868 | 0.763 | -0.700 | 1 | 0.418 | 2.387 | -8.814 |
| CC(=O)N1CCN(CC1)Cc1ccc(c(c1)C(=O)N1CCN(CC1)C(=O)C)F | 0.864 | 0.766 | -0.777 | 1 | 0.429 | 2.111 | -8.212 |
| CN1CCN(C1)CCc1ccc(c(c1)C(=O)N1CCN(C(=O)C1)C(=O)C)F | 0.862 | 0.761 | -0.647 | 1 | 0.382 | 2.614 | -8.563 |
| CN1CCN(CC1)Cc1ccc(c(c1)C(=O)N1CCN(CC1)C(=O)C)F | 0.862 | 0.801 | -0.821 | 1 | 0.403 | 2.062 | -7.296 |
| CN1CCN(CC1)Cc1ccc(c(c1)C(=O)N1CCN(C(=O)C1)C(=O)C)F | 0.861 | 0.764 | -0.641 | 1 | 0.378 | 2.363 | -8.674 |
| OCc1ccc(c(c1)C(=O)N1CCN(CC1)CC(=O)N1CCC(C1)O)F | 0.868 | 0.763 | -0.710 | 1 | 0.403 | 2.651 | -8.477 |
| O=C(N1CCN(CC1)C(=O)c1cc(ccc1F)CN1CCN(CC1)C(=O)C)C(=O)C | 0.865 | 0.660 | -0.740 | 1 | 0.397 | 2.305 | -7.818 |
| CC(=O)N1CCN(CC1)Cc1ccc(c(c1)C(=O)N1CCN(C1)C(=O)C)F | 0.860 | 0.781 | -0.747 | 1 | 0.388 | 2.336 | -8.264 |
| CN1CCN(CC1)C(=O)c1cc(ccc1F)Cc1nn(C)c(=O)c(=O)n1C | 0.859 | 0.682 | -0.882 | 1 | 0.423 | 2.509 | -6.687 |
| OCc1ccc(c(c1)C(=O)N1CCN(CC1)C(=O)C(=O)N1CCN(CC1)C)F | 0.857 | 0.678 | -0.870 | 1 | 0.394 | 2.310 | -6.366 |
| CC(=O)N1CCN(CC1)C(=O)c1cc(ccc1F)CN1CCN(C1)C(=O)[O-] | 0.857 | 0.712 | -0.795 | 1 | 0.386 | 2.614 | -8.239 |
| OCCN1CCN(CC1)C(=O)c1cc(ccc1F)CC1CN(C1=O)CCO | 0.859 | 0.628 | -0.835 | 1 | 0.403 | 2.937 | -9.058 |
| CN1CCN1Cc1ccc(c(c1)C(=O)N1CCN(CC1)C(=O)C)F | 0.874 | 0.820 | -0.462 | 1 | 0.388 | 2.512 | -7.067 |
| CC(=O)N1CCN(CC1)C(=O)c1cc(ccc1F)CN1CCC1=O | 0.871 | 0.771 | -0.653 | 1 | 0.403 | 2.137 | -7.169 |
| CC(=O)N1CCN(CC1)C(=O)c1cc(ccc1F)CN1CCN2C(C1)C2=O | 0.869 | 0.707 | -0.693 | 1 | 0.408 | 2.973 | -8.298 |
| O=C(N1CCN(CC1)Cc1ccc(c(c1)C(=O)N1CCN(CC1)C(=O)C)F)C1CO1 | 0.877 | 0.652 | -0.527 | 1 | 0.429 | 2.749 | -7.779 |
| O=C(CN1CCN(CC1)C(=O)C)NCc1ccc(c(c1)C(=O)N1CCN(CC1)C(=O)C)F | 0.875 | 0.681 | -0.558 | 1 | 0.426 | 2.225 | -8.216 |
| O=C(N1CCN(CC1)Cc1ccc(c(c1)C(=O)N1CCN(CC1)C(=O)C)F)C1OCCO1 | 0.869 | 0.652 | -0.693 | 1 | 0.423 | 2.619 | -7.713 |
| CC(=O)N1CCN(CC1)C(=O)c1cc(ccc1F)CN1CCN2C(C1)OCC2=O | 0.865 | 0.716 | -0.620 | 1 | 0.387 | 3.053 | -7.336 |
| CC(=O)N1CCN(CC1)C(=O)c1cc(ccc1F)CN1CCN(CC1)S(=O)(=O)C | 0.865 | 0.688 | -0.590 | 1 | 0.380 | 2.208 | -7.846 |
| CC(=O)N1CCN(CC1)C(=O)c1cc(ccc1F)CN1CCN2C1CCC2=O | 0.864 | 0.772 | -0.761 | 1 | 0.397 | 2.979 | -8.366 |
| O=C(N1CCN(CO1)Cc1ccc(c(c1)C(=O)N1CCN(CC1)C(=O)C)F)C1CC1 | 0.864 | 0.732 | -0.793 | 1 | 0.458 | 2.718 | -7.423 |
| CC(=O)N1CCN(CC1)C(=O)c1cc(ccc1F)CN1CCN(CC1)S(=O)C | 0.861 | 0.727 | -0.847 | 1 | 0.400 | 2.984 | -7.983 |
| CCC(=O)N1CCN(CC1)C(=O)c1cc(ccc1F)CN1CCN(CC1)C(=O)[O-] | 0.861 | 0.703 | -0.850 | 1 | 0.400 | 2.491 | -7.766 |
| OCc1ccc(cc1C(=O)N1CCN(CC1)C)Cc1n[nH]c(=O)c(=O)n1C | 0.860 | 0.661 | -0.860 | 1 | 0.444 | 2.602 | -7.749 |
| COCCN1CCN(CC1)C(=O)c1cc(ccc1F)Cc1n[nH]c(=O)c(=O)n1C | 0.855 | 0.657 | -0.980 | 1 | 0.493 | 2.506 | -7.184 |
| OCCc1ccc(cc1C(=O)N1CCN(CC1)C)Cc1n[nH]c(=O)c(=O)n1C | 0.854 | 0.636 | -0.967 | 1 | 0.400 | 2.630 | -8.147 |
| COCCN1CCN(CC1)C(=O)c1cc(ccc1F)Cc1n[nH]c(=O)n(c1=O)C | 0.854 | 0.702 | -0.991 | 1 | 0.500 | 2.519 | -7.252 |
| CN1CCN(CC1)C(=O)c1cc(ccc1C(=O)N(C)C)Cc1n[nH]c(=O)c(=O)n1C | 0.854 | 0.653 | -0.948 | 1 | 0.395 | 2.615 | -7.065 |
| COc1ccc(cc1C(=O)N1CCN(CC1)CCOC)Cc1n[nH]c(=O)n(c1=O)C | 0.853 | 0.645 | -0.862 | 1 | 0.385 | 2.494 | -6.724 |
| COCN1CCN(CC1)C(=O)c1cc(ccc1F)Cc1n[nH]c(=O)n(c1=O)C | 0.852 | 0.742 | -1.025 | 1 | 0.507 | 2.661 | -8.299 |
| OCc1ccc(cc1C(=O)N1CCN(CC1)C(=O)C)Cc1n[nH]c(=O)c(=O)n1CC | 0.852 | 0.624 | -0.961 | 1 | 0.432 | 2.638 | -7.523 |
| O=C(N1CCN(CC1)C(=O)c1n[nH]c(=O)c(=O)n1c1ccccn1)C1CC1 | 0.842 | 0.693 | -1.243 | 1 | 0.449 | 2.622 | -5.636 |
| CCn1c(n[nH]c(=O)c1=O)C(=O)N1CCN(CC1)C(=O)C1CC1 | 0.841 | 0.692 | -1.257 | 1 | 0.424 | 2.561 | -7.368 |
| O=C(N1CCN(CC1)C(=O)C1CC1)Cn1[nH]c(=O)c2c(c1=O)nccc2 | 0.840 | 0.771 | -1.280 | 1 | 0.403 | 2.494 | -6.105 |
| O=C(N1CCN(CC1)C(=O)c1n[nH]c(=O)c(=O)n1Cc1ccccn1)C1CC1 | 0.839 | 0.680 | -1.294 | 1 | 0.444 | 2.591 | -7.376 |
| O=C(N1CCN(CC1)C(=O)C1CC1)Cn1[nH]c(=O)c2c1nccc2 | 0.839 | 0.845 | -1.256 | 1 | 0.394 | 2.495 | -7.332 |
| O=C(C1CCN(C1)C(=O)C1CC1)N1CCN(CC1)C(=O)c1ccc[nH]c1=O | 0.838 | 0.803 | -1.283 | 1 | 0.397 | 2.736 | -7.710 |
| OCC(=O)N1CCN(CC1)C(=O)c1n[nH]c(=O)c2c1cccc2 | 0.838 | 0.754 | -1.323 | 1 | 0.438 | 2.168 | -10.172 |
| O=C(N1CCN(CC1)C(=O)C1CC1)CCn1[nH]c(=O)c2c(c1=O)nccc2 | 0.838 | 0.784 | -1.252 | 1 | 0.392 | 2.521 | -6.635 |
| O=C(N1CCN(CC1)C(=O)C1CN1)Cc1n[nH]c(=O)c2c1cccc2 | 0.838 | 0.704 | -1.330 | 1 | 0.492 | 2.803 | -10.011 |
| O=C(N1CCN(CC1)C(=O)c1n[nH]c(=O)c2n1ccc2)C1CC1 | 0.835 | 0.825 | -1.372 | 1 | 0.433 | 2.562 | -7.882 |
| OCC(=O)N1CCN(CC1)C(=O)c1cc(ccc1F)Cc1n[nH]c(=O)n(c1=O)C | 0.860 | 0.636 | -0.851 | 1 | 0.522 | 2.593 | -8.373 |
| COCC(=O)N1CCN(CC1)C(=O)c1cc(ccc1F)Cc1n[nH]c(=O)n(c1=O)C | 0.859 | 0.673 | -0.880 | 1 | 0.507 | 2.560 | -6.804 |
| CC(=O)N1CCN(CC1)C(=O)c1cc(ccc1F)Cc1n[nH]c(=O)c(=O)n1C | 0.858 | 0.712 | -0.916 | 1 | 0.537 | 2.487 | -8.376 |
| CC(=O)N1CCN(CC1)C(=O)c1cc(ccc1F)Cc1nccn(c1=O)C | 0.856 | 0.799 | -0.943 | 1 | 0.457 | 2.439 | -8.327 |
| COCC(=O)N1CCN(CC1)C(=O)c1cc(CCN2CCC(=O)C2)ccc1F | 0.855 | 0.709 | -0.619 | 1 | 0.364 | 2.376 | -8.080 |
| COCN1CCN(CC1)C(=O)c1cc(ccc1F)Cc1n[nH]c(=O)c(=O)n1C | 0.853 | 0.694 | -1.007 | 1 | 0.500 | 2.647 | -6.129 |
| O=C(N1CCN(CC1)C(=O)C)Cc1ccc(c(c1)C(=O)N1CCN(CC1)C(=O)C)F | 0.860 | 0.710 | -0.876 | 1 | 0.406 | 2.202 | -7.654 |
| OCCN1CCN(CC1)C(=O)c1cc(ccc1F)CC(=O)N1CCN(CC1)C(=O)C | 0.858 | 0.714 | -0.731 | 1 | 0.380 | 2.237 | -7.083 |
| [O-]C(=O)N1CCN(CC1)CCc1ccc(c(c1)C(=O)N1CCN(CC1)C(=O)C)F | 0.855 | 0.682 | -0.833 | 1 | 0.386 | 2.503 | -8.663 |
| OCCN1CCN(CC1)C(=O)Cc1ccc(c(c1)C(=O)N1CCN(CC1)C(=O)C)F | 0.854 | 0.714 | -0.815 | 1 | 0.380 | 2.237 | -7.959 |
| O=CN1CCN(CC1)C(=O)Cc1ccc(c(c1)C(=O)N1CCN(CC1)C(=O)C)F | 0.854 | 0.661 | -0.866 | 1 | 0.386 | 2.405 | -7.945 |
| O=C(N1CCN(CC1)C(=O)c1cc(ccc1F)CC(=O)N1CCNC1=O)C1CC1 | 0.853 | 0.799 | -1.016 | 1 | 0.561 | 2.384 | -7.915 |
| O=C(N1CCN(CC1)C(=O)c1cc(CCN2CCNC(=O)C2=O)ccc1F)C1CC1 | 0.849 | 0.686 | -1.088 | 1 | 0.536 | 2.499 | -8.188 |
| O=C(N1CCN(CC1)C(=O)[O-])Cc1ccc(c(c1)C(=O)N1CCN(CC1)C(C)C)F | 0.848 | 0.687 | -0.691 | 1 | 0.356 | 2.565 | -7.282 |
| CC(=O)N1CCN(CC1)C(=O)c1cc(CCN2CCN3C2CCC3=O)ccc1F | 0.848 | 0.752 | -1.019 | 1 | 0.392 | 3.023 | -9.218 |
| OCCN1CCN(CC1)C(=O)c1n[nH]c(=O)c2c(c1=O)cccc2 | 0.843 | 0.750 | -1.189 | 1 | 0.397 | 2.267 | -9.491 |
| OCc1[nH]nc(c(=O)c1)C(=O)N1CCN(CC1)C(=O)C1CC1 | 0.843 | 0.741 | -1.221 | 1 | 0.403 | 2.480 | -8.202 |
| [C]N1CCN(CC1)C(=O)c1n[nH]c(=O)c2c(c1=O)cccc2 | 0.842 | 0.773 | -1.229 | 1 | 0.415 | 2.864 | -9.151 |
| NCCN1CCN(CC1)C(=O)c1n[nH]c(=O)c2c(c1=O)cccc2 | 0.842 | 0.747 | -1.239 | 1 | 0.412 | 2.314 | -7.416 |
| OCCN1CCN(CC1)C(=O)c1n[nH]c(=O)c2c1cccc2 | 0.840 | 0.810 | -1.273 | 1 | 0.431 | 2.094 | -10.635 |
| OCCN1CCN(CC1)C(=O)Cc1n[nH]c(=O)c2c1cccc2 | 0.840 | 0.801 | -1.276 | 1 | 0.418 | 2.196 | -10.022 |
| OCN1CCN(CC1)C(=O)c1n[nH]c(=O)c2c(c1=O)cccc2 | 0.840 | 0.735 | -1.288 | 1 | 0.409 | 2.389 | -9.988 |
| O=CN1CCN(CC1)C(=O)c1n[nH]c(=O)c2c1cccc2 | 0.839 | 0.777 | -1.300 | 1 | 0.422 | 2.339 | -10.073 |
| OCCN1CCN(CC1)C(=O)C1CCN(C1)C(=O)c1n[nH]c(=O)c2c1cccc2 | 0.839 | 0.719 | -1.307 | 1 | 0.465 | 2.756 | -11.283 |
| OCCN1CCN(CC1)C(=O)c1cc(ccc1F)CC(=O)N1CC1 | 0.867 | 0.767 | -0.720 | 1 | 0.403 | 2.044 | -7.080 |
| OCCN1CCN(CC1)CC(=O)N1CCN(CC1)C(=O)c1cc(CO)ccc1F | 0.862 | 0.642 | -0.695 | 1 | 0.386 | 2.226 | -7.861 |
| OCCN1CCN(CC1)C(=O)c1cc(ccc1F)CC(CO)O | 0.861 | 0.655 | -0.714 | 1 | 0.386 | 2.625 | -7.635 |
| OCCN1CCN(CC1)C(=O)c1cc(ccc1F)CC(=O)[O-] | 0.858 | 0.744 | -0.900 | 1 | 0.403 | 2.461 | -7.103 |
| OCCN1CCN(CC1)C(=O)Cc1ccc(c(c1)C(=O)N1CC1)F | 0.858 | 0.767 | -0.904 | 1 | 0.403 | 2.044 | -7.506 |
| OCCN1CCN(CC1)C(=O)c1cc(ccc1F)CC(=O)N1CCN(C1)CCO | 0.857 | 0.615 | -0.816 | 1 | 0.406 | 2.462 | -8.767 |
| OCC(=O)N1CCN(CC1)C(=O)c1cc(ccc1F)CN1CCNCC1 | 0.856 | 0.754 | -0.813 | 1 | 0.386 | 2.260 | -7.065 |
| OCCN1CCN(CC1)C(=O)c1cc(ccc1F)CC1CCN(C1=O)CCO | 0.854 | 0.679 | -0.989 | 1 | 0.417 | 2.936 | -7.318 |
| CC(=O)N1CCN(CC1)C(=O)c1cc(ccc1F)CCn1[nH]c(=O)c(=O)n(c1=O)C | 0.866 | 0.633 | -0.684 | 1 | 0.397 | 2.644 | -8.916 |
| CC(=O)N1CCN(CC1)C(=O)c1cc(CCN2NC(=O)C(=O)C(=O)N2C)ccc1F | 0.856 | 0.633 | -0.897 | 1 | 0.397 | 2.933 | -8.228 |
| OCn1c(=O)[nH]nc(c1=O)CCc1ccc(c(c1)C(=O)N1CCN(C1)C(=O)C)F | 0.853 | 0.661 | -1.021 | 1 | 0.416 | 2.891 | -7.733 |
| OCCn1c(=O)nc([nH]c1=O)Cc1ccc(c(c1)C(=O)N1CCN(CC1)C(=O)C)F | 0.852 | 0.637 | -1.012 | 1 | 0.408 | 2.649 | -8.456 |
| CC(=O)N1CCN(CC1)C(=O)c1cc(ccc1F)CCn1c(=O)[nH]c(=O)c(=O)n1C | 0.852 | 0.633 | -0.920 | 1 | 0.392 | 2.699 | -8.491 |
| CC(=O)N1CCN(CC1)C(=O)c1cc(ccc1F)CCc1n[nH]c(=O)c(=O)n(c1=O)C | 0.852 | 0.623 | -0.956 | 1 | 0.522 | 2.605 | -8.826 |
| OCC(=O)N1CCN(CC1=O)C(=O)c1cc(ccc1F)Cc1n[nH]c(=O)n1C | 0.862 | 0.663 | -0.823 | 1 | 0.421 | 2.793 | -7.899 |
| O=CCN1CCN(CC1)C(=O)c1cc(ccc1F)Cc1n[nH]c(=O)n1C | 0.847 | 0.745 | -1.133 | 1 | 0.471 | 2.609 | -8.297 |
| NCC(=O)N1CCN(C1)C(=O)c1cc(ccc1F)Cc1n[nH]c(=O)n1C | 0.847 | 0.726 | -1.143 | 1 | 0.438 | 2.768 | -8.785 |
| OCC(N1CCN(CC1)C(=O)c1cc(ccc1F)Cc1n[nH]c(=O)n1C)C | 0.846 | 0.764 | -1.149 | 1 | 0.452 | 3.013 | -8.661 |
| OCN(C(=O)N1CCN(C1)C(=O)c1cc(ccc1F)Cc1n[nH]c(=O)n1C)C | 0.845 | 0.685 | -1.166 | 1 | 0.421 | 3.010 | -8.364 |
| OCc1ccc(cc1C(=O)N1CCN(CC1)C(=O)CO)Cc1n[nH]c(=O)n1C | 0.845 | 0.568 | -0.806 | 1 | 0.397 | 2.650 | -8.162 |
| OCC1CN(CCN1C(=O)NC)C(=O)c1cc(ccc1F)Cc1n[nH]c(=O)n1C | 0.844 | 0.619 | -0.961 | 1 | 0.418 | 3.237 | -7.575 |
| OCC(=O)N1CCN(CC1)C(=O)c1cc(ccc1F)CN1CCNC1=O | 0.850 | 0.762 | -1.079 | 1 | 0.400 | 2.411 | -8.259 |
| OCC(=O)N1CCN(CC1)C(=O)c1cc(ccc1F)CN1CCCNC1=O | 0.847 | 0.772 | -0.986 | 1 | 0.384 | 2.433 | -8.186 |
| OCCN1CCN(CC1)C(=O)c1cc(ccc1F)Cc1nn(CCO)ccc1=O | 0.846 | 0.658 | -1.166 | 1 | 0.458 | 2.551 | -8.657 |
| OCCN1CCN(CC1)C(=O)c1cc(ccc1F)Cc1nccn(c1=O)CCO | 0.845 | 0.658 | -1.186 | 1 | 0.452 | 2.582 | -7.792 |
| OCCn1ccc(=O)c(n1)Cc1ccc(c(c1)C(=O)N1CCN(CC1)C(=O)C)F | 0.843 | 0.775 | -1.219 | 1 | 0.438 | 2.519 | -7.964 |
| OCCN1CCN(CC1)C(=O)c1cc(ccc1F)Cc1ccc(=O)n(n1)CCO | 0.842 | 0.658 | -1.246 | 1 | 0.425 | 2.467 | -8.072 |
| OCCN1CCN(C(=O)C1)Cc1ccc(c(c1)C(=O)N1CCOCC1)F | 0.841 | 0.791 | -0.785 | 1 | 0.351 | 2.337 | -7.569 |
| OCCN1CCN(C1)C(=O)c1cc(ccc1F)Cc1nn(CCO)c(=O)cc1F | 0.840 | 0.659 | -1.286 | 1 | 0.452 | 2.866 | -8.538 |
| OCCn1ccc(=O)c(n1)Cc1ccc(c(c1)C(=O)N1CCN(C(=O)C1)CCF)F | 0.839 | 0.692 | -1.297 | 1 | 0.400 | 2.854 | -7.201 |
| CN1CCN(CC1)C(=O)c1cc(ccc1F)Cc1nc(=O)n(c(=O)n1C)C | 0.873 | 0.729 | -0.595 | 1 | 0.429 | 2.556 | -7.356 |
| CN1CCN(CC1)C(=O)N1CCN(C1)C(=O)c1cc(ccc1F)Cc1nn(c(=O)n1C)C | 0.868 | 0.660 | -0.586 | 1 | 0.387 | 2.834 | -8.193 |
| CN1CCN(CC1)C(=O)N1CCN(CC1)C(=O)c1cc(ccc1F)CN1CN(C(=O)C1)C | 0.867 | 0.663 | -0.399 | 1 | 0.365 | 2.595 | -8.351 |
| CN1CCN(CC1)C(=O)C1CN(C1)C(=O)c1cc(ccc1F)Cc1nn(c(=O)n1C)C | 0.865 | 0.665 | -0.769 | 1 | 0.472 | 2.655 | -8.251 |
| CN1CCN(CC1)C(=O)N1CCN(CC1)C(=O)c1cc(ccc1F)Cc1nn(c(=O)n1C)C | 0.862 | 0.644 | -0.822 | 1 | 0.423 | 2.645 | -8.690 |
| CN1CCN(C1)C(=O)N1CCN(C1)C(=O)c1cc(ccc1F)Cc1nn(C)c(=O)n(c1=O)C | 0.862 | 0.598 | -0.615 | 1 | 0.408 | 3.032 | -8.053 |
| CN1CCN(CC1)C(=O)N1CCN(CC1)C(=O)c1cc(ccc1F)Cc1nc(=O)n(n1C)C | 0.859 | 0.644 | -0.880 | 1 | 0.417 | 2.834 | -8.864 |
| O=C(C1CCS(=O)(=O)C1)N1CCN(CC1)C(=O)c1cc(ccc1F)CN1COCC1 | 0.845 | 0.672 | -1.151 | 1 | 0.434 | 3.057 | -8.401 |
| O=C(C1CS(=O)(=O)C1)N1CCN(CC1)C(=O)c1cc(ccc1F)CN1CCCC1=O | 0.844 | 0.674 | -1.193 | 1 | 0.458 | 2.499 | -6.946 |
| O=C(C1CS(=O)(=O)C1)N1CCN(CC1)C(=O)c1cc(ccc1F)CN1CCOCC1 | 0.843 | 0.656 | -1.227 | 1 | 0.451 | 2.427 | -7.014 |
| O=C1NCCN1Cc1ccc(c(c1)C(=O)N1CCN(CC1)C1CCS(=O)(=O)C1)F | 0.842 | 0.751 | -1.054 | 1 | 0.385 | 3.057 | -6.905 |
| O=C(N1CCN(CC1)Cc1cc(ccc1F)C(=O)N1CCS(=O)(=O)C1)C1CC1 | 0.842 | 0.732 | -0.818 | 1 | 0.355 | 2.586 | -6.804 |
| O=C(C1CCS(=O)(=O)C1)N1CCN(CC1)C(=O)c1cc(ccc1F)CN1CCC1=O | 0.840 | 0.631 | -1.227 | 1 | 0.472 | 2.940 | -8.257 |
| O=C1CNCCN1Cc1ccc(c(c1)C(=O)N1CCN(CC1)C1CCS(=O)(=O)C1)F | 0.839 | 0.695 | -0.974 | 1 | 0.370 | 3.080 | -6.936 |
| O=C1CN(CCN1C(=O)C1CC1)C(=O)c1cc(ccc1F)CN1CC[NH2+]CC1 | 0.839 | 0.700 | -1.148 | 1 | 0.432 | 3.275 | -7.062 |
| O=C1NCCN1Cc1ccc(c(c1)C(=O)N1CCN(CC1)CN1CCS1(=O)=O)F | 0.838 | 0.700 | -1.263 | 1 | 0.395 | 2.905 | -7.501 |
| O=C(C1CCS(=O)(=O)C1)N1CCN(C1)C(=O)c1cc(ccc1F)CN1CCOCC1 | 0.837 | 0.672 | -1.264 | 1 | 0.397 | 3.080 | -7.232 |
| OCCN1CCN(CC1)C(=O)c1cc(ccc1F)CN1CCNC(=O)C1=O | 0.851 | 0.644 | -1.050 | 1 | 0.411 | 2.449 | -9.306 |
| OCCN1CCN(CC1)C(=O)c1cc(ccc1F)CC(=O)N1CCOCC1 | 0.851 | 0.778 | -0.770 | 1 | 0.370 | 2.187 | -8.172 |
| OCCN1CCN(CC1)Cc1ccc(c(c1)C(=O)N1CCN(C(=O)C1)CCO)F | 0.850 | 0.615 | -0.622 | 1 | 0.365 | 2.396 | -7.934 |
| OCCN1CCN(CC1=O)C(=O)c1cc(ccc1F)CN1CCNCC1 | 0.849 | 0.737 | -0.670 | 1 | 0.355 | 2.418 | -6.824 |
| OCCN1CCN(CC1)C(=O)c1cc(CCC(=O)N2CCNCC2)ccc1F | 0.849 | 0.710 | -0.941 | 1 | 0.384 | 2.295 | -8.751 |
| OCCN1CCN(CC1)C(=O)c1cc(ccc1F)CN1CCNC(=O)CC1 | 0.849 | 0.748 | -1.021 | 1 | 0.392 | 2.295 | -7.577 |
| OCCN1CCN(CC1)C(=O)c1cc(CCN2CCNC(=O)C2)ccc1F | 0.847 | 0.701 | -1.019 | 1 | 0.387 | 2.362 | -8.573 |
| OCc1ccc(cc1C(=O)N1CCN(CC1)C(=O)[O-])Cc1n[nH]ccc1=O | 0.850 | 0.706 | -1.022 | 1 | 0.403 | 3.099 | -8.642 |
| OCc1ccc(cc1C(=O)N1CCN(CC1)C(=O)[O-])Cc1n[nH]ncc1=O | 0.848 | 0.654 | -1.051 | 1 | 0.423 | 3.098 | -7.512 |
| OCc1ccc(cc1C(=O)N1CCN(CC1)C#C)Cc1n[nH]ncc1=O | 0.846 | 0.725 | -1.038 | 1 | 0.425 | 3.217 | -6.752 |
| OCc1ccc(cc1C(=O)N1CCN(CC1)CC(=O)[O-])Cc1n[nH]ccc1=O | 0.844 | 0.610 | -1.002 | 1 | 0.419 | 3.098 | -6.311 |
| [NH3+]Cc1ccc(cc1C(=O)N1CCN(CC1)C(=O)[O-])Cc1n[nH]ccc1=O | 0.840 | 0.653 | -0.856 | 1 | 0.384 | 3.504 | -7.692 |
| OCC(=O)N1CCN(CC1)C(=O)c1cc(ccc1C[O-])Cc1n[nH]ccc1=O | 0.839 | 0.644 | -0.943 | 1 | 0.368 | 3.123 | -8.851 |
| OCc1ccc(cc1C(=O)N1CCN(CC1)COC)Cc1n[nH]ccc1=O | 0.836 | 0.752 | -1.369 | 1 | 0.400 | 2.906 | -8.918 |
| OCC(=O)N1CCN(CC1)C(=O)c1cc(ccc1[O-])Cc1n[nH]ccc1=O | 0.834 | 0.688 | -1.157 | 1 | 0.384 | 3.157 | -8.491 |
| OCc1ccc(cc1C(=O)N1CCN(CC1)C(=O)[O-])Cc1ccc(=O)[nH]n1 | 0.834 | 0.706 | -1.405 | 1 | 0.403 | 2.854 | -8.688 |
| OCc1ccc(cc1C(=O)N1CCN(CC1)C=O)Cc1n[nH]ncc1=O | 0.834 | 0.674 | -1.408 | 1 | 0.431 | 2.989 | -6.785 |
| OCCN1CCN(CC1)C(=O)c1cc(ccc1F)CN1CC[NH2+]CC1 | 0.867 | 0.711 | -0.535 | 1 | 0.380 | 3.016 | -6.702 |
| OCC(=O)N1CCN(CC1)C(=O)c1cc(ccc1F)CN1CC[NH2+]CC1 | 0.850 | 0.679 | -0.713 | 1 | 0.366 | 3.063 | -7.065 |
| OCC(=O)N1CCN(CC1)Cc1ccc(c(c1)C(=O)N1CC[NH2+]CC1)F | 0.848 | 0.679 | -0.895 | 1 | 0.380 | 3.063 | -6.692 |
| OCC(=O)N1CCN(CC1)C(=O)c1cc(ccc1F)Cn1[nH]ccc1=O | 0.847 | 0.776 | -1.085 | 1 | 0.394 | 2.619 | -7.887 |
| OCCn1c([O-])nnc(c1=O)Cc1ccc(c(c1)C(=O)N1CCN(CC1)C(=O)C)F | 0.846 | 0.641 | -1.156 | 1 | 0.427 | 2.903 | -7.204 |
| CC(=O)N1CCN(CC1)C(=O)c1cc(ccc1F)Cc1n[nH]c(=O)n1F | 0.843 | 0.849 | -1.214 | 1 | 0.485 | 2.670 | -7.238 |
| OCCn1nc(n(c1=O)C)Cc1ccc(c(c1)C(=O)N1CCN(CC1)C(=O)C1CC1)F | 0.839 | 0.694 | -1.308 | 1 | 0.543 | 2.572 | -7.907 |
| CN(CCn1c(n[nH]c1=O)Cc1ccc(c(c1)C(=O)N1CCN(CC1)C(=O)C)F)C | 0.837 | 0.722 | -1.339 | 1 | 0.440 | 2.546 | -7.441 |
| OCCn1c(n[nH]c1=O)Cc1ccc(c(c1)C(=O)N1CCN(CC1)C(=O)C1CC1)F | 0.832 | 0.687 | -1.440 | 1 | 0.603 | 2.537 | -8.277 |
| O=C(N1CC1)N1CCN(CC1)C(=O)c1cc(ccc1F)Cc1n[nH]c(=O)[nH]c1=O | 0.827 | 0.663 | -1.541 | 1 | 0.587 | 2.570 | -7.933 |
| OCCn1c(=O)[nH]nc(c1=O)Cc1ccccc1C(=O)N1CCN(CC1)C(=O)C1CC1 | 0.827 | 0.622 | -1.464 | 1 | 0.466 | 2.552 | -7.078 |
| OCCn1c(n[nH]c1=O)Cc1ccc(cc1)C(=O)N1CCN(CC1)C(=O)C1CC1 | 0.826 | 0.702 | -1.558 | 1 | 0.411 | 2.379 | -7.761 |
| OCCn1c(n[nH]c1=O)Cc1ccc(c(c1)C(=O)N1CCN(CC1)C(=O)C)F | 0.824 | 0.720 | -1.608 | 1 | 0.458 | 2.505 | -9.443 |
| O=CN1CCN(CC1)CCN1CCN(C1)C(=O)c1cc(C[NH3+])ccc1F | 0.871 | 0.661 | -0.550 | 1 | 0.394 | 3.083 | -7.716 |
| [NH3+]Cc1ccc(c(c1)C(=O)N1CCN(C(=O)C1)CCN1CCN(CC1)C)F | 0.870 | 0.721 | -0.287 | 1 | 0.360 | 2.802 | -7.430 |
| O=CN1CCN(CC1)CCN1CCN(C1)C(=O)c1cc(ccc1F)CN1CCNC1=O | 0.863 | 0.606 | -0.543 | 1 | 0.390 | 2.867 | -8.234 |
| O=CN1CCN(CC1)CCN1CCN(C1)C(=O)c1cc(CO)ccc1F | 0.862 | 0.709 | -0.782 | 1 | 0.394 | 2.627 | -7.658 |
| CC(=O)N1CCN(CC1)C(=O)c1cc(ccc1F)CN1CC[NH2+]C1=O | 0.861 | 0.809 | -0.844 | 1 | 0.400 | 3.012 | -7.754 |
| [NH3+]Cc1ccc(c(c1)C(=O)N1CCN(C1=O)CCN1CCN(CC1)C)F | 0.861 | 0.778 | -0.188 | 1 | 0.329 | 2.957 | -8.408 |
| CC(=O)N1CCN(CC1)C(=O)c1cc(ccc1F)Cc1cn(c(=O)n1C)C | 0.865 | 0.797 | -0.776 | 1 | 0.420 | 2.474 | -7.852 |
| CC(C(=O)N1CCOCC1)N1CCN(CC1)C(=O)c1cc(ccc1F)Cc1n[nH]c(=O)n1C | 0.862 | 0.665 | -0.780 | 1 | 0.418 | 3.070 | -7.831 |
| [O-]C(=O)N1CCN(CC1)Cc1ccc(c(c1)C(=O)N1CCN(CC1)C(=O)[O-])F | 0.862 | 0.612 | -0.704 | 1 | 0.422 | 2.797 | -5.750 |
| OCCN1CCN(C1=O)Cc1ccc(c(c1)C(=O)N1CCN(CC1)CC(O)C)F | 0.867 | 0.673 | -0.673 | 1 | 0.395 | 2.946 | -8.263 |
| CN1CCN(CC1)C(=O)c1cc(ccc1F)CN1CCN(C(=O)C1)C(=O)C | 0.866 | 0.764 | -0.729 | 1 | 0.397 | 2.364 | -8.026 |
| OCCN1C(=O)CCC1C(=O)N1CCN(CC1)C(=O)c1ccccc1F | 0.846 | 0.822 | -0.929 | 1 | 0.375 | 2.703 | -7.053 |
| OCC(=O)N1CCN(CC1)C(=O)c1cc(ccc1F)CN1CCC1=O | 0.838 | 0.766 | -1.235 | 1 | 0.391 | 2.248 | -7.373 |
| O=C1CCC(NN1)C(=O)N1CCN(CC1)C(=O)c1ccccc1F | 0.837 | 0.791 | -1.334 | 1 | 0.403 | 2.858 | -7.493 |
| O=C1CCC(N1)C(=O)N1CCN(CC1)C(=O)c1ccccc1F | 0.833 | 0.857 | -1.428 | 1 | 0.422 | 2.536 | -8.360 |
| O=C(c1ccc(=O)[nH]c1F)N1CCN(CC1)C(=O)C1CC1 | 0.830 | 0.792 | -1.478 | 1 | 0.429 | 2.362 | -8.287 |
| O=C(N1CCNC1=O)CN1CCN(CC1)C(=O)c1ccccc1F | 0.830 | 0.853 | -1.221 | 1 | 0.371 | 2.135 | -7.054 |
| O=C1CCC(N1)C(=O)N1CCN(CC1)Cc1ccccc1C(=O)N1CC1 | 0.829 | 0.775 | -1.229 | 1 | 0.371 | 2.639 | -7.799 |
| O=C1CCC(N1)C(=O)N1CCN(CC1)C(=O)c1n[nH]c(=O)c2c1cccc2 | 0.829 | 0.740 | -1.501 | 1 | 0.492 | 2.823 | -10.613 |
| O=C(N1CCN(CC1)C(=O)c1ccc[nH]c1=O)C1CC1 | 0.829 | 0.830 | -1.514 | 1 | 0.435 | 2.083 | -7.229 |
| O=C(N1CCN(CC1)CCN1NC(=O)C1=O)Cc1n[nH]c(=O)c2c1cccc2 | 0.827 | 0.605 | -1.228 | 1 | 0.384 | 2.762 | -10.272 |
| CN1CC(C1)N(N1CCN(C1)C(=O)c1cc(ccc1F)Cc1n[nH]c(=O)n1C)C | 0.871 | 0.739 | -0.557 | 1 | 0.418 | 3.169 | -8.386 |
| CN1CCN(CC1)C1CN(C1)C(=O)c1cc(ccc1F)Cc1n[nH]c(=O)[nH]1 | 0.860 | 0.780 | -0.879 | 1 | 0.427 | 2.771 | -7.961 |
| CN1CCN(C1)C(=O)C1CN(C1)C(=O)c1cc(ccc1F)Cc1n[nH]c(=O)[nH]1 | 0.858 | 0.744 | -0.910 | 1 | 0.486 | 2.991 | -8.931 |
| CN(C1CN(C1)CCN1CCN(C1)C(=O)c1cc(ccc1F)Cc1n[nH]c(=O)[nH]1)C | 0.858 | 0.648 | -0.904 | 1 | 0.436 | 3.025 | -7.789 |
| CN1CC(C1)N(C1CN(C1)C(=O)c1cc(ccc1F)Cc1n[nH]c(=O)[nH]c1=O)C | 0.858 | 0.683 | -0.918 | 1 | 0.479 | 2.986 | -8.457 |
| CN1CN(N(C1)CCN1CCN(C1)C(=O)c1cc(ccc1F)Cc1n[nH]c(=O)[nH]1)C | 0.856 | 0.656 | -0.702 | 1 | 0.423 | 3.464 | -9.858 |
| CN1CCN(CC1)C1CN(C1)C(=O)c1cc(ccc1F)Cc1n[nH]c(=O)[nH]c1=O | 0.855 | 0.698 | -0.963 | 1 | 0.493 | 2.699 | -7.461 |
| CN(C1CN(C1)C)C1CN(C1)C(=O)c1cc(ccc1F)Cc1n[nH]c(=O)[nH]1 | 0.853 | 0.763 | -0.967 | 1 | 0.413 | 3.081 | -7.793 |
| CN1CCN(C1)C(=O)N(C1CN(C1)C(=O)c1cc(ccc1F)Cc1n[nH]c(=O)[nH]1)C | 0.852 | 0.723 | -0.934 | 1 | 0.425 | 3.162 | -8.599 |
| OCCN1CC(CC1=O)C(=O)N1CCN(CC1)C(=O)c1cc(F)ccc1F | 0.845 | 0.802 | -1.183 | 1 | 0.403 | 2.711 | -7.857 |
| OCCN1C(=O)CN(CC1=O)C(=O)C1CCN(CC1)C(=O)c1ccccc1F | 0.840 | 0.721 | -1.217 | 1 | 0.394 | 2.335 | -8.161 |
| OCCN1CCN(CC1=O)C(=O)C1CCN(CC1)C(=O)c1ccccc1F | 0.839 | 0.821 | -1.303 | 1 | 0.403 | 2.230 | -7.282 |
| OCCN1CC(=O)CC(CC1=O)C(=O)N1CCN(CC1)C(=O)c1cc(F)ccc1F | 0.838 | 0.738 | -1.310 | 1 | 0.405 | 3.000 | -6.568 |
| COCC(=O)N1CC(=O)NC(C1)C(=O)N1CCN(CC1)C(=O)c1cc(F)ccc1F | 0.838 | 0.684 | -1.263 | 1 | 0.395 | 2.964 | -6.416 |
| OCCN1CC(CC1=O)C(=O)N1CCN(CC1)C(=O)c1ccccc1F | 0.838 | 0.813 | -1.272 | 1 | 0.394 | 2.600 | -7.316 |
| O=CN1CCN(CC1=O)C(=O)C1CCN(CC1)C(=O)c1cc(F)ccc1F | 0.836 | 0.723 | -1.365 | 1 | 0.403 | 2.527 | -7.393 |
| OCC(=O)N1CCC(C1)C(=O)N1CCN(CC1)C(=O)c1ccccc1F | 0.834 | 0.817 | -1.406 | 1 | 0.412 | 2.534 | -7.375 |
| OCCN1CCC(CC1=O)C(=O)N1CCN(CC1)C(=O)c1ccccc1F | 0.832 | 0.821 | -1.434 | 1 | 0.408 | 2.638 | -7.038 |
| OCCN1CCN(CC1)C(=O)C(=O)C1CCN(CC1)C(=O)c1cc(F)ccc1F | 0.832 | 0.718 | -1.203 | 1 | 0.375 | 2.383 | -8.198 |
| CC(=O)N1CCN(CC1)C(=O)c1cc(ccc1F)Cc1nnn(n1)C | 0.863 | 0.795 | -0.800 | 1 | 0.435 | 2.437 | -7.140 |
| [O-]C(=O)C(N1CCN(CC1)C(=O)c1cc(ccc1F)Cc1n[nH]c(=O)n1C)C | 0.856 | 0.681 | -0.811 | 1 | 0.446 | 3.261 | -7.645 |
| CC(=O)N1CCN(CCN(C1=O)C)C(=O)c1cc(ccc1F)Cc1n[nH]c(=O)n1C | 0.854 | 0.765 | -0.995 | 1 | 0.452 | 2.784 | -7.375 |
| O=C(N1CCN(C1)C(=O)C)N1CCN(CC1)C(=O)c1cc(ccc1F)Cc1n[nH]c(=O)n1C | 0.852 | 0.685 | -1.023 | 1 | 0.452 | 2.829 | -8.990 |
| CC1CN(CCN1C(=O)N(C)C)C(=O)c1cc(ccc1F)Cc1n[nH]c(=O)n1C | 0.852 | 0.813 | -0.936 | 1 | 0.429 | 3.166 | -7.593 |
| CNC(=O)N1CCN(CC1)C(=O)c1cc(ccc1F)Cc1n[nH]c(=O)n(c1=O)C | 0.851 | 0.701 | -1.046 | 1 | 0.522 | 2.602 | -6.728 |
| CN(C(C(=O)N1CCN(CC1)C(=O)c1cc(ccc1F)Cc1n[nH]c(=O)n1C)C)C | 0.853 | 0.742 | -0.978 | 1 | 0.446 | 3.074 | -8.036 |
| [O-]C(=O)C(N1CCN(CC1)C(=O)c1cc(ccc1F)CCc1n[nH]c(=O)n1C)C | 0.850 | 0.656 | -0.931 | 1 | 0.403 | 3.258 | -8.684 |
| CN1CCN(CCN(C1)C(=O)C)C(=O)c1cc(ccc1F)Cc1n[nH]c(=O)n1C | 0.850 | 0.779 | -1.075 | 1 | 0.458 | 2.744 | -7.855 |
| CC(=O)N1CCN(CC1)C(=O)c1cc(ccc1F)Cc1nnc(n1C)C(=O)[O-] | 0.850 | 0.679 | -1.053 | 1 | 0.397 | 2.798 | -7.907 |
| CC(=O)N1CCN(CC1)C(=O)c1cc(ccc1F)CC1CNCC(=O)N1C | 0.845 | 0.812 | -1.094 | 1 | 0.392 | 2.982 | -8.498 |
| CC(=O)N1CCN(CCC1C(=O)[O-])C(=O)c1cc(ccc1F)Cc1n[nH]c(=O)n1C | 0.845 | 0.653 | -0.957 | 1 | 0.455 | 3.391 | -8.716 |
| COC(=O)C(N1CCN(CC1)C(=O)c1cc(ccc1F)Cc1n[nH]c(=O)n1C)C | 0.844 | 0.712 | -1.191 | 1 | 0.434 | 2.980 | -7.306 |
| O=C1N(CCN1Cc1n[nH]c(=O)c2c1cccc2)CC(=O)N1CCN(CC1)C(=O)C | 0.845 | 0.751 | -1.065 | 1 | 0.389 | 2.577 | -9.948 |
| O=C(N1CCN(CC1)C(=O)C)CN1CCN(CC1)Cc1n[nH]c(=O)c2c1cccc2 | 0.845 | 0.743 | -1.172 | 1 | 0.406 | 2.346 | -7.200 |
| O=C1CN(CCN1Cc1n[nH]c(=O)c2c1cccc2)CCN1CCN(CC1)C(=O)C | 0.844 | 0.727 | -1.032 | 1 | 0.384 | 2.547 | -9.691 |
| CC(=O)N1CCN(CCC1=O)CCN1CCN(CC1)C(=O)c1n[nH]c(=O)c2c1cccc2 | 0.844 | 0.699 | -1.168 | 1 | 0.397 | 2.510 | -10.547 |
| O=C(N1CCN(CC1)CCN1CCC(=O)N(CC1)C(=O)C)Cc1n[nH]c(=O)c2c1cccc2 | 0.843 | 0.660 | -1.091 | 1 | 0.387 | 2.589 | -9.387 |
| O=C1N(CCN1Cc1n[nH]c(=O)c2c1cccc2)CCN1CCN(CC1)C(=O)C | 0.843 | 0.783 | -1.121 | 1 | 0.389 | 2.584 | -10.090 |
| O=C(N1CCN(C1)CCN1CCN(CC1)C(=O)C)Cc1n[nH]c(=O)c2c1cccc2 | 0.842 | 0.727 | -1.236 | 1 | 0.414 | 2.546 | -10.081 |
| O=C1CN(CCN1CCN1CCN(CC1)C(=O)C)Cc1n[nH]c(=O)c2c1cccc2 | 0.841 | 0.727 | -1.047 | 1 | 0.378 | 2.536 | -9.475 |
| O=C(N1CCN(CC1)CCN1CCN(CC1)C(=O)C)Cc1n[nH]c(=O)c2c1cccc2 | 0.841 | 0.710 | -1.253 | 1 | 0.412 | 2.364 | -9.360 |
| CC(N1CCN(CC1)C(=O)C)CN1CCN(CC1)Cc1n[nH]c(=O)c2c1cccc2 | 0.841 | 0.775 | -1.264 | 1 | 0.403 | 2.929 | -10.939 |
| OCCN1CCN(CC1)C(=O)c1cc(ccc1F)CN1CCC(C1=O)O | 0.860 | 0.747 | -0.876 | 1 | 0.417 | 2.839 | -8.079 |
| OCCN1CCN(CC1)C(=O)c1cc(ccc1F)CN1CC(C(C1)O)O | 0.857 | 0.626 | -0.852 | 1 | 0.426 | 3.034 | -8.348 |
| OCCN1CCN(CC1)Cc1ccc(c(c1)C(=O)N1CCN(CC1)O)F | 0.854 | 0.769 | -0.900 | 1 | 0.391 | 2.375 | -7.228 |
| OCCN1CCN(CC1)C(=O)N1CCN(CC1)C(=O)c1cc(CCO)ccc1F | 0.853 | 0.708 | -0.979 | 1 | 0.397 | 2.316 | -6.882 |
| OCCN1CCN(CC(=O)C1=O)C(=O)c1cc(ccc1F)Cc1n[nH]ccc1=O | 0.823 | 0.641 | -1.624 | 1 | 0.429 | 3.009 | -8.849 |
| OCCN1CCN(CC1=O)C(=O)c1cc(ccc1F)Cc1n[nH]c(=O)cc1[O-] | 0.823 | 0.664 | -1.614 | 1 | 0.467 | 3.039 | -8.086 |
| OCc1ccc(c(c1)C(=O)N1CCN(CC1)CCN1CCNC(=O)CC1=O)F | 0.822 | 0.655 | -1.523 | 1 | 0.387 | 2.524 | -9.977 |
| OCCN1CCN2C(C1=O)CN(CC2)C(=O)c1cc(ccc1F)Cc1n[nH]ccc1=O | 0.820 | 0.661 | -1.446 | 1 | 0.425 | 3.433 | -7.906 |
| OCCN1CCN(CC(=O)C1=O)C(=O)c1cc(ccc1F)Cc1ncc[nH]c1=O | 0.820 | 0.641 | -1.685 | 1 | 0.418 | 2.829 | -7.860 |
| [O-]C(=O)C(=O)N1CCN(CC1)C(=O)c1cc(ccc1F)Cc1n[nH]ccc1=O | 0.820 | 0.641 | -1.678 | 1 | 0.493 | 3.018 | -7.474 |
| OCC(=O)C(=O)N1CCN(CC1)C(=O)Cc1n[nH]c(=O)c2c1cccc2 | 0.820 | 0.655 | -1.695 | 1 | 0.412 | 2.504 | -9.324 |
| O=CN1CCN(CC1)C(=O)C1CN(C1)C(=O)c1cc(ccc1F)Cc1n[nH]ccc1=O | 0.819 | 0.670 | -1.698 | 1 | 0.542 | 3.005 | -8.975 |
| OCCNC(=O)CN1CCN(C1)C(=O)c1cc(ccc1F)Cc1ncccc1F | 0.818 | 0.712 | -1.725 | 1 | 0.405 | 2.600 | -8.333 |
| OCCN1CCN(CC1)C(=O)c1cc(ccc1F)Cc1n[nH]c(=O)ccc1=O | 0.818 | 0.723 | -1.727 | 1 | 0.529 | 2.533 | -7.887 |
| CN1CCN(CC1)C(=O)C1CCN(CC1)C(=O)c1ccc[nH]c1=O | 0.849 | 0.827 | -0.871 | 1 | 0.377 | 2.195 | -5.839 |
| OCCN1CCN(CC1)C(=O)C1CCN(CC1)C(=O)c1ccc[nH]c1=O | 0.849 | 0.743 | -1.051 | 1 | 0.394 | 2.266 | -7.095 |
| OCCN1CCN(CCN(CC1)C(=O)c1ccc[nH]c1=O)C(=O)C1CC1 | 0.844 | 0.743 | -1.190 | 1 | 0.420 | 2.680 | -7.499 |
| OCC(=O)N1CCN(CC1)C(=O)C1CCN(CC1)C(=O)c1ccc[nH]c1=O | 0.841 | 0.694 | -1.129 | 1 | 0.386 | 2.333 | -8.106 |
| CCN1CCN(CC1)C(=O)C1CCN(CC1)C(=O)c1ccc[nH]c1=O | 0.837 | 0.859 | -1.346 | 1 | 0.400 | 2.202 | -6.411 |
| O=C(c1ccc(=O)[nH]n1)N1CCN(CC1)C(=O)C1CC1 | 0.836 | 0.786 | -1.353 | 1 | 0.413 | 2.181 | -7.620 |
| O=C(C1CC1)N1CCCN(CC1)C(=O)c1ccc[nH]c1=O | 0.836 | 0.859 | -1.366 | 1 | 0.415 | 2.122 | -7.218 |
| O=C(N1CCN(CC1)C(=O)C1CC1)N1CCN(CC1)C(=O)c1ccc[nH]c1=O | 0.835 | 0.760 | -1.376 | 1 | 0.415 | 2.333 | -7.443 |
| OCc1cc(C(=O)N2CCN(CC2)C(=O)C2CC2)c(=O)[nH]c1 | 0.835 | 0.792 | -1.383 | 1 | 0.460 | 2.322 | -6.931 |
| OCN1CCC(CC1)C(=O)N1CCN(CC1)C(=O)c1ccc[nH]c1=O | 0.833 | 0.754 | -1.331 | 1 | 0.391 | 2.366 | -7.030 |
| OCC(=O)N1CCN(CC1)C(=O)c1cc(n[nH]c1=O)c1ccc(c(c1)CO)F | 0.837 | 0.637 | -1.325 | 1 | 0.417 | 2.479 | -9.189 |
| OCC(=O)N1CCN(CC1)C(=O)c1cc(ccc1F)c1ccc(=O)[nH]n1 | 0.832 | 0.792 | -1.399 | 1 | 0.394 | 2.345 | -8.200 |
| OCC(C(=O)N1CCN(CC1)C(=O)c1cc(ccc1F)c1cc(F)c(=O)[nH]n1)O | 0.832 | 0.617 | -1.302 | 1 | 0.431 | 3.078 | -10.180 |
| OCCN1CCN(CC1)C(=O)c1cc(n[nH]c1=O)c1ccc(c(c1)CO)F | 0.831 | 0.668 | -1.464 | 1 | 0.411 | 2.416 | -8.253 |
| OCc1ccc(cc1C(=O)N1CCOCC1)Cc1n[nH]c(=O)c(=O)ccc1=O | 0.828 | 0.649 | -1.532 | 1 | 0.457 | 2.795 | -6.603 |
| OCC(=O)N1CCN(CC1)C(=O)c1cc(ccc1F)CN1CCC(CCC1=O)C(=O)CO | 0.827 | 0.621 | -1.456 | 1 | 0.400 | 3.010 | -8.475 |
| OCCn1nc([nH]c1=O)Cc1ccc(c(c1)C(=O)N1CCN(CC1)C(=O)CO)F | 0.826 | 0.538 | -1.078 | 1 | 0.413 | 2.789 | -8.568 |
| OCN1CCN(CC1)C(=O)c1cc(n[nH]c1=O)c1ccc(c(c1)CO)F | 0.823 | 0.694 | -1.625 | 1 | 0.423 | 2.524 | -8.274 |
| OCc1ccc(cc1C(=O)N1CCN(CC1)C(=O)C)Cc1n[nH]ccc1=O | 0.823 | 0.797 | -1.627 | 1 | 0.408 | 2.747 | -8.871 |
| OCCN1CCN(CC1)C(=O)c1cc(ccc1F)CN1CC[NH2+]CC1=O | 0.864 | 0.664 | -0.443 | 1 | 0.368 | 3.073 | -7.725 |
| OCCN1CCN(CC1)C(=O)c1cc(ccc1F)CN1CCNC1=O | 0.859 | 0.790 | -0.899 | 1 | 0.408 | 2.346 | -7.938 |
| OCCN1CCN(CC1)Cc1ccc(c(c1)C(=O)N1CC[NH2+]CC1)F | 0.856 | 0.711 | -0.629 | 1 | 0.366 | 3.017 | -7.644 |
| OCCN1CCN(CC1)C(=O)c1cc(CC2CNC2=O)ccc1F | 0.854 | 0.735 | -0.982 | 1 | 0.429 | 2.865 | -8.677 |
| O=C(c1cc(ccc1F)CN1NC(=O)C1=O)N1CCN(CC1)C1CC1 | 0.839 | 0.786 | -1.306 | 1 | 0.423 | 2.617 | -8.294 |
| OCC(=O)N1CCN(CC1)C(=O)c1cc(ccc1F)Cc1cc([O-])c(=O)[nH]n1 | 0.838 | 0.679 | -1.310 | 1 | 0.515 | 2.889 | -8.382 |
| O=C1CNN(N1)Cc1cc(F)ccc1C(=O)N1CCN(CC1)C(=O)C1CC1 | 0.835 | 0.766 | -1.302 | 1 | 0.392 | 2.873 | -7.333 |
| OCC(=O)N1CCN(CC1)Cc1ccc(c(c1)C(=O)N1CCNC1=O)F | 0.834 | 0.760 | -1.062 | 1 | 0.365 | 2.414 | -7.630 |
| O=C1NNN(C1=O)Cc1ccc(c(c1)C(=O)N1CCN(CC1)CC1CC1)F | 0.834 | 0.731 | -1.396 | 1 | 0.438 | 2.762 | -6.292 |
| O=C(c1cc(ccc1F)CN1NC(=O)C(=O)N1)N1CCN(CC1)C1CC1 | 0.832 | 0.748 | -1.434 | 1 | 0.463 | 2.878 | -7.435 |
| OCC(=O)N1CCN(CC1)Cc1cc(ccc1F)C(=O)N1CCN(C1=O)C(=O)C1CC1 | 0.832 | 0.717 | -0.822 | 1 | 0.333 | 2.623 | -6.709 |
| O=C(N1CCN(CC1)C(=O)c1ccc(c(c1)CN1NC(=O)C1=O)F)C1CC1 | 0.831 | 0.745 | -1.462 | 1 | 0.403 | 2.585 | -7.621 |
| O=C(N1CCN(CC1)C(=O)c1ccc(c(c1)CN1C(=O)C1=O)F)C1CC1 | 0.831 | 0.718 | -1.464 | 1 | 0.412 | 2.137 | -7.899 |
| OCC(=O)N1CCN(CC1)C(=O)c1cc(ccc1F)CN1CC(=O)NC1=O | 0.830 | 0.664 | -1.378 | 1 | 0.389 | 2.444 | -8.258 |
| [O-]C(=O)C1CCN(CC1)Cc1ccc(c(c1)C(=O)N1CCN(CC1)C(=O)C)F | 0.873 | 0.728 | -0.609 | 1 | 0.420 | 2.461 | -7.786 |
| [NH]Cc1ccc(c(c1)C(=O)N1CCN(CC1)CC(=O)N1CCN(CC1)C(=O)C)F | 0.865 | 0.710 | -0.640 | 1 | 0.386 | 2.485 | -7.694 |
| [O-]C(=O)CCN1CCN(CC1)Cc1ccc(c(c1)C(=O)N1CCN(CC1)C(=O)C)F | 0.862 | 0.611 | -0.636 | 1 | 0.394 | 2.515 | -6.654 |
| CC(=O)N1CCN(CC1)C(=O)c1cc(ccc1F)CN1CCN(C1=O)C(=O)[O-] | 0.862 | 0.704 | -0.551 | 1 | 0.370 | 2.698 | -7.031 |
| CC(=O)C(N1CCN(CC1)C(=O)c1cc(ccc1F)Cc1n[nH]c(=O)c(=O)n1C)C | 0.854 | 0.683 | -0.954 | 1 | 0.486 | 3.060 | -8.239 |
| OCCN1CCN(CC1)C(=O)c1cc(ccc1F)Cc1n[nH]c(=O)c(=O)n1C | 0.851 | 0.629 | -0.995 | 1 | 0.514 | 2.520 | -7.685 |
| OC(=O)CN1CCN(CC1)C(=O)c1cc(ccc1F)Cc1n[nH]c(=O)c(=O)n1C | 0.849 | 0.613 | -0.962 | 1 | 0.507 | 2.512 | -7.731 |
| CC(=O)CN1CCN(CC1)C(=O)c1cc(ccc1F)Cc1n[nH]c(=O)c(=O)n1C | 0.849 | 0.677 | -1.090 | 1 | 0.507 | 2.566 | -7.167 |
| OCC(N1CCN(CC1)C(=O)c1cc(ccc1F)Cc1n[nH]c(=O)c(=O)n1C)C | 0.849 | 0.639 | -1.059 | 1 | 0.486 | 3.071 | -8.544 |
| CC(=O)N1CCN(CC1)C(=O)c1cc(ccc1F)Cc1nccc(=O)n1C | 0.848 | 0.799 | -1.109 | 1 | 0.423 | 2.385 | -7.772 |
| OCCN1CCN(CC1)C(=O)C1CCN(CC1)C(=O)c1ccc([nH]c1=O)F | 0.850 | 0.687 | -1.064 | 1 | 0.429 | 2.501 | -6.979 |
| OCCN1CCC(C1=O)C(=O)N1CCN(CC1)C(=O)c1ccccc1F | 0.849 | 0.761 | -0.899 | 1 | 0.380 | 2.736 | -7.770 |
| O=C(N1CCN(CC1)CCN1CCN(CC1)C(=O)c1ccc[nH]c1=O)C1CC1 | 0.844 | 0.750 | -1.197 | 1 | 0.439 | 2.278 | -5.279 |
| O=C(C1CN(C1)C(=O)c1ccc[nH]c1=O)N1CCN(CC1)C(=O)C1CC1 | 0.844 | 0.790 | -1.150 | 1 | 0.394 | 2.344 | -7.132 |
| CC(CN1CCN(CC1)C(=O)C1CCN(CC1)C(=O)c1ccc[nH]c1=O)O | 0.843 | 0.756 | -1.225 | 1 | 0.417 | 2.778 | -6.667 |
| OCCN1CCN(CC1)C(=O)c1cc2[nH]nc(c(=O)c2cc1F)CO | 0.839 | 0.673 | -1.199 | 1 | 0.389 | 2.604 | -7.783 |
| O=C(c1ccc([nH]c1=O)[O-])N1CCN(CC1)C(=O)C1CC1 | 0.838 | 0.766 | -1.321 | 1 | 0.406 | 2.606 | -9.330 |
| OCCN1CCC(CC1)C(=O)N1CCN(CC1)C(=O)c1ccc[nH]c1=O | 0.838 | 0.743 | -1.140 | 1 | 0.380 | 2.260 | -7.473 |
| OCCn1c(n[nH]c(=O)c1=O)Cc1ccc(c(c1)C(=O)N1CCOCC1)F | 0.844 | 0.653 | -1.193 | 1 | 0.479 | 2.553 | -7.254 |
| OCCn1[nH]nc(c(=O)c1=O)Cc1ccc(c(c1)C(=O)N1CCOCC1)F | 0.841 | 0.653 | -1.264 | 1 | 0.446 | 2.891 | -7.399 |
| OCCn1c(n[nH]c(=O)c1=O)Cc1ccc(c(c1)C(=O)N1COCC1)F | 0.841 | 0.663 | -1.268 | 1 | 0.447 | 2.801 | -7.099 |
| OCCn1c(=O)[nH]nc(c1=O)Cc1ccc(c(c1)C(=O)N1CCOCCN1C)F | 0.840 | 0.650 | -1.255 | 1 | 0.400 | 3.025 | -6.427 |
| OCCn1c(n[nH]c1=O)Cc1ccc(c(c1)C(=O)N1CCN(CC1)CC)F | 0.838 | 0.743 | -1.320 | 1 | 0.446 | 2.484 | -8.131 |
| OCCn1c(n[nH]c(=O)c1=O)Cc1ccc(c(c1)C(=O)N1CCN(CC1)C)F | 0.838 | 0.635 | -1.304 | 1 | 0.507 | 2.547 | -8.016 |
| OCn1c(n[nH]c(=O)c1=O)Cc1ccc(c(c1)C(=O)N1CCOCC1)F | 0.837 | 0.678 | -1.338 | 1 | 0.493 | 2.625 | -8.864 |
| OCCn1c(n[nH]c(=O)c1=O)Cc1ccc(c(c1)C(=O)N1CCN(C1)CC)F | 0.836 | 0.636 | -1.338 | 1 | 0.461 | 2.765 | -7.693 |
| OCCn1c(=O)[nH]nc(c1=O)Cc1ccc(c(c1)C(=O)N1CCOCC1)F | 0.836 | 0.698 | -1.368 | 1 | 0.479 | 2.566 | -8.149 |
| OCN1CN(CCN(C1=O)CO)C(=O)c1cc(ccc1F)Cc1ncccn1 | 0.845 | 0.758 | -1.168 | 1 | 0.411 | 2.975 | -8.064 |
| OCCN1CCN(CCN(C1=O)C(=O)C)C(=O)c1cc(ccc1F)Cc1ncccn1 | 0.842 | 0.758 | -1.243 | 1 | 0.403 | 2.694 | -7.713 |
| OCCN1CN(CCN(C1=O)CCO)C(=O)c1cc(ccc1F)Cc1ncccn1 | 0.839 | 0.672 | -1.293 | 1 | 0.400 | 2.864 | -8.369 |
| OCCN1C(O)CN(CCC1=O)C(=O)c1cc(ccc1F)Cc1ncccn1 | 0.839 | 0.757 | -1.005 | 1 | 0.380 | 3.177 | -8.297 |
| OCCN1CCN(CCO)CCN(C1)C(=O)c1cc(ccc1F)Cc1ncccn1 | 0.838 | 0.665 | -1.321 | 1 | 0.437 | 2.631 | -8.007 |
| OCCN1CCN(CC1=O)C(=O)c1cc(ccc1F)Cc1ncncn1 | 0.838 | 0.799 | -1.155 | 1 | 0.382 | 2.663 | -7.497 |
| OCCN1CCN(C1)C(=O)c1cc(ccc1F)Cc1nccc(=O)n1CO | 0.837 | 0.714 | -1.333 | 1 | 0.403 | 2.816 | -10.555 |
| OCCN1CCN(CCN(C1=O)C)C(=O)c1cc(ccc1F)Cc1ncccn1 | 0.837 | 0.808 | -1.346 | 1 | 0.419 | 2.593 | -6.893 |
| OCCn1c(n[nH]c(=O)c1=O)Cc1ccc(c(c1)C(=O)N1CCN(C1)C)F | 0.835 | 0.647 | -1.389 | 1 | 0.473 | 2.788 | -7.368 |
| OCC(=O)N1CCN(CC1)c1ccc(c(c1)C(=O)N1CCN(C1)C(=O)C1CC1)F | 0.851 | 0.771 | -1.050 | 1 | 0.408 | 2.486 | -7.008 |
| OCCn1nc(n(c1=O)[O-])Cc1ccc(c(c1)C(=O)N1CCN(CC1)CO)F | 0.850 | 0.631 | -1.027 | 1 | 0.427 | 2.986 | -8.141 |
| OCC(=O)N1CCN(CC1)C(=O)c1cc(ccc1F)c1n[nH]c(=O)n1C | 0.845 | 0.741 | -1.023 | 1 | 0.384 | 2.493 | -9.372 |
| OCc1nnc(n1C)Cc1ccc(c(c1)C(=O)N1CCN(CC1)C(=O)C)F | 0.844 | 0.832 | -1.201 | 1 | 0.435 | 2.447 | -8.510 |
| OCC(=C)N1CCN(C1)C(=O)c1cc(ccc1F)Cc1n[nH]c(=O)n1C | 0.843 | 0.785 | -1.196 | 1 | 0.432 | 3.023 | -8.694 |
| OCCN1CCN(CC1)C(=O)C1CCN(CC1)C(=O)c1c[nH]c(=O)c(c1)F | 0.843 | 0.734 | -1.063 | 1 | 0.384 | 2.416 | -6.861 |
| [O-]C(=O)N1CCN(CC1)C(=O)c1cc(ccc1F)c1n[nH]c(=O)n1C | 0.843 | 0.749 | -0.987 | 1 | 0.375 | 2.780 | -8.678 |
| OCC(N1CCN(CC1)C(=O)c1cc(ccc1F)c1n[nH]c(=O)n1C)CO | 0.843 | 0.616 | -0.983 | 1 | 0.387 | 2.660 | -9.536 |
| OCCN1CCN(CC1)C(=O)Cc1ccc(c(c1)C(=O)N1CCC1CO)F | 0.861 | 0.710 | -0.594 | 1 | 0.373 | 2.784 | -6.854 |
| OCCN1CCN(C1=O)CCc1ccc(c(c1)C(=O)N1CCN(CC1)O)F | 0.857 | 0.739 | -0.701 | 1 | 0.375 | 2.697 | -7.749 |
| OCCN1CCN(CC1)C(=O)c1cc(ccc1F)CN1CCNCCC1=O | 0.864 | 0.748 | -0.701 | 1 | 0.392 | 2.370 | -8.370 |
| O=C1CNCCN1Cc1ccc(c(c1)C(=O)N1CCNCC1)F | 0.862 | 0.810 | -0.747 | 1 | 0.391 | 2.425 | -7.074 |
| OCCN1CCN(CC1)C(=O)c1cc(ccc1F)Cn1c(=O)[nH][nH]c1=O | 0.861 | 0.616 | -0.733 | 1 | 0.414 | 2.566 | -8.125 |
| OCCN1CCN(CC1)C(=O)c1cc(ccc1F)CN1C(=O)CNCC1=O | 0.852 | 0.641 | -1.041 | 1 | 0.403 | 2.432 | -7.883 |
| OCCN1CCN(CC1)C(=O)c1cc(ccc1F)Cn1cn[nH]c1=O | 0.850 | 0.760 | -1.068 | 1 | 0.411 | 2.491 | -8.900 |
| OCCN1CCN(CC1)C(=O)c1cc(ccc1F)c1n[nH]c(=O)[nH]c1=O | 0.850 | 0.641 | -1.047 | 1 | 0.397 | 2.472 | -7.562 |
| OCCN1CCN(CC1=O)C(=O)c1cc(ccc1F)Cc1n[nH]cc1O | 0.833 | 0.700 | -1.422 | 1 | 0.434 | 2.900 | -8.917 |
| OCCN1CCN(CC1)C(=O)c1cc(ccc1F)Cc1ncc[nH]c1=O | 0.833 | 0.798 | -1.424 | 1 | 0.493 | 2.453 | -8.416 |
| O=C1CN(CCN1CCN1CCOCC1)C(=O)c1cc(ccc1F)Cc1n[nH]cc1F | 0.832 | 0.732 | -1.436 | 1 | 0.418 | 2.895 | -7.891 |
| OC1CCN(C1)C(=O)c1cc(ccc1F)CN1CCNC(=O)C1 | 0.832 | 0.820 | -1.193 | 1 | 0.373 | 2.761 | -9.497 |
| OCCN1CCN(CC1)C(=O)c1cc(ccc1F)c1n[nH][nH]c1=O | 0.830 | 0.709 | -1.490 | 1 | 0.403 | 2.599 | -6.787 |
| CCCN1CCN(CC1)C(=O)c1cc(ccc1F)Cc1nn(C)c(=O)[nH]c1=O | 0.829 | 0.798 | -1.505 | 1 | 0.486 | 2.522 | -7.576 |
| O=C1CN(CCN1Cc1n[nH]c(=O)c(c1)O)C(=O)c1ccccc1F | 0.828 | 0.820 | -1.443 | 1 | 0.392 | 2.595 | -8.655 |
| OCCN1CCN(CC1)C(=O)c1cc(ccc1F)Cc1c[nH][nH]c1=O | 0.828 | 0.719 | -1.529 | 1 | 0.451 | 2.628 | -9.722 |
| OCCn1ccc(=O)c(n1)Cc1ccc(c(c1)C(=O)N1CCN(C(=O)C1)C)F | 0.828 | 0.771 | -1.506 | 1 | 0.397 | 2.683 | -8.201 |
| OCCn1c(n[nH]c1=O)Cc1ccc(c(c1)C(=O)N1CCN(CC1)CC=O)F | 0.828 | 0.601 | -1.348 | 1 | 0.440 | 2.679 | -8.130 |
| OCCN1CCN(C1=O)Cc1ccc(c(c1)C(=O)N1CCN(CC1)C(=O)CO)F | 0.859 | 0.650 | -0.605 | 1 | 0.370 | 2.502 | -8.602 |
| OCCN1CCN(CC1)C(=O)c1cc(ccc1F)CN1CCN(CC1)CC=O | 0.859 | 0.654 | -0.842 | 1 | 0.394 | 2.325 | -8.218 |
| O=CN1CCN(CC1)Cc1ccc(c(c1)C(=O)N1CCN(CC1)C(=O)C)F | 0.856 | 0.714 | -0.943 | 1 | 0.406 | 2.329 | -7.867 |
| OCCN1CCN(CC1)C(=O)c1cc(ccc1F)CN1CCN(CC1)C(=O)O | 0.855 | 0.751 | -0.884 | 1 | 0.391 | 2.176 | -7.248 |
| OCCN1CCN(CC1=O)c1ccc(c(c1)F)C(=O)N1CCN(CC1)C(=O)C1CC1 | 0.850 | 0.737 | -1.074 | 1 | 0.400 | 2.425 | -5.520 |
| OCCN1CCN(CC1=O)Cc1ccc(c(c1)F)C(=O)N1CCN(CC1)C(=O)C1CC1 | 0.847 | 0.692 | -1.136 | 1 | 0.427 | 2.398 | -7.149 |
| OCCN1CCN(CC1=O)Cc1cc(c(cc1F)F)C(=O)N1CCN(CC1)C(=O)C1CC1 | 0.846 | 0.674 | -1.158 | 1 | 0.400 | 2.551 | -8.006 |
| OCCN1CCN(CC1)C(=O)c1ccc(cc1F)CN1CCN(C1=O)C(=O)C1CC1 | 0.843 | 0.737 | -1.124 | 1 | 0.390 | 2.507 | -6.715 |
| OCCN1CCN(C1=O)c1ccc(c(c1)F)C(=O)N1CCN(CC1)C(=O)C1CC1 | 0.841 | 0.785 | -1.231 | 1 | 0.397 | 2.410 | -4.432 |
| OCC(=O)N1CCN(CC1)c1ccc(c(c1)F)C(=O)N1CCN(CC1)C(=O)C1CC1 | 0.841 | 0.753 | -1.259 | 1 | 0.400 | 2.262 | -5.328 |
| OCCN1CCN(C(=O)C1)c1ccc(c(c1)F)C(=O)N1CCN(CC1)C(=O)C1CC1 | 0.839 | 0.737 | -1.303 | 1 | 0.400 | 2.386 | -5.855 |
| O=C(N1CCN(CC1)C(=O)c1ccc(c(c1)F)CN1CCNC(=O)C1=O)C1CC1 | 0.835 | 0.717 | -1.304 | 1 | 0.392 | 2.443 | -6.387 |
| OCC(=O)N1CCN(CC1)C(=O)c1ccc(cc1F)N1CCN(CC1)C(=O)C1CC1 | 0.835 | 0.753 | -1.383 | 1 | 0.400 | 2.262 | -5.513 |
| OCCN1CCN(CC1)c1ccc(c(c1)C(=O)N1CCN(CC1)C(=O)C(=O)C1CC1)F | 0.832 | 0.647 | -1.287 | 1 | 0.384 | 2.452 | -8.556 |
| CN1CCN(C(=O)C1Cc1ccc(c(c1)C(=O)N1CCN(CC1)C(=O)C)F)C | 0.869 | 0.752 | -0.685 | 1 | 0.408 | 2.932 | -8.091 |
| CC(=O)N1CCN(CC1)C(=O)c1cc(CCC2C(=O)N(C)CCN(C2=O)C)ccc1F | 0.866 | 0.653 | -0.747 | 1 | 0.414 | 2.633 | -8.471 |
| CC(=O)N1CCN(CC1)C(=O)c1cc(CCN2CCN(C(=O)N2)C)ccc1F | 0.864 | 0.813 | -0.785 | 1 | 0.408 | 2.753 | -8.098 |
| CC(=O)N1CCN(CC1)C(=O)c1cc(ccc1F)CCn1nnn(c1=O)C | 0.863 | 0.728 | -0.809 | 1 | 0.400 | 2.467 | -7.719 |
| CC(=O)N1CCN(CC1)C(=O)c1cc(CCN2CCN(C2)C(=O)[O-])ccc1F | 0.861 | 0.697 | -0.793 | 1 | 0.394 | 2.659 | -8.155 |
| O=CN1CCC(C1)N1CCN(CC1)C(=O)Cc1n[nH]c(=O)c2c1cccc2 | 0.847 | 0.757 | -1.091 | 1 | 0.405 | 3.097 | -10.299 |
| O=C(N1CCN(C1)CCN1CCN(CC1)C(=O)N)Cc1n[nH]c(=O)c2c1cccc2 | 0.844 | 0.667 | -1.193 | 1 | 0.408 | 2.612 | -10.625 |
| O=CN1CCN(CC1)CN1CCN(CC1)C(=O)Cc1n[nH]c(=O)c2c1cccc2 | 0.843 | 0.673 | -1.220 | 1 | 0.400 | 2.627 | -9.356 |
| OCCN1CCN(CC1)C(=O)c1cc(ccc1F)Cc1[nH][nH]c(=O)n1 | 0.847 | 0.680 | -1.137 | 1 | 0.451 | 2.577 | -7.599 |
| OCCN1CCN(CC1=O)C(=O)c1cc(ccc1F)Cc1[nH][nH]c(=O)n1 | 0.845 | 0.636 | -1.055 | 1 | 0.390 | 2.796 | -8.093 |
| OCCN1CCN(CC1C)C(=O)c1cc(ccc1F)Cc1n[nH]c(=O)[nH]1 | 0.843 | 0.692 | -1.094 | 1 | 0.429 | 3.241 | -7.861 |
| OCCN1CCN(CC1)C(=O)c1cc(ccc1F)Cc1nc(=O)[nH]c(=O)[nH]1 | 0.842 | 0.610 | -1.106 | 1 | 0.465 | 2.545 | -8.429 |
| OCCN1CCN(CC1)C(=O)c1cc(ccc1F)CC1=NC(=O)C(=O)N1 | 0.841 | 0.666 | -1.250 | 1 | 0.451 | 2.709 | -6.463 |
| OCCN1CCN(CC1)C(=O)c1cc(ccc1F)Cc1nc(=O)[nH][nH]c1=O | 0.841 | 0.610 | -1.121 | 1 | 0.500 | 2.649 | -8.068 |
| OCCN1CCN(CC1)C(=O)c1cc(ccc1F)Cc1[nH]c([O-])cc(=O)n1 | 0.839 | 0.716 | -1.291 | 1 | 0.427 | 2.810 | -8.468 |
| OCCN1CCN(C1)C(=O)c1cc(ccc1F)CC1=NC(=O)C(=O)N1 | 0.839 | 0.674 | -1.307 | 1 | 0.432 | 2.969 | -9.284 |
| OCCN1CCN(C1)C(=O)c1cc(ccc1F)Cc1[nH][nH]c(=O)n1 | 0.838 | 0.685 | -1.311 | 1 | 0.438 | 2.841 | -8.109 |
| CC(=O)N1CCN(CC1)C(=O)c1cc(CCN2CC(=O)N(C2)C)ccc1F | 0.853 | 0.763 | -0.784 | 1 | 0.375 | 2.474 | -7.997 |
| OCCN1CCN(CC1)C(=O)Cc1ccc(c(c1)C(=O)N1CCC(C1)O)F | 0.853 | 0.735 | -0.769 | 1 | 0.373 | 2.692 | -8.409 |
| OCCN1CC(=O)N=C(C1=O)Cc1ccc(c(c1)C(=O)N1CCN(CC1)C)F | 0.850 | 0.736 | -1.031 | 1 | 0.395 | 2.784 | -7.415 |
| OCCN1CC(=O)C(=O)N=C1Cc1ccc(c(c1)C(=O)N1CCNCC1)F | 0.846 | 0.654 | -1.111 | 1 | 0.395 | 2.947 | -7.247 |
| OCCN1CC(=O)N=C1Cc1ccc(c(c1)C(=O)N1CCNCC1)F | 0.846 | 0.760 | -1.165 | 1 | 0.405 | 2.850 | -7.811 |
| OCCN1CC(=O)C(=O)N=C1Cc1ccc(c(c1)C(=O)N1CCN(CC1)C)F | 0.842 | 0.685 | -1.194 | 1 | 0.395 | 2.843 | -8.708 |
| OCCN1CCN(CC1=O)C(=O)Cc1ccc(c(c1)C(=O)N1CC1)F | 0.837 | 0.726 | -1.155 | 1 | 0.380 | 2.286 | -7.309 |
| OCCN1CCN(CC1=O)C(=O)Cc1ccc(c(c1)C(=O)N1CCNCC1)F | 0.835 | 0.676 | -1.140 | 1 | 0.373 | 2.489 | -7.722 |
| O=C1CN(C1)C(=O)Cc1ccc(c(c1)C(=O)N1CCNCC1)F | 0.835 | 0.839 | -1.364 | 1 | 0.397 | 2.390 | -8.034 |
| OCCN1CCC(=O)C(=O)N=C1Cc1ccc(c(c1)C(=O)N1CCNCC1)F | 0.834 | 0.664 | -1.347 | 1 | 0.395 | 2.927 | -8.695 |
| OCCN1CC(=O)N=C(C1=O)Cc1ccc(c(c1)C(=O)N1CCNCC1)F | 0.833 | 0.702 | -1.323 | 1 | 0.390 | 2.887 | -7.410 |
| OCCN1CC(=O)N=C1Cc1ccc(c(c1)C(=O)N1CCOCC1)F | 0.833 | 0.814 | -1.300 | 1 | 0.387 | 2.753 | -7.674 |
| O=C1CN(CCN2CN1CC2)C(=O)c1cc(ccc1F)CCc1[nH]ncn1 | 0.847 | 0.834 | -0.841 | 1 | 0.413 | 3.549 | -9.243 |
| O=C1CN2CCN(CN1CC2)C(=O)c1cc(ccc1F)CCc1ncn[nH]1 | 0.846 | 0.834 | -0.837 | 1 | 0.413 | 3.581 | -6.555 |
| OCC(CN1CCN(CC1=O)C(=O)c1cc(ccc1F)Cc1ncc[nH]1)O | 0.846 | 0.646 | -0.978 | 1 | 0.388 | 3.122 | -8.491 |
| OCCN1CCN2C(C1=O)CN(CC2)C(=O)c1cc(ccc1F)Cc1ncn[nH]1 | 0.846 | 0.698 | -0.955 | 1 | 0.390 | 3.216 | -7.216 |
| OCCN1CCN(CC1=O)C(=O)c1cc(ccc1F)CCc1ncn[nH]1 | 0.845 | 0.754 | -1.022 | 1 | 0.385 | 2.599 | -7.728 |
| O=C(c1cc(ccc1F)Cc1ncn[nH]1)N1CCN2N(CC1)C(=O)CCC2=O | 0.845 | 0.820 | -1.178 | 1 | 0.451 | 2.878 | -7.205 |
| O=C1N2CCN1CCN(C2)C(=O)c1cc(ccc1F)CCc1ncn[nH]1 | 0.845 | 0.879 | -0.936 | 1 | 0.405 | 3.447 | -7.677 |
| OCCN1CCN(CC1=O)C(=O)c1cc(ccc1F)Cc1ccn[nH]1 | 0.844 | 0.821 | -1.187 | 1 | 0.408 | 2.617 | -8.133 |
| OCCN1CC(=O)N2C(C1=O)CN(CC2)C(=O)c1cc(ccc1F)Cc1ncn[nH]1 | 0.844 | 0.646 | -1.012 | 1 | 0.398 | 3.287 | -9.151 |
| FCCN1CCN(CC1=O)C(=O)c1cc(ccc1F)CCc1nnn[nH]1 | 0.843 | 0.800 | -1.219 | 1 | 0.403 | 2.877 | -5.801 |
| CC(=O)N1CCN(CC1)C(=O)c1cc(ccc1F)Cc1cn(C)c(=O)n(c1=O)C | 0.859 | 0.730 | -0.893 | 1 | 0.408 | 2.429 | -7.881 |
| OCCn1c(=O)c(Cc2ccc(c(c2)C(=O)N2CCN(CC2)C(=O)C)F)nn(c1=O)C | 0.858 | 0.644 | -0.906 | 1 | 0.438 | 2.582 | -8.658 |
| CC(=O)N1CCN(CC1)C(=O)c1cc(ccc1F)Cc1cn(C)c(=O)c(=O)n1C | 0.858 | 0.683 | -0.910 | 1 | 0.408 | 2.579 | -7.453 |
| O=C(N1CCN(C(=O)C1)C)Cc1ccc(c(c1)C(=O)N1CCN(CC1)C(=O)C)F | 0.855 | 0.709 | -0.786 | 1 | 0.380 | 2.346 | -8.884 |
| CC(=O)N1CCN(CC1)C(=O)c1cc(ccc1F)Cc1c[nH]c(=O)c(=O)n1C | 0.853 | 0.755 | -1.012 | 1 | 0.437 | 2.602 | -9.040 |
| CC(=O)N1CCN(CC1)Cc1ccc(c(c1)C(=O)N1CCN(C1=O)C(=O)C)F | 0.850 | 0.767 | -0.941 | 1 | 0.386 | 2.469 | -7.808 |
| CC(=O)N1CCN(CC1)C(=O)c1cc(ccc1F)Cc1cc(=O)n(c(=O)n1C)C | 0.850 | 0.730 | -1.083 | 1 | 0.414 | 2.440 | -8.298 |
| CC(=O)N1CCN(CC1)C(=O)c1cc(ccc1F)Cc1cn(C)c(=O)c(=O)n(c1=O)C | 0.848 | 0.610 | -0.972 | 1 | 0.403 | 2.549 | -7.374 |
| CC(=O)N1CCN(CC1)C(=O)c1cc(cnc1F)Cc1n[nH]c(=O)n(c1=O)C | 0.847 | 0.664 | -1.126 | 1 | 0.425 | 2.770 | -8.060 |
| OCc1n[nH]c(=O)cc1C(=O)N1CCN(CC1)C(=O)C1CC1 | 0.844 | 0.741 | -1.197 | 1 | 0.492 | 2.481 | -7.916 |
| O=C(N1CCN(CC1)C(=O)c1cc(c[nH]c1=O)CN1CCNC1=O)C1CC1 | 0.841 | 0.749 | -1.257 | 1 | 0.414 | 2.622 | -7.711 |
| O=C(N1CCN(CC1)C(=O)c1c[nH]c(=O)[nH]c1=O)C1CC1 | 0.839 | 0.711 | -1.301 | 1 | 0.413 | 2.241 | -7.462 |
| OCc1cc(C(=O)N2CCN(CC2)C(=O)C2CC2)c(=O)[nH]n1 | 0.839 | 0.741 | -1.302 | 1 | 0.500 | 2.429 | -6.316 |
| OCCN1CCN(CC1)C(=O)c1n[nH]c(=O)c(c1)C(=O)C1CC1 | 0.838 | 0.682 | -1.001 | 1 | 0.366 | 2.482 | -8.309 |
| OCc1nnccc1C(=O)N1CCN(CC1)C(=O)C1CC1 | 0.836 | 0.823 | -1.351 | 1 | 0.400 | 2.432 | -6.544 |
| OCc1c[nH]c(=O)cc1C(=O)N1CCN(CC1)C(=O)C1CC1 | 0.836 | 0.792 | -1.368 | 1 | 0.409 | 2.351 | -8.683 |
| OCc1nc(C(=O)N2CCN(CC2)C(=O)C2CC2)c(=O)[nH]n1 | 0.835 | 0.690 | -1.385 | 1 | 0.409 | 2.575 | -6.517 |
| OCc1cc(C(=O)N2CCN(CC2)C(=O)C2CCOCC2)c(=O)[nH]n1 | 0.835 | 0.720 | -1.391 | 1 | 0.463 | 2.572 | -7.430 |
| OCCN1CCN(C1=O)CCc1c[nH]nc1Cc1ccc(c(c1)C(=O)N1CCOCC1)F | 0.841 | 0.628 | -1.181 | 1 | 0.407 | 3.034 | -7.943 |
| OCCN1CCN(C1=O)Cc1c[nH]nc1Cc1ccc(c(c1)C(=O)N1CC1)F | 0.837 | 0.688 | -1.331 | 1 | 0.453 | 2.945 | -8.525 |
| OCCN1CCN(C1=O)CCc1c[nH]nc1Cc1ccc(c(c1)C(=O)N1CCC1)F | 0.837 | 0.679 | -1.340 | 1 | 0.423 | 2.995 | -6.680 |
| OCCN1CCN(C1=O)CCc1c[nH]nc1Cc1ccc(c(c1)C(=O)N1CC1)F | 0.836 | 0.642 | -1.370 | 1 | 0.453 | 2.982 | -7.207 |
| OCCN1CCN(CC1)C(=O)c1cc(ccc1F)CC1=NOCCN(C1=O)CCO | 0.832 | 0.604 | -1.272 | 1 | 0.403 | 2.923 | -8.091 |
| OCCN1CCN(CCN(CC1)C(=O)c1ccccc1F)Cc1n[nH]c(=O)c(c1)O | 0.831 | 0.619 | -1.369 | 1 | 0.405 | 2.931 | -7.854 |
| OCCN1CCN(CC1=O)C(=O)c1cc(ccc1F)Cc1n[nH]n(c1=O)CCO | 0.830 | 0.516 | -0.882 | 1 | 0.440 | 3.018 | -8.520 |
| CCCn1c(n[nH]c1=O)Cc1ccc(c(c1)C(=O)N1CCN(CC1)C=O)F | 0.830 | 0.750 | -1.485 | 1 | 0.453 | 2.668 | -8.060 |
| OCCN1CCN(CC1)C(=O)c1cc(ccc1F)Cc1n[nH]cc(c1=O)[O-] | 0.830 | 0.716 | -1.485 | 1 | 0.486 | 2.981 | -7.869 |
| OCCN1CCN(CC1=O)C(=O)c1cc(ccc1F)Cc1ncn[nH]1 | 0.835 | 0.781 | -1.374 | 1 | 0.403 | 2.616 | -7.686 |
| OCCN1CCN(CC1=O)C(=O)c1cc(ccc1F)CCn1nccn1 | 0.835 | 0.778 | -0.915 | 1 | 0.351 | 2.688 | -7.167 |
| OCCc1ccc(c(c1)C(=O)N1CCN(CC1)C(=O)C)F | 0.833 | 0.891 | -1.414 | 1 | 0.406 | 1.997 | -7.530 |
| OCCN1CN(CC1=O)C(=O)c1cc(ccc1F)Cc1ccn[nH]1 | 0.833 | 0.828 | -1.194 | 1 | 0.377 | 2.901 | -7.732 |
| CC1CN(CCN1C(=O)c1cc(ccc1F)Cc1n[nH]c(=O)n1C)C(=O)C | 0.833 | 0.847 | -1.389 | 1 | 0.421 | 3.053 | -7.980 |
| OCCN1CCN(CC1=O)C(=O)c1cc(ccc1F)Cc1nnn[nH]1 | 0.833 | 0.731 | -1.426 | 1 | 0.408 | 2.759 | -6.179 |
| O=C1NCCN(C1)C(=O)CN1CCN(CC1)C(=O)c1cc(ccc1F)Cc1ccn[nH]1 | 0.831 | 0.690 | -1.456 | 1 | 0.410 | 2.656 | -7.656 |
| OCCN1CCN(CC1=O)C(=O)c1cc(ccc1F)Cc1cnn[nH]1 | 0.831 | 0.781 | -1.457 | 1 | 0.427 | 2.961 | -8.922 |
| CNCCN1CCN(CC1)C(=O)c1cc(ccc1F)Cc1n[nH]c(=O)n1C | 0.863 | 0.730 | -0.814 | 1 | 0.465 | 2.516 | -9.573 |
| COCCN1CCN(CC1)C(=O)c1cc(ccc1F)Cc1nn(c(=O)[nH]1)C | 0.856 | 0.780 | -0.952 | 1 | 0.421 | 2.637 | -7.611 |
| CNCN1CCN(CC1)C(=O)c1cc(ccc1F)Cc1n[nH]c(=O)n(c1=O)C | 0.854 | 0.688 | -0.992 | 1 | 0.514 | 2.724 | -7.252 |
| CNC(=O)CN1CCN(CC1)C(=O)c1cc(ccc1F)Cc1n[nH]c(=O)n1C | 0.853 | 0.706 | -1.019 | 1 | 0.458 | 2.483 | -7.827 |
| CNCCN1CCN(CC1=O)C(=O)c1cc(ccc1F)Cc1n[nH]c(=O)n1C | 0.852 | 0.682 | -1.040 | 1 | 0.421 | 2.732 | -7.916 |
| OCCN1CCN(CC1)C(=O)c1cc(ccc1F)CC(=O)N1CCNCC1 | 0.864 | 0.726 | -0.738 | 1 | 0.394 | 2.276 | -8.059 |
| OCCN1CCN(CC1)C(=O)Cc1ccc(c(c1)C(=O)N1CCOCC1)F | 0.859 | 0.778 | -0.601 | 1 | 0.370 | 2.187 | -7.847 |
| OCCN1CCN(CC1)C(=O)c1cc(ccc1F)CC(=O)N1CCC(CC1)[O-] | 0.855 | 0.728 | -0.953 | 1 | 0.397 | 2.650 | -8.670 |
| CN1CCN(CC1)C(=O)c1cc(ccc1C(=O)O)Cc1n[nH]c(=O)c(=O)n1C | 0.852 | 0.654 | -1.039 | 1 | 0.417 | 2.521 | -7.758 |
| CC(=O)N1CCN(CC1)C(=O)c1ccccc1Cc1n[nH]c(=O)n(c1=O)C | 0.851 | 0.772 | -0.793 | 1 | 0.371 | 2.432 | -9.637 |
| CC(=O)N1CCN(CC1)C(=O)c1ccccc1Cc1n[nH]c(=O)c(=O)n1C | 0.850 | 0.723 | -0.948 | 1 | 0.386 | 2.420 | -7.510 |
| CN1CCN(CC1)C(=O)c1cc(ccc1C)Cc1n[nH]c(=O)n(c1=O)C | 0.849 | 0.818 | -1.090 | 1 | 0.423 | 2.453 | -7.768 |
| CC(C(=O)N1CCN(CC1)C(=O)c1cc(ccc1F)Cc1n[nH]c(=O)n1C)O | 0.849 | 0.730 | -1.093 | 1 | 0.465 | 2.983 | -8.043 |
| CN1CCN(CC1)C(=O)c1cc(ccc1F)Cc1n[nH]c(=O)n(c1=O)C | 0.849 | 0.802 | -1.095 | 1 | 0.545 | 2.479 | -6.576 |
| OCCN1CCN(CC1)C(=O)c1cc(ccc1F)Cc1nc(=O)c(=O)[nH]n1C | 0.849 | 0.629 | -1.049 | 1 | 0.452 | 2.692 | -7.674 |
| CC(=O)N1CCN(CC1)C(=O)c1cc(ccc1F)Cc1cc(=O)c(=O)n(c(=O)n1C)C | 0.849 | 0.610 | -0.962 | 1 | 0.408 | 2.585 | -8.005 |
| O=C(C(N1CCN(C1)C(=O)c1cc(ccc1F)Cc1n[nH]c(=O)n1C)C)N1CCOCC1 | 0.847 | 0.682 | -0.872 | 1 | 0.390 | 3.294 | -7.491 |
| OCC(n1ccc(=O)c(n1)Cc1ccc(c(c1)C(=O)N1CCN(CC1)C)F)CO | 0.846 | 0.697 | -1.163 | 1 | 0.427 | 2.739 | -7.720 |
| OCCn1ccc(=O)c(n1)Cc1ccc(c(c1)C(=O)N1CCN(CC1)C(=O)CO)F | 0.841 | 0.637 | -1.239 | 1 | 0.432 | 2.610 | -8.724 |
| OCCN1CCN(C1)C(=O)c1cc(ccc1F)Cc1ncc(=O)n(n1)CCO | 0.841 | 0.627 | -1.204 | 1 | 0.421 | 2.840 | -8.076 |
| OCCn1nc(Cc2ccc(c(c2)C(=O)N2CC[NH2+]CC2)F)c(=O)ccc1=O | 0.840 | 0.637 | -1.070 | 1 | 0.408 | 3.341 | -8.722 |
| OCCN1CCN(CC1=O)Cc1ccc(c(c1)C(=O)N1CCN(C(=O)C1)CCO)F | 0.840 | 0.569 | -0.664 | 1 | 0.370 | 2.599 | -7.862 |
| OC(N1CCN(CC1)C(=O)c1cc(ccc1F)Cc1nccc(=O)n1[O-])O | 0.840 | 0.679 | -1.274 | 1 | 0.425 | 3.005 | -7.429 |
| ON1CCN(CC1)C(=O)c1cc(ccc1F)Cc1nc(=O)c(c([nH]1)[O-])F | 0.839 | 0.783 | -1.234 | 1 | 0.425 | 3.104 | -8.367 |
| OCCN1CCN(CC1=O)C(=O)c1cc(ccc1F)Cc1n[nH]c(=O)n1C | 0.838 | 0.703 | -1.329 | 1 | 0.427 | 2.674 | -7.810 |
| [O-]N1CCN(CC1)C(=O)c1cc(ccc1F)Cc1ccc(=O)n(c1)C(O)O | 0.836 | 0.742 | -1.328 | 1 | 0.403 | 3.072 | -8.705 |
| OCCN1CCN(CC1)C(=O)c1cc(ccc1F)Cc1n[nH]c(=O)c(c1[O-])[O-] | 0.836 | 0.627 | -1.258 | 1 | 0.559 | 3.095 | -7.928 |
| OC(N1CCN(CC1)C(=O)c1cc(ccc1F)Cc1[nH]ccc(=O)n1)O | 0.834 | 0.634 | -1.368 | 1 | 0.431 | 2.822 | -7.748 |
| OCCN1CCN(CC1=O)C(=O)c1cc(ccc1F)Cc1nn(CO)ccc1=O | 0.834 | 0.651 | -1.331 | 1 | 0.392 | 2.840 | -8.220 |
| OCCN1CCN(C1)C(=O)c1cc(ccc1F)Cc1nccc(=O)n1CCO | 0.834 | 0.672 | -1.407 | 1 | 0.408 | 2.748 | -9.069 |
| OCCN1CCN(CC1)C(=O)c1cc(ccc1F)Cc1nccc(=O)n1O | 0.833 | 0.711 | -1.422 | 1 | 0.438 | 2.562 | -8.261 |
| CC(CN1CCN(CC1)C(=O)c1cc(ccc1F)c1n[nH]c(=O)n1C)O | 0.852 | 0.801 | -1.027 | 1 | 0.405 | 2.940 | -8.793 |
| COCC(=O)N1CCN(CC1)C(=O)c1cc(ccc1F)Cc1n[nH]c(=O)c(=O)n1C | 0.852 | 0.630 | -0.989 | 1 | 0.500 | 2.548 | -6.812 |
| CCN1CCN(CC1)C(=O)c1cc(ccc1F)c1n[nH]c(=O)n1C | 0.851 | 0.897 | -0.950 | 1 | 0.389 | 2.373 | -8.274 |
| OCCN1CCN(CC1)C(=O)c1cc(ccc1F)Cc1n[nH]c(=O)n1C | 0.850 | 0.753 | -1.074 | 1 | 0.478 | 2.456 | -6.998 |
| CNN1CCN(C1)C(=O)c1cc(ccc1F)Cc1n[nH]c(=O)n1C | 0.850 | 0.801 | -1.082 | 1 | 0.451 | 2.950 | -6.907 |
| CN1CCN(CC1)C(=O)c1cc(ccc1F)Cc1nn(c(=O)n1C)C | 0.849 | 0.798 | -1.089 | 1 | 0.441 | 2.439 | -8.824 |
| O=C1CCN(C1)CCN1CCN(C1)C(=O)Cc1n[nH]c(=O)c2c1cccc2 | 0.861 | 0.788 | -0.821 | 1 | 0.397 | 2.700 | -10.009 |
| OCCN1CCN(CC1)C(=O)C1CN(C1)Cc1n[nH]c(=O)c2c1cccc2 | 0.852 | 0.736 | -1.025 | 1 | 0.507 | 2.433 | -11.238 |
| OCCN1CCN(CC1)CC(=O)C1CN(C1)Cc1n[nH]c(=O)c2c1cccc2 | 0.851 | 0.666 | -1.053 | 1 | 0.403 | 2.587 | -9.820 |
| FCCN1CCN(CC1)C(=O)C(=O)N1CCN(CC1)Cc1n[nH]c(=O)c2c1cccc2 | 0.849 | 0.669 | -1.087 | 1 | 0.433 | 2.624 | -8.254 |
| OCCN1CCN(CC1)C(=O)C(=O)N1CCC(C1)Cc1n[nH]c(=O)c2c1cccc2 | 0.849 | 0.651 | -1.068 | 1 | 0.411 | 3.063 | -10.398 |
| O=C(N1CCN(C1)C(=O)CN1CCOCC1)C1CN(C1)Cc1n[nH]c(=O)c2c1cccc2 | 0.848 | 0.657 | -1.105 | 1 | 0.434 | 2.735 | -8.805 |
| O=C(C(=O)N1CCOCC1)N1CCN(CC1)Cc1n[nH]c(=O)c2c1cccc2 | 0.848 | 0.695 | -1.108 | 1 | 0.412 | 2.461 | -9.607 |
| OCCN1CCN(C(=O)C1)C1CN(C1)Cc1n[nH]c(=O)c2c1cccc2 | 0.848 | 0.731 | -0.918 | 1 | 0.378 | 2.641 | -11.468 |
| OCCN1CC(C1)C(=O)N1CCN(CC1)Cc1n[nH]c(=O)c2c1cccc2 | 0.847 | 0.736 | -1.126 | 1 | 0.485 | 2.437 | -9.153 |
| CC(=O)N(CCN1CCN(CC1)C(=O)c1n[nH]c(=O)c2c1cccc2)C | 0.844 | 0.842 | -1.087 | 1 | 0.389 | 2.299 | -9.293 |
| CC(=O)N1CCN(CC1)CCN1CCN(CC1)C(=O)c1n[nH]c(=O)c2c1cccc2 | 0.840 | 0.751 | -1.271 | 1 | 0.424 | 2.276 | -8.980 |
| O=C(N1CCN(CC1)C(=O)C)CN1CCN(CC1)C(=O)c1n[nH]c(=O)c2c1cccc2 | 0.840 | 0.700 | -1.275 | 1 | 0.406 | 2.288 | -10.733 |
| O=C(N1CCN(C1=O)C(=O)C)C1CCN(CC1)Cc1n[nH]c(=O)c2c1cccc2 | 0.836 | 0.826 | -1.370 | 1 | 0.451 | 2.677 | -7.019 |
| CC(=O)N1CCN(CC1)C(=O)c1n[nH]c(=O)c2c1cccc2 | 0.835 | 0.816 | -1.375 | 1 | 0.435 | 2.049 | -10.079 |
| CC(=O)N(CCN1CCN(C1)C(=O)Cc1n[nH]c(=O)c2c1cccc2)C | 0.834 | 0.816 | -1.267 | 1 | 0.387 | 2.617 | -10.473 |
| CC(=O)N1CCN(CC1)CCc1n[nH]c(=O)c2c1cccc2 | 0.834 | 0.902 | -1.403 | 1 | 0.431 | 2.192 | -9.707 |
| CC(=O)N(CCN1CCN(C1)C(=O)c1n[nH]c(=O)c2c1cccc2)C | 0.834 | 0.851 | -1.200 | 1 | 0.378 | 2.541 | -9.975 |
| O=C(c1n[nH]c(=O)c2c1cccc2)N1CCN(CC1)S(=O)(=O)O | 0.833 | 0.710 | -1.415 | 1 | 0.409 | 2.303 | -8.855 |
| CC(=O)N1CCN(CC1)Cc1n[nH]c(=O)c2c1cccc2 | 0.833 | 0.877 | -1.427 | 1 | 0.444 | 2.148 | -9.535 |
| OCCN1CCN(CC1)CCN1CCN(C1)C(=O)c1n[nH]c(=O)c2c1cccc2 | 0.846 | 0.659 | -1.154 | 1 | 0.406 | 2.501 | -7.512 |
| OC(=O)CN1CCN(C1)CCN1CCN(C1)C(=O)c1n[nH]c(=O)c2c1cccc2 | 0.844 | 0.654 | -1.136 | 1 | 0.394 | 2.718 | -9.957 |
| O=CN1CCN1CCN1CCN(CC1)C(=O)c1n[nH]c(=O)c2c1cccc2 | 0.843 | 0.705 | -1.060 | 1 | 0.384 | 2.884 | -10.131 |
| O=CN1CCN(CC1)CCN1CCN(C1)C(=O)c1n[nH]c(=O)c2c1cccc2 | 0.842 | 0.693 | -1.189 | 1 | 0.394 | 2.681 | -10.278 |
| O=NN1CCN(CC1)CCN1CCN(C1)C(=O)c1n[nH]c(=O)c2c1cccc2 | 0.840 | 0.719 | -1.284 | 1 | 0.414 | 2.756 | -9.677 |
| OCCN1CN(C1)CCN1CCN(CC1)C(=O)c1n[nH]c(=O)c2c1cccc2 | 0.840 | 0.671 | -1.285 | 1 | 0.406 | 2.495 | -8.744 |
| OCCN1CCN(CC1)CCN1CCN(CC1)C(=O)c1n[nH]c(=O)c2c1cccc2 | 0.839 | 0.645 | -1.291 | 1 | 0.424 | 2.309 | -9.575 |
| O=C(N1CC1)CN1CCN(CC1)Cc1n[nH]c(=O)c2c1cccc2 | 0.845 | 0.793 | -1.177 | 1 | 0.431 | 2.180 | -9.119 |
| OC1CN(C1)C(=O)CN1CCN(CC1)Cc1n[nH]c(=O)c2c1cccc2 | 0.842 | 0.748 | -1.246 | 1 | 0.400 | 2.381 | -9.774 |
| O=C1CN(C1)CCN1CCN(CC1)C(=O)c1n[nH]c(=O)c2c1cccc2 | 0.840 | 0.800 | -1.278 | 1 | 0.418 | 2.372 | -10.435 |
| O=C(C(=O)N1CCNCC1)N1CCN(CC1)Cc1n[nH]c(=O)c2c1cccc2 | 0.838 | 0.644 | -1.312 | 1 | 0.418 | 2.546 | -9.322 |
| OCCN1CCC1C(=O)N1CCN(CC1)C(=O)Cc1n[nH]c(=O)c2c1cccc2 | 0.838 | 0.682 | -1.323 | 1 | 0.444 | 2.965 | -11.409 |
| O=C(N1CCN(CC1)CC(=O)N1COCC1)Cc1n[nH]c(=O)c2c1cccc2 | 0.837 | 0.757 | -1.126 | 1 | 0.378 | 2.517 | -9.754 |
| OCCN1CCN(CC1)C(=O)C(=O)N1CCN(CC1)Cc1n[nH]c(=O)c2c1cccc2 | 0.837 | 0.582 | -1.069 | 1 | 0.412 | 2.524 | -8.419 |
| OCCN1CCN(CC1)C(=O)CN1CCN(CC1)Cc1n[nH]c(=O)c2c1cccc2 | 0.837 | 0.637 | -1.284 | 1 | 0.394 | 2.378 | -10.131 |
| OCCN1CCN(CC1=O)Cc1ccc(c(c1)C(=O)N1CCN(CC1)CC(O)C)F | 0.873 | 0.622 | -0.366 | 1 | 0.385 | 2.903 | -7.749 |
| CC(CN1CCN(CC1)C(=O)c1cc(ccc1F)CN1CCN(C(=O)C1)C)O | 0.869 | 0.778 | -0.681 | 1 | 0.405 | 2.816 | -7.213 |
| CC(CN1CCN(CC1)C(=O)c1cc(ccc1F)CN1CCNC(=O)C1=O)O | 0.868 | 0.655 | -0.712 | 1 | 0.432 | 2.956 | -10.075 |
| CC(CN1CCN(CC1)C(=O)c1cc(ccc1F)CN1CC(=O)N(C1)C)O | 0.866 | 0.793 | -0.746 | 1 | 0.405 | 2.950 | -8.022 |
| O=CN1CCN(CC1)C(=O)C1CN(C1)C(=O)c1n[nH]c(=O)c2c1cccc2 | 0.842 | 0.720 | -1.238 | 1 | 0.456 | 2.549 | -10.799 |
| CCN1CCN(C1)C(=O)C1CN(C1)C(=O)c1n[nH]c(=O)c2c1cccc2 | 0.842 | 0.845 | -1.247 | 1 | 0.437 | 2.541 | -10.330 |
| OCN1CCN1CCN1CCN(CC1)C(=O)c1n[nH]c(=O)c2c1cccc2 | 0.835 | 0.707 | -1.382 | 1 | 0.400 | 2.819 | -10.173 |
| O=CN1CCN(CC1)C(=O)C1CCN(C1)C(=O)c1n[nH]c(=O)c2c1cccc2 | 0.835 | 0.732 | -1.388 | 1 | 0.457 | 2.932 | -11.055 |
| OCC(=O)N1CCN(CC1)C(=O)C1CN(C1)C(=O)c1n[nH]c(=O)c2c1cccc2 | 0.833 | 0.658 | -1.424 | 1 | 0.471 | 2.424 | -9.280 |
| O=CN1CCN(CC1)C(=O)N1CCN(CC1)C(=O)c1n[nH]c(=O)c2c1cccc2 | 0.832 | 0.691 | -1.447 | 1 | 0.409 | 2.528 | -10.668 |
| O=CN1CCN(C1)C(=O)C1CCN(C1)C(=O)c1n[nH]c(=O)c2c1cccc2 | 0.831 | 0.744 | -1.379 | 1 | 0.425 | 3.138 | -11.011 |
| O=CN1CCN(CC1)C(=O)C1CCN(CC1)C(=O)c1ncc(c(=O)[nH]1)F | 0.831 | 0.696 | -1.282 | 1 | 0.380 | 2.710 | -7.787 |
| OCCn1c(n[nH]c1=O)Cc1ccc(c(c1)C(=O)N1CCN(CC1)C=O)F | 0.831 | 0.641 | -1.472 | 1 | 0.459 | 2.734 | -9.576 |
| OCCN1CCN(CC1)C(=O)c1cc(ccc1F)CN1C(=O)N(C1=O)CCO | 0.850 | 0.671 | -1.069 | 1 | 0.400 | 2.497 | -8.060 |
| OCCN1CCN(CC1)C(=O)C1CN1C(=O)c1n[nH]c(=O)c2c1cccc2 | 0.842 | 0.659 | -1.172 | 1 | 0.417 | 3.119 | -10.815 |
| OCC(=O)N1CCN(CC1)CCN1CCN(C1)C(=O)c1n[nH]c(=O)c2c1cccc2 | 0.839 | 0.630 | -1.192 | 1 | 0.394 | 2.561 | -9.666 |
| NC(=O)CN1CCN(C1)CCN1CCN(CC1)C(=O)c1n[nH]c(=O)c2c1cccc2 | 0.837 | 0.607 | -1.191 | 1 | 0.429 | 2.545 | -11.186 |
| O=C(C(=O)N1CCN(CC1)CCc1n[nH]c(=O)c2c1cccc2)N1CCNCC1 | 0.837 | 0.646 | -1.350 | 1 | 0.406 | 2.581 | -10.078 |
| CC1N(CCNC1=O)CCN1CCN(CC1)C(=O)c1n[nH]c(=O)c2c1cccc2 | 0.836 | 0.720 | -1.179 | 1 | 0.382 | 2.952 | -10.228 |
| O=C1NCCN1CCN1CCN(CC1)C(=O)c1n[nH]c(=O)c2c1cccc2 | 0.835 | 0.770 | -1.324 | 1 | 0.394 | 2.421 | -9.335 |
| [O-]C(=O)CCN1CCN(CC1)C(=O)c1cc(ccc1F)Cc1n[nH]c(=O)n1C | 0.865 | 0.658 | -0.769 | 1 | 0.479 | 2.821 | -7.886 |
| OCCn1nc(n(c1=O)C)Cc1ccc(c(c1)C(=O)N1CCN(CC1)C(=O)[O-])F | 0.856 | 0.638 | -0.936 | 1 | 0.400 | 2.874 | -6.963 |
| [O-]C(=O)C(N1CCN(CC1)C(=O)c1cc(ccc1F)Cc1nn(c(=O)n1C)C)C | 0.855 | 0.617 | -0.658 | 1 | 0.395 | 3.290 | -8.449 |
| OCCn1nnnc1Cc1ccc(c(c1)C(=O)N1CCN(CC1)C(=O)[O-])F | 0.854 | 0.671 | -0.913 | 1 | 0.392 | 2.789 | -7.734 |
| CCOC(=O)N(N1CCN(CC1)C(=O)c1cc(ccc1F)Cc1n[nH]c(=O)n1C)C | 0.853 | 0.760 | -1.008 | 1 | 0.423 | 2.880 | -7.280 |
| OCC(=O)N1CCN(CC1=O)C(=O)c1cc(ccc1F)Cc1ncc(=O)[nH]n1 | 0.856 | 0.663 | -0.942 | 1 | 0.429 | 2.865 | -8.698 |
| OCN(C(=O)N1CCN(CC1=O)C(=O)c1cc(ccc1F)Cc1n[nH]c(=O)n1C)C | 0.847 | 0.618 | -0.991 | 1 | 0.400 | 3.061 | -8.948 |
| OCC(=O)N1CCN(CC1=O)C(=O)c1cc(ccc1F)Cc1nncc(=O)[nH]1 | 0.846 | 0.663 | -1.127 | 1 | 0.397 | 2.988 | -7.365 |
| OCC(=O)N1CCN(CC1)C(=O)c1cc(CCc2n[nH]c(=O)n2C)ccc1F | 0.845 | 0.701 | -1.172 | 1 | 0.431 | 2.525 | -8.518 |
| OCC(=O)N1CCN(CC1)C(=O)c1cc(ccc1F)Cc1n[nH]c(=O)c(=O)n1C | 0.845 | 0.594 | -0.962 | 1 | 0.514 | 2.580 | -7.845 |
| OCC(=O)CN1CCN(CC1=O)C(=O)c1cc(ccc1F)Cc1ncc(=O)[nH]n1 | 0.844 | 0.614 | -1.085 | 1 | 0.418 | 2.919 | -8.522 |
| OCCC(=O)N1CCN(CC1=O)C(=O)c1cc(ccc1F)Cc1n[nH]c(=O)n1C | 0.843 | 0.659 | -1.220 | 1 | 0.410 | 2.808 | -7.247 |
| [O-]C(=O)Cc1ccc(c(c1)C(=O)N1CCN(CC1)C(=O)C)F | 0.862 | 0.748 | -0.818 | 1 | 0.406 | 2.421 | -6.706 |
| O=C(c1cc(ccc1F)CC(=O)[O-])N1CCN(CC1)CC(=O)N1CCOCC1 | 0.861 | 0.621 | -0.477 | 1 | 0.370 | 2.520 | -6.898 |
| OC(=O)CN1CCN(CC1)C(=O)c1cc(ccc1F)CC(=O)[O-] | 0.856 | 0.743 | -0.950 | 1 | 0.403 | 2.438 | -6.681 |
| CC(CN1CCN(CC1)C(=O)c1cc(ccc1F)CC(=O)[O-])O | 0.856 | 0.771 | -0.956 | 1 | 0.426 | 2.976 | -7.593 |
| CCN1CCN(CC1)C(=O)c1cc(ccc1F)CC(=O)[O-] | 0.855 | 0.777 | -0.973 | 1 | 0.409 | 2.407 | -6.381 |
| [O-]C(=O)CCc1ccc(c(c1)C(=O)N1CCN(CC1)C(=O)C)F | 0.852 | 0.770 | -0.913 | 1 | 0.388 | 2.428 | -7.386 |
| [O-]C(=O)CCN1CCN(CC1)C(=O)c1cc(ccc1F)CC(=O)[O-] | 0.852 | 0.596 | -0.823 | 1 | 0.424 | 2.871 | -6.910 |
| OCC(=O)N1CCN(CC1)C(=O)c1cc(ccc1F)CN1CCOCC1 | 0.847 | 0.809 | -0.814 | 1 | 0.366 | 2.164 | -7.207 |
| O=CCN1CCN(CC1)C(=O)c1cc(ccc1F)CC(=O)[O-] | 0.847 | 0.657 | -1.142 | 1 | 0.418 | 2.645 | -6.461 |
| OCCN1CCN(CC1)Cc1ccc(c(c1)C(=O)N1CCS(=O)(=O)CC1)F | 0.845 | 0.739 | -0.807 | 1 | 0.361 | 2.393 | -8.046 |
| OCCN1CCN(C1)C(=O)c1cc(ccc1F)Cc1nccn(c1=O)CCO | 0.844 | 0.672 | -1.204 | 1 | 0.440 | 2.800 | -9.160 |
| OCCN1CCN(CC1)C(=O)c1cc(ccc1F)Cc1cnn(c(=O)c1F)CCO | 0.844 | 0.644 | -1.205 | 1 | 0.452 | 2.679 | -7.804 |
| OCCN1CCN(CC1)C(=O)c1cc(ccc1F)CN1CN(CCO)CCC1=O | 0.843 | 0.636 | -1.167 | 1 | 0.397 | 2.528 | -8.531 |
| OCCN1CCN(C1)C(=O)c1cc(ccc1F)CN1CCN(C(=O)C1)CCO | 0.842 | 0.628 | -0.928 | 1 | 0.373 | 2.608 | -7.533 |
| OCCN1CCN(CC1)C(=O)c1cc(ccc1F)CC1CCC(=O)N(C1=O)CCO | 0.841 | 0.595 | -1.038 | 1 | 0.400 | 2.966 | -7.636 |
| OCCN1CCN2C(C1=O)CN(CC2)C(=O)c1cc(ccc1F)c1n[nH]c2c1cccc2 | 0.824 | 0.643 | -1.565 | 1 | 0.402 | 3.069 | -8.365 |
| O=C1CN(CCN1CCN1CCOCC1)C(=O)c1cc(ccc1F)Cc1n[nH]ccc1=O | 0.824 | 0.681 | -1.607 | 1 | 0.413 | 2.888 | -7.330 |
| OCCN1CCN2C(C1=O)CN(CC2)C(=O)c1n[nH]c(=O)c2c1cccc2 | 0.821 | 0.708 | -1.365 | 1 | 0.368 | 2.982 | -9.904 |
| O=c1[nH]ncc(c1)C(=O)N1CCN(CC1)C(=O)c1ccccc1F | 0.819 | 0.869 | -1.716 | 1 | 0.403 | 2.155 | -6.884 |
| OCCN1C(=O)CC(C1=O)CN1CCN(CC1)C(=O)c1cc(F)ccc1F | 0.818 | 0.732 | -1.136 | 1 | 0.338 | 2.905 | -6.852 |
| OCC[NH2+]c1cccnc1Cc1ccc(c(c1)C(=O)N1CCN(C(=O)C1)CCO)F | 0.814 | 0.525 | -1.032 | 1 | 0.395 | 3.335 | -7.778 |
| OCCN1CCN(CC1=O)C(=O)c1cc(ccc1F)Cc1n[nH]ccc1=O | 0.813 | 0.752 | -1.836 | 1 | 0.440 | 2.860 | -7.852 |
| OCCN1CCN(CC1C(=O)O)C(=O)c1cc(ccc1F)Cc1ncccc1F | 0.812 | 0.749 | -1.841 | 1 | 0.416 | 3.015 | -8.518 |
| OCCn1c(=O)[nH]nc(c1=O)Cc1ccc(c(c1)C(=O)N1CCN(C1)C(=O)C)F | 0.854 | 0.651 | -0.988 | 1 | 0.473 | 2.807 | -8.240 |
| CC(=O)N1CCN(CC1)C(=O)c1cc(ccc1F)Cc1nc(=O)n([nH]c1=O)O | 0.847 | 0.660 | -1.129 | 1 | 0.500 | 2.840 | -7.940 |
| O=C(N1CN(C1)C(=O)C)N1CCN(C1)C(=O)c1cc(ccc1F)Cc1n[nH]c(=O)n1C | 0.847 | 0.720 | -1.124 | 1 | 0.432 | 3.033 | -8.402 |
| OCCn1[nH]c(=O)c(nc1=O)Cc1ccc(c(c1)C(=O)N1CCN(CC1)C(=O)C)F | 0.846 | 0.637 | -1.148 | 1 | 0.472 | 2.765 | -8.097 |
| OCCn1c(Cc2ccc(c(c2)C(=O)N2CCN(CC2)C(=O)C)F)nc(=O)[nH]c1=O | 0.844 | 0.637 | -1.188 | 1 | 0.408 | 2.633 | -9.118 |
| OCCn1[nH]nc(c(=O)c1=O)Cc1ccc(c(c1)C(=O)N1CCN(CC1)C(=O)C)F | 0.843 | 0.596 | -1.000 | 1 | 0.472 | 2.881 | -8.077 |
| OCCn1c(n[nH]c(=O)c1=O)Cc1ccc(c(c1)C(=O)N1CCN(CC1)C(=O)C)F | 0.841 | 0.596 | -1.049 | 1 | 0.507 | 2.572 | -8.244 |
| OCCN(C1CCN(C1)C(=O)c1cc(ccc1F)Cc1n[nH]c(=O)n1C)C(=O)C | 0.841 | 0.697 | -1.146 | 1 | 0.423 | 3.209 | -9.038 |
| OCCN1CCN(CC1)Cc1ccc(c(c1)C(=O)N1CCN(CC1)C(=O)C)F | 0.855 | 0.778 | -0.890 | 1 | 0.391 | 2.148 | -7.368 |
| OCCN1CCN(CC1)C(=O)c1cc(ccc1F)CN1CCN1CCO | 0.854 | 0.685 | -0.971 | 1 | 0.397 | 2.709 | -7.791 |
| OCCn1c(c[nH]c1=O)Cc1ccc(c(c1)C(=O)N1CCN(CC1)C(=O)[O-])F | 0.863 | 0.677 | -0.798 | 1 | 0.400 | 2.995 | -7.259 |
| OCCN1CCN(CC1)Cc1ccc(c(c1)C(=O)N1CCN(CC1)C(=O)[O-])F | 0.860 | 0.693 | -0.739 | 1 | 0.386 | 2.487 | -7.033 |
| OCCn1cc([nH]c1=O)Cc1ccc(c(c1)C(=O)N1CCN(CC1)C(=O)[O-])F | 0.856 | 0.677 | -0.946 | 1 | 0.405 | 2.943 | -7.448 |
| OCCN1CCN(CC1)CCc1ccc(c(c1)C(=O)N1CCN(CC1)C(=O)[O-])F | 0.856 | 0.656 | -0.824 | 1 | 0.386 | 2.535 | -8.405 |
| [O-]C(=O)N1CCN(CC1)CCc1ccc(c(c1)C(=O)N1CCN(CC1)C(=O)[O-])F | 0.855 | 0.598 | -0.771 | 1 | 0.409 | 2.833 | -6.641 |
| OCCN1CCN(CC1)Cc1ccc(c(c1)C(=O)N1CCN(CC1)CO)F | 0.853 | 0.703 | -0.988 | 1 | 0.397 | 2.275 | -7.173 |
| OCCN1CCN(CC1)Cc1ccc(c(c1)C(=O)N1CCN(CC1)C(=O)O)F | 0.853 | 0.751 | -0.937 | 1 | 0.391 | 2.176 | -7.033 |
| O=C(C(=C)N1CCN(C1)C(=O)c1cc(ccc1F)Cc1ncc(=O)[nH]n1)N(C)C | 0.860 | 0.720 | -0.803 | 1 | 0.423 | 3.134 | -7.385 |
| O=C(C(=C)N1CCN(CC1)C(=O)c1cc(ccc1F)Cc1ncc(=O)[nH]n1)N(C)C | 0.856 | 0.704 | -0.946 | 1 | 0.453 | 2.964 | -8.132 |
| CN1CCN(CC1)C(=O)C(=O)N1CCN(CC1)C(=O)c1cc(ccc1F)Cc1n[nH]cn1 | 0.856 | 0.647 | -0.954 | 1 | 0.437 | 2.876 | -6.433 |
| O=C(C(=C)N1CCN(C1)C(=O)c1cc(ccc1F)Cc1n[nH]c(=O)[nH]1)N1CCOCC1 | 0.856 | 0.641 | -0.858 | 1 | 0.410 | 3.174 | -7.799 |
| NC(=O)C(=O)N1CCN(CC1)C(=O)c1cc(ccc1F)Cc1ncc(=O)[nH]n1 | 0.855 | 0.624 | -0.899 | 1 | 0.479 | 2.669 | -8.728 |
| O=C(N(N1CCN(C1)C(=O)c1cc(ccc1F)Cc1ncc(=O)[nH]n1)C)N(C)C | 0.855 | 0.779 | -0.863 | 1 | 0.418 | 3.209 | -8.347 |
| O=C(C(=O)N1CCN(CC1)C(=O)c1cc(ccc1F)Cc1ncc(=O)[nH]n1)N(C)C | 0.850 | 0.659 | -1.067 | 1 | 0.466 | 2.747 | -7.843 |
| CN(C(=O)C(=O)N1CCN(CC1)C(=O)c1cc(ccc1F)CN1CCNC1=O)C | 0.849 | 0.701 | -0.949 | 1 | 0.384 | 2.577 | -7.664 |
| COCCN1CCN(C1)C(=O)c1cc(ccc1F)Cc1n[nH]c(=O)c(=O)n1C | 0.845 | 0.670 | -1.180 | 1 | 0.480 | 2.725 | -8.252 |
| CNC(=O)N1CCN(CC1)C(=O)c1cc(ccc1F)CN1CCC1=O | 0.866 | 0.816 | -0.741 | 1 | 0.400 | 2.258 | -6.691 |
| OCCc1ccc(c(c1)C(=O)N1CCN(CC1)CC(=O)N1CC1)F | 0.864 | 0.767 | -0.784 | 1 | 0.403 | 2.048 | -7.163 |
| OCC(CN1CCN(CC1)C(=O)c1cc(ccc1F)CN1CCN(CC1)C(=O)C)O | 0.862 | 0.649 | -0.728 | 1 | 0.389 | 2.713 | -7.915 |
| OCN1CCN(CC1)Cc1ccc(c(c1)C(=O)N1CCN(CC1)C(=O)CO)F | 0.861 | 0.678 | -0.814 | 1 | 0.397 | 2.341 | -7.916 |
| O=C(c1cc(ccc1F)CN1CC[NH2+]CC1)N1CCN(CC1)CC(=O)N1CC1 | 0.861 | 0.642 | -0.657 | 1 | 0.380 | 2.950 | -7.137 |
| CN(N1CCN(CC1)C(=O)c1cc(ccc1F)Cc1nn(c(=O)n1C)C)C | 0.863 | 0.756 | -0.799 | 1 | 0.417 | 2.790 | -7.571 |
| CC(N1CCN(CC1)C(=O)c1cc(ccc1F)CN1CCN(C(=O)C1)C)O | 0.859 | 0.811 | -0.602 | 1 | 0.368 | 2.880 | -7.524 |
| OCN1CCN(CC1)C(=O)c1cc(ccc1F)Cc1n[nH]c(=O)n(c1=O)[O-] | 0.859 | 0.676 | -0.890 | 1 | 0.544 | 2.998 | -7.632 |
| OCCN1CCN(CC1)C(=O)c1cc(ccc1F)Cc1n[nH]c(=O)n(c1=O)[O-] | 0.858 | 0.650 | -0.915 | 1 | 0.529 | 2.877 | -7.473 |
| OCCN1CCN(CC1)C(=O)c1cc(ccc1F)Cn1[nH]c(=O)n(c1=O)[O-] | 0.848 | 0.658 | -1.108 | 1 | 0.405 | 2.915 | -8.129 |
| OCCN1CCN(CC1)C(=O)c1cc(ccc1F)Cc1n[nH]c(=O)c(cc1=O)[O-] | 0.845 | 0.634 | -1.152 | 1 | 0.559 | 2.948 | -6.974 |
| OCCN1CCN(CC1=O)C(=O)c1cc(ccc1F)Cc1n[nH]c(=O)n1[O-] | 0.844 | 0.680 | -1.194 | 1 | 0.429 | 2.992 | -10.029 |
| OCCn1c(n[nH]c1=O)Cc1ccc(c(c1)C(=O)N1CCN(CC1)C(=O)[O-])F | 0.843 | 0.629 | -1.159 | 1 | 0.452 | 2.848 | -9.027 |
| OCCN1CCN(CC1)C(=O)c1cc(ccc1F)Cn1[nH]c([O-])cc(=O)c1=O | 0.843 | 0.602 | -1.036 | 1 | 0.400 | 2.924 | -7.153 |
| OCCN1CCN(CC1)C(=O)c1cc(ccc1F)Cc1n[nH]n(c(=O)c1=O)[O-] | 0.843 | 0.608 | -1.055 | 1 | 0.486 | 3.024 | -6.834 |
| OCCn1c(=O)[nH]nc(c1=O)Cc1ccc(c(c1)C(=O)N1CCN(CC1)[O-])F | 0.842 | 0.660 | -1.224 | 1 | 0.500 | 3.015 | -7.880 |
| CCC(=O)N1CCN(CC1)C(=O)c1cc(ccc1F)Cc1n[nH]c(=O)n1CC(=O)[O-] | 0.861 | 0.623 | -0.771 | 1 | 0.473 | 2.863 | -9.107 |
| O=C(N1CCOCC1)N1CCN(C1)C(=O)c1cc(ccc1F)Cc1n[nH]c(=O)n1C | 0.857 | 0.756 | -0.939 | 1 | 0.421 | 2.806 | -8.402 |
| OCCn1cnc(=O)c(n1)Cc1ccc(c(c1)C(=O)N1CCN(C1)C(=O)[O-])F | 0.855 | 0.652 | -0.910 | 1 | 0.403 | 3.124 | -8.005 |
| OCC(=O)N1CCN(CC1)Cc1ccc(c(c1)C(=O)N1CCN(CC1)C(=O)[O-])F | 0.854 | 0.655 | -0.859 | 1 | 0.386 | 2.548 | -6.775 |
| CC(=O)C1CN(C1)C(=O)CN1CCN(C1)C(=O)c1cc(ccc1F)Cc1nnn[nH]1 | 0.868 | 0.688 | -0.657 | 1 | 0.395 | 2.986 | -7.251 |
| O=C1CN(C1)C(=O)CN1CCN(C1)C(=O)c1cc(ccc1F)Cc1nnn[nH]1 | 0.867 | 0.704 | -0.720 | 1 | 0.403 | 3.008 | -6.927 |
| O=C1CN(C1)C1CN(C1)CCN1CCN(C1)C(=O)c1cc(ccc1F)Cc1nnn[nH]1 | 0.864 | 0.613 | -0.593 | 1 | 0.413 | 3.128 | -6.883 |
| O=C1CN(C1)CCN1CCN(C1)C(=O)c1cc(ccc1F)Cc1n[nH]nn1 | 0.863 | 0.731 | -0.810 | 1 | 0.472 | 2.857 | -7.146 |
| O=C1CN(C1)CCN1CCN(C1)C(=O)c1cc(ccc1F)CCc1nnn[nH]1 | 0.861 | 0.698 | -0.839 | 1 | 0.408 | 2.979 | -6.803 |
| O=C1CN(C1)C(=O)CN1CCN(C1)C(=O)c1cc(ccc1F)Cc1ncn[nH]1 | 0.860 | 0.754 | -0.844 | 1 | 0.397 | 2.880 | -8.048 |
| O=C1CN(C1)C1CCN(C1)C(=O)c1cc(ccc1F)Cc1nnn[nH]1 | 0.859 | 0.841 | -0.740 | 1 | 0.421 | 3.281 | -6.642 |
| OCC1CN(C1)CCN1CCN(C1)C(=O)c1cc(ccc1F)Cc1nnn[nH]1 | 0.859 | 0.663 | -0.898 | 1 | 0.429 | 2.934 | -7.490 |
| O=C1CN(C1)CCN1CCN(C1)C1CN(C1)C(=O)c1cc(ccc1F)Cc1nnn[nH]1 | 0.858 | 0.613 | -0.652 | 1 | 0.395 | 3.138 | -6.872 |
