# Supplementary material for: ChemMORT: an automatic ADMET optimization platform using deep learning and multi-objective particle swarm optimization
Source: Brief Bioinform. 2024 Feb 20;25(2):bbae008. doi: 10.1093/bib/bbae008 (PMC10883642; doi:10.1093/bib/bbae008)
Supplement: supplementary_materials_bbae008 [file supplementary_materials_bbae008.zip › supplementary_materials_bbae008/Table S5.docx]

**Table S5.** The MM-GBSA value of Olaparib and optimized molecules

| **Ligand** | **MM-GBSA (kcal/mol)** |
| --- | --- |
| Olaparib | -52.130 |
| Optimized molecule 1 | -39.569 |
| Optimized molecule 2 | -39.377 |
| Optimized molecule 3 | -56.772 |
| Optimized molecule 4 | -58.949 |
| Optimized molecule 5 | -58.831 |
