# Supplementary material for: ChemMORT: an automatic ADMET optimization platform using deep learning and multi-objective particle swarm optimization
Source: Brief Bioinform. 2024 Feb 20;25(2):bbae008. doi: 10.1093/bib/bbae008 (PMC10883642; doi:10.1093/bib/bbae008)
Supplement: supplementary_materials_bbae008 [file supplementary_materials_bbae008.zip › supplementary_materials_bbae008/Table S6.docx]

**Table S6.** Browser compatibility data for ChemMORT

| **OS** | **Version** | **Chrome** | **Edge** | **Firefox** | **Safari** |
| --- | --- | --- | --- | --- | --- |
| Linux | Ubuntu 18.04.5 LTS | 87.0.4280.141 | n/a | 82.0.2 | n/a |
| Windows | 10 | 119.0.6045.200 | 119.0.2151.97 | 120.0.1 | n/a |
| MacOS | Catalina 10.15.6 | 119.0.6045.199 | 119.0.2151.97 | n/a | 17.1.2 |
