# Supplementary figures and images for: ChemMORT: an automatic ADMET optimization platform using deep learning and multi-objective particle swarm optimization
Source: Brief Bioinform. 2024 Feb 20;25(2):bbae008. doi: 10.1093/bib/bbae008 (PMC10883642; doi:10.1093/bib/bbae008)

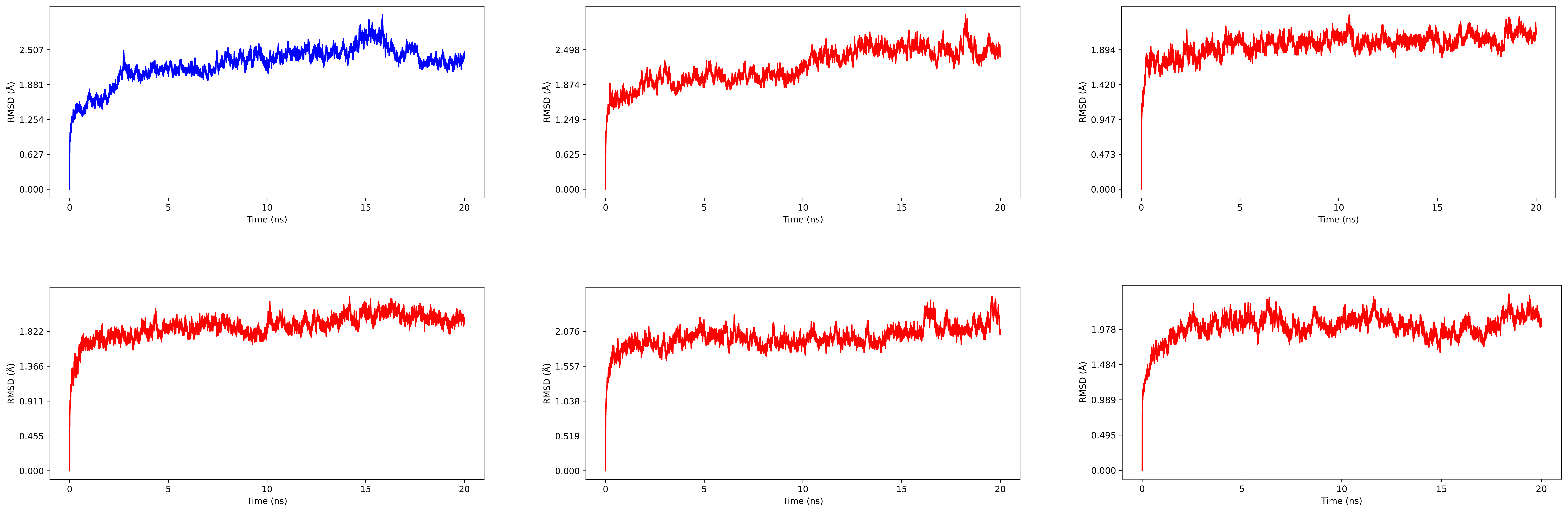

Supplement: supplementary_materials_bbae008 [file supplementary_materials_bbae008.zip › supplementary_materials_bbae008/Figure S1.tif]
